# Towards a theory of cortical columns: From spiking neurons to interacting neural populations of finite size

**DOI:** 10.1371/journal.pcbi.1005507

**Published:** 2017-04-19

**Authors:** Tilo Schwalger, Moritz Deger, Wulfram Gerstner

**Affiliations:** 1 Brain Mind Institute, School of Computer and Communication Sciences and School of Life Sciences, École Polytechnique Fédérale de Lausanne (EPFL), Lausanne, Switzerland; 2 Institute for Zoology, Faculty of Mathematics and Natural Sciences, University of Cologne, Cologne, Germany; Université Paris Descartes, Centre National de la Recherche Scientifique, FRANCE

## Abstract

Neural population equations such as neural mass or field models are widely used to study brain activity on a large scale. However, the relation of these models to the properties of single neurons is unclear. Here we derive an equation for several interacting populations at the mesoscopic scale starting from a microscopic model of randomly connected generalized integrate-and-fire neuron models. Each population consists of 50–2000 neurons of the same type but different populations account for different neuron types. The stochastic population equations that we find reveal how spike-history effects in single-neuron dynamics such as refractoriness and adaptation interact with finite-size fluctuations on the population level. Efficient integration of the stochastic mesoscopic equations reproduces the statistical behavior of the population activities obtained from microscopic simulations of a full spiking neural network model. The theory describes nonlinear emergent dynamics such as finite-size-induced stochastic transitions in multistable networks and synchronization in balanced networks of excitatory and inhibitory neurons. The mesoscopic equations are employed to rapidly integrate a model of a cortical microcircuit consisting of eight neuron types, which allows us to predict spontaneous population activities as well as evoked responses to thalamic input. Our theory establishes a general framework for modeling finite-size neural population dynamics based on single cell and synapse parameters and offers an efficient approach to analyzing cortical circuits and computations.

## Author summary

Understanding the brain requires mathematical models on different spatial scales. On the “microscopic” level of nerve cells, neural spike trains can be well predicted by phenomenological spiking neuron models. On a coarse scale, neural activity can be modeled by phenomenological equations that summarize the total activity of many thousands of neurons. Such population models are widely used to model neuroimaging data such as EEG, MEG or fMRI data. However, it is largely unknown how large-scale models are connected to an underlying microscale model. Linking the scales is vital for a correct description of rapid changes and fluctuations of the population activity, and is crucial for multiscale brain models. The challenge is to treat realistic spiking dynamics as well as fluctuations arising from the finite number of neurons. We obtained such a link by deriving stochastic population equations on the mesoscopic scale of 100–1000 neurons from an underlying microscopic model. These equations can be efficiently integrated and reproduce results of a microscopic simulation while achieving a high speed-up factor. We expect that our novel population theory on the mesoscopic scale will be instrumental for understanding experimental data on information processing in the brain, and ultimately link microscopic and macroscopic activity patterns.

## Introduction

When neuroscientists report electrophysiological, genetic, or anatomical data from a cortical neuron, they typically refer to the cell type, say, a layer 2/3 fast-spiking interneuron, a parvalbumin-positive neuron in layer 5, or a Martinotti cell in layer 4, together with the area, say primary visual cortex or somatosensory cortex [1–4]. Whatever the specific taxonomy used, the fact that a taxonomy is plausible at all indicates that neurons can be viewed as being organized into populations of cells with similar properties. In simulation studies of cortical networks with spiking neurons, the number of different cell types, or neuronal populations, per cortical column ranges from eight [5] to about 200 [6] with 31’000 to 80’000 simulated neurons for one cortical column, but larger simulations of several columns adding up to a million neurons (and 22 cells types) have also been performed [7]. In the following, we will refer to a model where each neuron in each population is simulated explicitly by a spiking neuron model as a *microscopic* model.

On a much coarser level, neural mass models [8–10], also called field models [11–13], population activity equations [14], rate models [15], or Wilson-Cowan models [16] represent the activity of a cortical column at location *x* by one or at most a few variables, such as the mean activity of excitatory and inhibitory neurons located in the region around *x*. Computational frameworks related to neural mass models have been used to interpret data from fMRI [17, 18] and EEG [9]. Since neural mass models give a compact summary of coarse neural activity, they can be efficiently simulated and fit to experimental data [17, 18].

However, neural mass models have several disadvantages. While the stationary state of neural mass activity can be matched to the single-neuron gain function and hence to detailed neuron models [11, 14, 19–22], the dynamics of neural mass models in response to a rapid change in the input does not correctly reproduce a microscopic simulation of a population of neurons [14, 22, 23]. Second, fluctuations of activity variables in neural mass models are either absent or described by an *ad hoc* noise model. Moreover, the links of neural mass models to local field potentials are difficult to establish [24]. Because a systematic link to microscopic models at the level of spiking neurons is missing, existing neural mass models must be considered as heuristic phenomenological, albeit successful, descriptions of neural activity.

In this paper we address the question of whether equations for the activity of populations, similar in spirit but not necessarily identical to Wilson-Cowan equations [16], can be systematically derived from the interactions of spiking neurons at the level of microscopic models. At the microscopic level, we start from generalized integrate-and-fire (GIF) models [14, 25, 26] because, first, the parameters of such GIF models can be directly, and efficiently, extracted from experiments [27] and, second, GIF models can predict neuronal adaptation under step-current input [28] as well as neuronal firing times under in-vivo-like input [26]. In our modeling framework, the GIF neurons are organized into different interacting populations. The populations may correspond to different cell types within a cortical column with known statistical connectivity patterns [3, 6]. Because of the split into different cell types, the number of neurons per population (e.g., fast-spiking inhibitory interneurons in layer 2/3) is finite and in the range of 50–2000 [3]. We call a model at the level of interacting cortical populations of finite size a *mesoscopic* model. The mathematical derivation of the mesoscopic model equations from the microscopic model (i.e. network of generalized integrate-and-fire neurons) is the main topic of this paper. The small number of neurons per population is expected to lead to characteristic fluctuations of the activity which should match those of the microscopic model.

The overall aims of our approach are two-fold. As a first aim, this study would like to develop a theoretical framework for cortical information processing. The main advantage of a systematic link between neuronal parameters and mesoscopic activity is that we can quantitatively predict the effect of changes of neuronal parameters in (or of input to) one population on the activation pattern of this as well as other populations. In particular, we expect that a valid mesoscopic model of interacting cortical populations will become useful to predict the outcome of experiments such as optogenetic stimulation of a subgroup of neurons [29–31]. A better understanding of the activity patterns within a cortical column may in turn, after suitable postprocessing, provide a novel basis for models of EEG, fMRI, or LFP [9, 17, 18, 24]. While we cannot address all these points in this paper, we present an example of nontrivial activity patterns in a network model with stochastic switching between different activity states potentially linked to perceptual bistability [32–34] or resting state activity [18].

As a second aim, this study would like to contribute to multiscale simulation approaches [35] in the neurosciences by providing a new tool for efficient and consistent coarse-grained simulation at the mesoscopic scale. Understanding the computations performed by the nervous system is likely to require models on different levels of spatial scales, ranging from pharmacological interactions at the subcellular and cellular levels to cognitive processes at the level of large-scale models of the brain. Ideally, a modeling framework should be efficient and consistent across scales in the following sense. Suppose, for example, that we are interested in neuronal membrane potentials in one specific group of neurons which receives input from many other groups of neurons. In a microscopic model, all neurons would be simulated at the same level; in a multi-scale simulation approach, only the group of neurons where we study the membrane potentials is simulated at the microscopic level, whereas the input from other groups is replaced by the activity of the mesoscopic model. A multiscale approach is consistent, if the replacement of parts of the microscopic simulation by a mesoscopic simulation does not lead to any change in the observed pattern of membrane potentials in the target population. The approach is efficient if the change of simulation scale leads to a significant speed-up of simulation. While we do not intend to present a systematic comparison of computational performance, we provide an example and measure the speed-up factor between mesoscopic and microscopic simulation for the case of a cortical column consisting of eight populations [5].

Despite of its importance, a quantitative link between mesoscopic population models and microscopic neuronal parameters is still largely lacking. This is mainly due to two obstacles: First, in a cortical column the number of neurons of the same type is small (50–2000 [3]) and hence far from the *N* → ∞ limit of classic “macroscopic” theories in which fluctuations vanish [14, 36–38]. Systematic treatments of finite-size networks using methods from statistical physics (system size expansion [39], path integral methods [40, 41], neural Langevin equations [42–45]) have so far been limited to simplified Markov models that lack, however, a clear connection to single neuron physiology.

Second, spikes generated by a neuron are generally correlated in time due to refractoriness [46], adaptation and other spike history dependencies [28, 47–51]. Therefore spike trains are often not well described by an (inhomogeneous) Poisson process, especially during periods of high firing rates [46]. As a consequence, the mesoscopic population activity (i.e. the sum of spike trains) is generally not simply captured by a Poisson model either [52–54], even in the absence of synaptic couplings [55]. These non-Poissonian finite-size fluctuations on the mesoscopic level in turn imply temporally correlated synaptic input to other neurons (colored noise) that can drastically influence the population activity [53, 54, 56] but which is hard to tackle analytically [57]. Therefore, most theoretical approaches rely on a white-noise or Poisson assumption to describe the synaptic input [58–62], thereby neglecting temporal correlations caused by spike-history dependencies in single neuron activity. Here, we will exploit earlier approaches to treating refractoriness [23] and spike frequency adaptation [55, 63] and combine these with a novel treatment of finite-size fluctuations.

Our approach is novel for several reasons. First, we use generalized integrate-and-fire models that accurately describe neuronal data [25, 26] as our microscopic reference. Second, going beyond earlier studies [58–60, 64], we derive stochastic population equations that account for both strong neuronal refractoriness and finite population size in a consistent manner. Third, our theory has a non-perturbative character as it neither assumes the self-coupling (refractoriness and adaptation) to be weak [65] nor does it hinge on an expansion around the *N* → ∞ solution for large but finite *N* [55, 66, 67]. Thus, it is also valid for relatively small populations and non-Gaussian fluctuations. And forth, in contrast to linear response theories [55, 68–72], our mesoscopic equations work far away from stationary states and reproduce large fluctuations in multistable networks.

In the Results section we present our mesoscopic population equations, suggest an efficient numerical implementation, and illustrate the main dynamical effects via a selected number of examples. To validate the mesoscopic theory we numerically integrate the stochastic differential equations for the mesoscopic population activities and compare their statistics to those of the population activities derived from a microscopic simulation. A detailed account of the derivation is presented in the Methods section. In the discussion section we point out limitations and possible applications of our mesoscopic theory.

## Results

**Fig 1:**
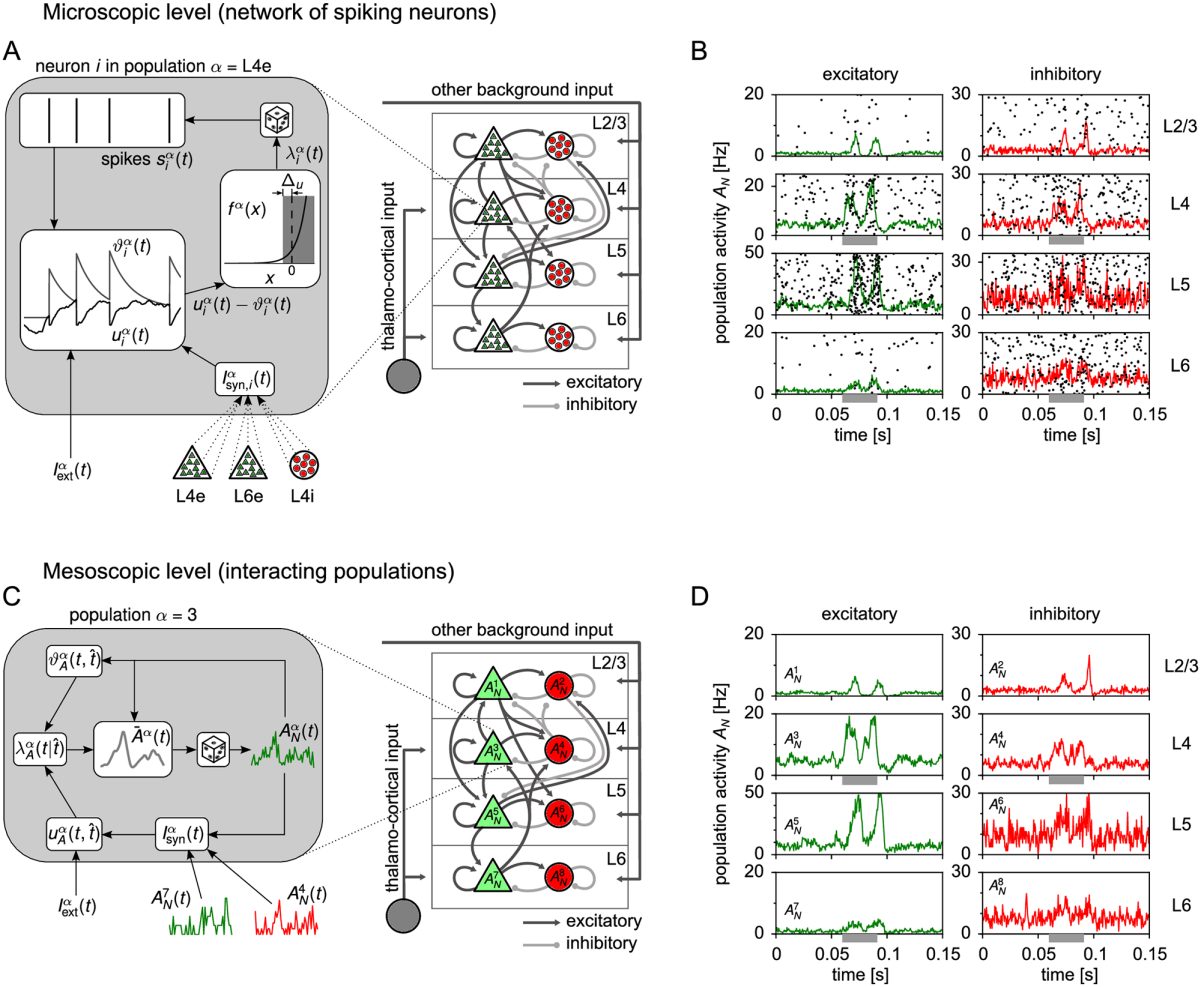
Illustration of population models on the microscopic and mesoscopic level. (A) Cortical column model [5] with ∼ 80’000 neurons organized into four layers (L2/3, L4, L5, L6) each consisting of an excitatory (“e”) and an inhibitory (“i”) population. On the microscopic level, each individual neuron is described by a generalized integrate-and-fire (GIF) model with membrane potential $u_{i}^{\alpha }\left(t\right)$, dynamic threshold $\vartheta_{i}^{\alpha }\left(t\right)$ and conditional intensity $f^{\alpha }(u_{i}^{\alpha }\left(t\right)-\vartheta_{i}^{\alpha }\left(t\right))$. Inset: GIF dynamics for a specific neuron *i* of population L4e (*α* = L4e). The neuron receives spikes from neurons in L4e, L4i and L6e, which drive the membrane potential $u_{i}^{\alpha }\left(t\right)$. Spikes are elicited stochastically by a conditional intensity $\lambda_{i}^{\alpha }\left(t\right)=f^{\alpha }(u_{i}^{\alpha }\left(t\right)-\vartheta_{i}^{\alpha }\left(t\right))$ that depends on the instantaneous difference between $u_{i}^{\alpha }\left(t\right)$ and the dynamic threshold $\vartheta_{i}^{\alpha }\left(t\right)$. Spike feedback (voltage reset and spike-triggered threshold movement) gives rise to spike history effects like refractoriness and adaptation. (B) Spike raster plot of the first 200 neurons of each population. The panels correspond to the populations in (A). Layer 4 and 6 are stimulated by a step current during the interval (0.06s, 0.09s) mimicking thalamic input (gray bars). Solid lines show the population activities $A_{N}^{\alpha }\left(t\right)$ computed with temporal resolution Δ*t* = 0.5 ms, cf. Eq (2). The activities are stochastic due to the finite population size. (C) On the mesoscopic level, the model reduces to a network of 8 populations, each represented by its population activity $A_{N}^{\alpha }\left(t\right)$. Inset: The mesoscopic model generates realizations of $A_{N}^{\alpha }\left(t\right)$ from an expected rate ${\bar{A}}^{\alpha }\left(t\right)$, which is a deterministic functional of the past population activities. (D) A corresponding simulation of the mesoscopic model yields population activities with the same temporal statistics as in (B).

We consider a structured network of interacting homogeneous populations. Homogeneous here means that each population consists of spiking neurons with similar intrinsic properties and random connectivity within and between populations. To define such populations, one may think of grouping neurons into genetically-defined cell classes of excitatory and inhibitory neurons [4], or, more traditionally, into layers and cell types (Fig 1A). For example, pyramidal cells in layer 2/3 of rodent somatosensory cortex corresponding to whisker C2 form a population of about 1700 neurons [3]. Pyramidal cells in layer 5 of the same cortical column form another one (∼1200 neurons [3]), fast-spiking inhibitory cells in layer 2/3 a third one (∼100 neurons [3]) and non-fast-spiking inhibitory cells in layer 2/3 a fourth one (∼130 neurons [3]), and so on [3, 6, 73]. We suppose that the parameters of typical neurons from each population [27, 73, 74], the number of neurons per population [3, 73] and the typical connection probabilities [5] and strengths within and between populations [73, 75–79] are known from experimental data. The resulting spiking neural network can be simulated on a cellular level by numerical integration of the spiking dynamics of each individual neuron (Fig 1B). In the following, we will refer to this level of description as the *microscopic* level. Apart from being computationally expensive, the full microscopic network dynamics is highly complex and hence difficult to understand. To overcome these shortcomings, we have developed a new mean-field description for the mesoscopic dynamics of interacting populations.

### Mesoscopic population equations

A population *α* of size *N*^*α*^ is represented by its population activity $A_{N}^{\alpha }\left(t\right)$ (Greek superscripts label the populations, Fig 1C) defined as

$$\begin{array}{c} A_{N}^{\alpha }\left(t\right)=\frac{1}{N^{\alpha }}\sum_{i=1}^{N^{\alpha }}s_{i}^{\alpha }\left(t\right). \end{array}$$ (1)

Here, $s_{i}^{\alpha }\left(t\right)=\sum_{k}\delta \left(t-t_{i,k}^{\alpha }\right)$ with the Dirac *δ*-function denotes the spike train of an individual neuron *i* in population *α* with spike times $t_{i,k}^{\alpha }$. Empirically, the population activity is measured with a finite temporal resolution Δ*t*. In this case, we define the coarse-grained population activity as

$$\begin{array}{c} A_{N}^{\alpha }\left(t\right)=\frac{\Delta n^{\alpha }\left(t\right)}{N^{\alpha }\Delta t}, \end{array}$$ (2)

where Δ*n*^*α*^(*t*) is the number of neurons in population *α* that have fired in a time bin of size Δ*t* starting at time *t*. The two definitions converge in the limit Δ*t* → 0.

An example of population activities derived from spiking activity in a cortical circuit model under a step current stimulation is shown in Fig 1B. To bridge the scales between neurons and populations, the corresponding mean-field model should ideally result in the same population activities as obtained from the full microscopic model (Fig 1D). Because of the stochastic nature of the population activities, however, the qualifier “same” has to be interpreted in a statistical sense. The random fluctuations apparent in Fig 1B and 1D are a consequence of the finite number of neurons because microscopic stochasticity is not averaged out in the finite sum in Eq (1). This observation is important because estimated neuron numbers reported in experiments on local cortical circuits are relatively small [3, 73]. Therefore, a quantitatively valid population model needs to account for finite-size fluctuations. As mentioned above, we will refer to the population-level with finite size populations (*N* ∼ 50 to 2000 per population) as the *mesoscopic* level. In summary, we face the following question: is it possible to derive a closed set of evolution equations for the mesoscopic variables $A_{N}^{\alpha }\left(t\right)$ that follow the same statistics as the original microscopic model?

To address this question, we describe neurons by generalized integrate-and-fire (GIF) neuron models (Fig 1A (inset) and Methods, Sec. “Generalized integrate-and-fire model”) with escape noise [14]. In particular, neuron *i* of population *α* is modeled by a leaky integrate-and-fire model with dynamic threshold [49, 80]. The variables of this model are the membrane potential $u_{i}^{\alpha }\left(t\right)$ and the dynamic threshold $\vartheta_{i}^{\alpha }\left(t\right)=u_{\text{th}}+\int_{-\infty }^{t}\theta^{\alpha }\left(t-t^{\prime }\right)s_{i}^{\alpha }\left(t^{\prime }\right)\;dt^{\prime }$ (Fig 1A, inset), where *u*_th_ is a baseline threshold and *θ*^*α*^(*t*) is a spike-triggered adaptation kernel or filter function that accounts for adaptation [26, 47, 81–84] and other spike-history effects [14, 84] via a convolution with the neurons spike train $s_{i}^{\alpha }\left(t\right)$. In other words, the dynamic threshold depends on earlier spikes $t_{i,k}^{\alpha }$ of neuron *i*: $\vartheta_{i}^{\alpha }\left(t\right)\equiv \vartheta^{\alpha }\left(t,t_{i,k}^{\alpha }<t\right)$. Spikes are elicited stochastically depending on the present state of the neuron (Fig 1A, inset). Specifically, the probability that neuron *i* fires a spike in the next time step [*t*, *t* + Δ*t*] is given by λ_*i*_(*t*)Δ*t*, where $\lambda_{i}^{\alpha }\left(t\right)$ is the conditional intensity of neuron *i* (also called conditional rate or hazard rate):

$$\begin{array}{c} \lambda_{i}^{\alpha }\left(t\right)=f^{\alpha }\left(u_{i}^{\alpha }\left(t\right)-\vartheta^{\alpha }\left(t,t_{i,k}^{\alpha }<t\right)\right) \end{array}$$ (3)

with an exponential function $f^{\alpha }\left(x\right)=c^{\alpha }\exp\left(x/\Delta_{u}^{\alpha }\right)$. Analysis of experimental data has shown that the “softness” parameter $\Delta_{u}^{\alpha }$ of the threshold is in the range of 1 to 5 mV [85]. The parameter *c*^*α*^ can be interpreted as the instantaneous rate at threshold.

Besides the effect of a spike on the threshold as mediated by the filter function *θ*^*α*^(*t*), a spike also triggers a change of the membrane potential. In the GIF model (Methods, Sec. “Generalized integrate-and-fire model”), the membrane potential $u_{i}^{\alpha }\left(t\right)$ is reset after spiking to a reset potential *u*_r_, to which $u_{i}^{\alpha }\left(t\right)$ is clamped for an absolute refractory period *t*_ref_. Absolute refractoriness is followed by a period of relative refractoriness, where the conditional intensity Eq (3) is reduced. This period is determined by the relaxation of the membrane potential from the reset potential to the unperturbed or “free” potential, denoted *h*(*t*), which corresponds to the membrane potential dynamics in the absence of resets.

The GIF model accurately predicts spikes of cortical neurons under noisy current stimulation mimicking in-vivo like input [25, 26] and its parameters can be efficiently extracted from single neuron recordings [26, 27]. Variants of this model have also been suggested that explicitly incorporate biophysical properties such as fast sodium inactivation [86, 87], conductance-based currents [88] and synaptically-filtered background noise [89].

#### Mean-field approximations

In order to derive a mesoscopic mean-field theory for populations of GIF neurons, we first approximate the conditional intensity $\lambda_{i}^{\alpha }\left(t\right)$ of an individual neuron by an effective rate $\lambda_{A}^{\alpha }\left(t|{\hat{t}}_{i}^{\alpha }\right)$ that only depends on its last spike time ${\hat{t}}_{i}^{\alpha }$ and on the history of the population activity $A_{N}^{\alpha }\left(t^{\prime }\right)$, *t*′ < *t* (as expressed by the subscript “*A*”). This is called the quasi-renewal approximation [63]. Taking into account the dependence on the last spike is particularly important because of neuronal refractoriness.

To obtain such a quasi-renewal description we make two approximations. Firstly, we approximate the random connectivity by an effective full connectivity with proportionally scaled down synaptic weights (“mean-field approximation”). As a result, all neurons of the same population are driven by identical synaptic input (see Methods). This implies that for all neurons that had the same last spike time, the time course of the membrane potential is identical, $u_{i}^{\alpha }\left(t\right)\approx u_{A}\left(t,{\hat{t}}_{i}^{\alpha }\right)$. Secondly, we make the quasi-renewal approximation for GIF neurons [63], which replaces the threshold *ϑ*_*i*_(*t*) by an effective threshold $\vartheta_{A}^{\alpha }\left(t,{\hat{t}}_{i}^{\alpha }\right)$. Again, the effective threshold only depends on the last spike time and the history of the population activity. As a final result we obtain the conditional intensity for all neurons with a given last spike time $\hat{t}$ as

$$\begin{array}{c} \lambda_{A}^{\alpha }\left(t|\hat{t}\right)=f^{\alpha }\left(u_{A}^{\alpha }\left(t,\hat{t}\right)-\vartheta_{A}^{\alpha }\left(t,\hat{t}\right)\right) \end{array}$$ (4)

(Fig 1C, inset). A comparison of Eq (4) with Eq (3) shows that the explicit dependence on *all* past spike times $t_{i,k}^{\alpha }<\hat{t}$ of a given neuron *i* has disappeared. Instead, the conditional intensity now only depends on the *last* firing time $\hat{t}$ and the past population activity $A_{N}^{\alpha }\left(t^{\prime }\right)$, *t*′ < *t*. To keep the notation simple, we omit in the following the population label *α* at all quantities.

#### Finite-size mean field theory

**Fig 2:**
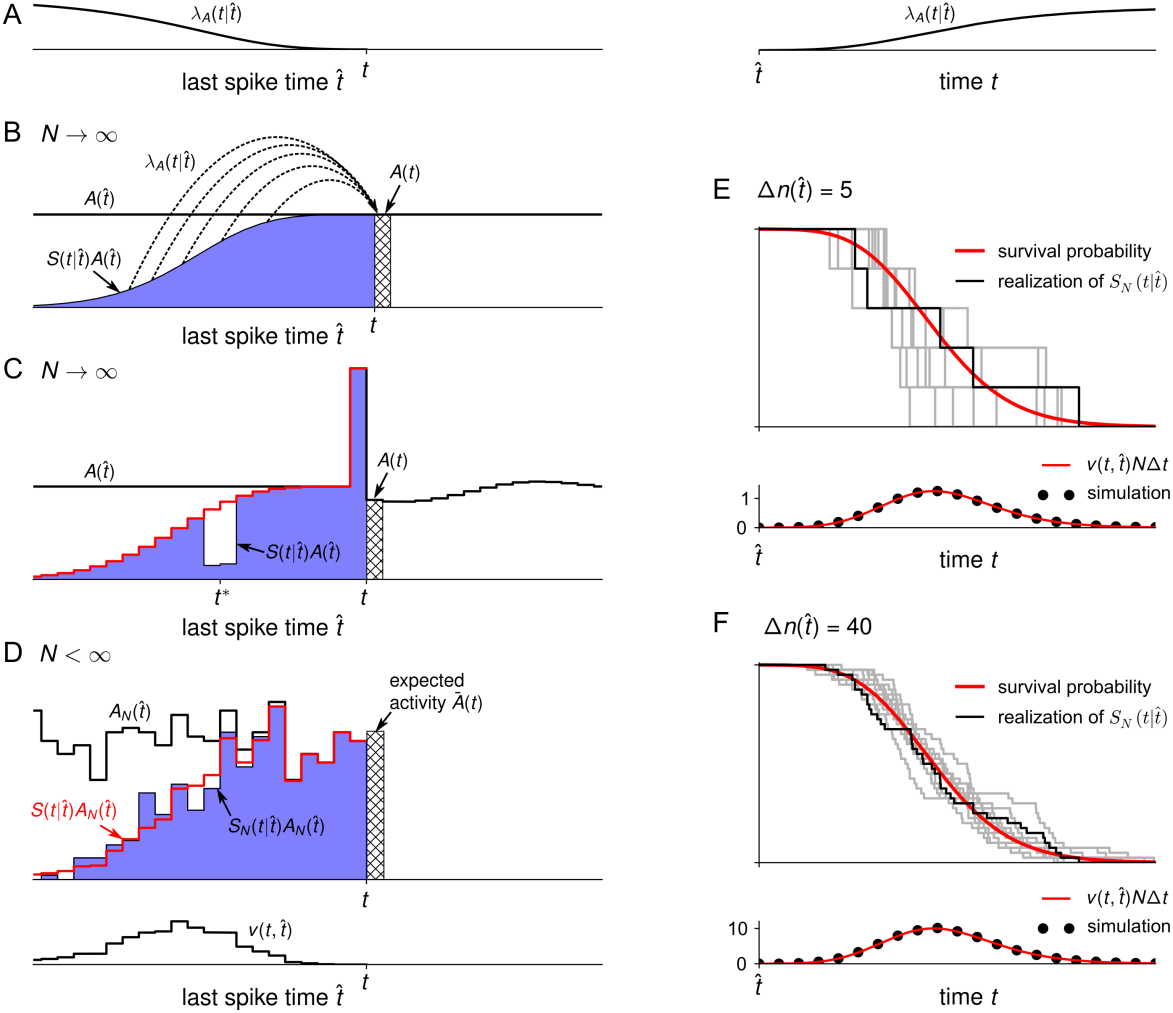
How fluctuations of the refractory density effect the population activity. (A) The conditional intensity $\lambda_{A}\left(t|\hat{t}\right)$ shown as a function of $\hat{t}$ (left) and *t* (right). Typically, the conditional intensity increases as the time since the last spike grows and the neuron recovers from refractoriness. (B) For *N* → ∞, the population activity *A*(*t*) (hatched bin) results from $\lambda_{A}\left(t|\hat{t}\right)$ averaged over the last spike times $\hat{t}$ with a weighting factor $S\left(t|\hat{t}\right)A\left(\hat{t}\right)$ (blue) corresponding to the density of last spike times. Here, $S(t|\hat{t})$ is the survival probability. (C) The last spike times $\hat{t}$ are discretized into time bins. In the bin immediately before *t*, a large fluctuation (blue peak) was induced by forcing some of the neurons with last spike time around t* to fire. At time *t*, the density of last spike times (blue) has a hole and a peak of equal probability mass. The red line shows the pseudo-density $S_{0}\left(t|\hat{t}\right)A\left(\hat{t}\right)$ that would be obtained if we had used the survival probability $S_{0}\left(t|\hat{t}\right)$ of the unperturbed system. The peak at $\hat{t}=t-\Delta t$ does not contribute to the activity *A*(*t*) because of refractoriness, but the hole at $\hat{t}=t^{*}$ contributes with a non-vanishing rate (A), implying a reduction of *A*(*t*) (hatched bin). (D) For a finite population size (here *N* = 400), the refractory density $S_{N}\left(t|\hat{t}\right)A_{N}\left(\hat{t}\right)$ (blue), determines the expectation $\bar{A}\left(t\right)$ (hatched bin) of the fluctuating activity *A*_*N*_(*t*). Analogous to the forced fluctuation in (C), the finite-size fluctuations are associated with negative and positive deviations in the refractory density (holes and overshoots) compared to the non-normalized density $S\left(t|\hat{t}\right)A_{N}\left(\hat{t}\right)$ (red line) that would be obtained if only the mean $S(t|\hat{t})$ and not the exact survival fraction $S_{N}\left(t|\hat{t}\right)$ had been taken into account. The variance of the deviations is proportional to $v(t,\hat{t})$ given by Eq (12). As a function of $\hat{t}$, $v(t,\hat{t})$ shows the range of $\hat{t}$ where deviations are most prominent (bottom). (E, F) Given the number of neurons firing in the bin $\left[\hat{t},\hat{t}+\Delta t\right)$, $\Delta n(\hat{t})$, the fraction of neurons that survive until time *t* is shown for ten realizations (gray lines, one highlighted in black for clarity). The mean fraction equals the survival probability $S(t|\hat{t})$ (red line, top panel). The variance of the number of survived neurons at time *t*, $v(t,\hat{t})N\Delta t$, is shown at the bottom (red line: semi-analytic theory, Eq (12); circles: simulation). (E) $\Delta n(\hat{t})=5$, (F) $\Delta n(\hat{t})=40$.

In this section, we present the main theoretical results with a focus on the finite-size effects arising from neuronal refractoriness. So far, we have effectively reduced the firing probability of a neuron to a function $\lambda_{A}\left(t|\hat{t}\right)$ that only depends on its last spike time $\hat{t}$ (Fig 2A). This allows us to describe the evolution of the system by the density of the last spike time [23, 68, 88, 89]. Because the last spike time characterizes the refractory state of the neuron, this density will also be referred to as the refractory density. Before we describe the novel finite-*N* theory, it is instructive to first recall the population equations for the case of infinite *N* (Fig 2B and 2C). Let us look at the population of neurons at time *t* and ask the following question: What fraction of these neurons has fired their last spike between $\hat{t}$ and $\hat{t}+d\hat{t}$? This fraction is given by the number of neurons $A\left(\hat{t}\right)d\hat{t}$ that have fired in this interval multiplied by the survival probability $S(t|\hat{t})$ that such a neuron has not fired again until time *t*. In other words, the product $Q_{\infty }\left(t,\hat{t}\right)=S\left(t|\hat{t}\right)A\left(\hat{t}\right)$ evaluated at time *t* represents the density of last spike times $\hat{t}$. Because a neuron with last spike time $\hat{t}$ emits a spike with rate $\lambda_{A}\left(t|\hat{t}\right)$, the total population activity at time *t* is given by the integral [23]

$$\begin{array}{c} A\left(t\right)=\int_{-\infty }^{t}\lambda_{A}\left(t|\hat{t}\right)S\left(t|\hat{t}\right)A\left(\hat{t}\right)\;d\hat{t}. \end{array}$$ (5)

This situation is depicted in Fig 2B. At the same time, the survival probability $S(t|\hat{t})$ of neurons that fired their last spike at $\hat{t}$ decays according to

$$\begin{array}{c} \frac{\partial S(t|\hat{t})}{\partial t}=-\lambda_{A}\left(t|\hat{t}\right)S\left(t|\hat{t}\right) \end{array}$$ (6)

with initial condition $S(\hat{t}|\hat{t})=1$ (Fig 2E and 2F red line). Eqs (5) and (6) define the population dynamics for *N* → ∞ [16, 23, 89].

In the limit *N* → ∞, the dynamics of *A*_*N*_(*t*) = *A*(*t*) is deterministic because microscopic noise averages out. Nevertheless, the infinite-*N* case is useful to understand the main effect of fluctuations in the finite-*N* case. To this end, let us perform the following thought experiment: in a small time interval of length Δ*t* immediately before time *t*, we induce a large, positive fluctuation in the activity by forcing many of the neurons with last spike close to a given time $\hat{t}=t^{*}$ to fire a spike (Fig 2C). As a result, the density of last spike times at time *t* exhibits a large peak just prior to time *t* corresponding to the large number of neurons that have been forced to fire in the time interval [*t* − Δ*t*, *t*). At the same time, these neurons leave behind a “hole” in the density around $\hat{t}=t^{*}$. Because the number of neurons is conserved, this hole exactly balances the area under the peak, and hence, the density of last spike times remains normalized. However, the two fluctuations (the hole and the peak) have two different effects on the population activity after time *t*. Specifically, the hole implies that some of the neurons which normally would have fired at time *t* with a nonzero rate λ_*A*_(*t*|t*) > 0 are no longer available. Moreover, neural refractoriness implies that neurons which fired in the peak have a small or even vanishing rate λ_*A*_(*t*|*t* − Δ*t*) ≈ 0 at time *t*. As a result, the population activity is reduced shortly after the perturbation (Fig 2C). This example highlights the importance of the normalization of the refractory density as well as the state-dependence of the firing probability for understanding the effect of fluctuations. In particular, the normalization condition and neuronal refractoriness imply that a positive fluctuation of the population activity is succeeded by a negative fluctuation, and vice versa. This behavior is characteristic for spiking neurons, which are known to exhibit negative auto-correlations of their mean-centered spike trains at short time lags (see e.g. [14, 53, 90]).

We now turn to the finite-size case. In this case, it is advantageous to discretize the last spike times into small bins of size Δ*t* that begin at the grid points ${\hat{t}}_{k}=k\Delta t$, k∈Z. Furthermore, we adopt the definition of the coarse-grained population activity, Eq (2), i.e. we consider the number of spikes $\Delta n({\hat{t}}_{k})$ in the time bin $\left[{\hat{t}}_{k},{\hat{t}}_{k}+\Delta t\right)$. Instead of the survival probability, we introduce the fraction of survived neurons $S_{N}\left(t|{\hat{t}}_{k}\right)$, $t>{\hat{t}}_{k}$, such that $S_{N}\left(t|{\hat{t}}_{k}\right)\Delta n\left({\hat{t}}_{k}\right)$ is the number of neurons from bin *k* that have not fired again until time *t* (Fig 2D and 2E). Dividing this number by *N*Δ*t* and taking the continuum limit Δ*t* → 0, yields the density of last spike times $Q_{N}\left(t,\hat{t}\right)=S_{N}\left(t|\hat{t}\right)A_{N}\left(\hat{t}\right)$ in the case of finite *N*. Since all neurons are uniquely identified by their last spike time, this density is normalized [23]

$$\begin{array}{c} 1=\int_{-\infty }^{t}S_{N}\left(t|\hat{t}\right)A_{N}\left(\hat{t}\right)\;d\hat{t}. \end{array}$$ (7)

We note that differentiating this equation with respect to time *t* yields the population activity $A_{N}\left(t\right)=-\int_{-\infty }^{t}\partial_{t}S_{N}\left(t|\hat{t}\right)A_{N}\left(\hat{t}\right)\;d\hat{t}$ as the formal finite-size analog of Eq (5). The change of the survival fraction $\partial_{t}S_{N}\left(t|\hat{t}\right)$, however, is not deterministic anymore as in Eq (6) but follows a stochastic jump process (Fig 2E and 2F): In the time step [*t*, *t* + Δ*t*), the number of survived neurons for a given bin ${\hat{t}}_{k}$ in the past, $S_{N}\left(t|{\hat{t}}_{k}\right)\Delta n\left({\hat{t}}_{k}\right)$, makes a downstep $X(t,{\hat{t}}_{k})$ that corresponds to the number of neurons that fire in the group with last spike time ${\hat{t}}_{k}$. For sufficiently small Δ*t*, this number is Poisson-distributed with mean $\lambda_{A}\left(t|{\hat{t}}_{k}\right)S_{N}\left(t|{\hat{t}}_{k}\right)\Delta n\left({\hat{t}}_{k}\right)\Delta t$. Hence, the fraction of survived neurons $S_{N}\left(t|{\hat{t}}_{k}\right)$ evolves in time like a random stair case according to the update rule $S_{N}\left(t+\Delta t|{\hat{t}}_{k}\right)=S_{N}\left(t|{\hat{t}}_{k}\right)-X\left(t,{\hat{t}}_{k}\right)/\Delta n\left({\hat{t}}_{k}\right)$. The activity *A*_*N*_(*t*) = Δ*n*(*t*)/(*N*Δ*t*) in the time bin starting at *t* is given by the sum of all the downsteps, $\Delta n\left(t\right)=\sum_{{\hat{t}}_{k}<t}\;X\left(t,{\hat{t}}_{k}\right)$, where the sum runs over all possible last spike times. This updating procedure represents the evolution of the density of last spike times, $Q_{N}\left(t,\hat{t}\right)=S_{N}\left(t|\hat{t}\right)A_{N}\left(\hat{t}\right)$, for finite *N* under the quasi-renewal approximation (cf. Methods, Eqs (41) and (42)). Although it is possible to simulate such a finite-*N* stochastic process using the downsteps $X(t,{\hat{t}}_{k})$, this process will not yield the reduced mesoscopic dynamics that we are looking for. The reason is that the variable $S_{N}\left(t|{\hat{t}}_{k}\right)$ refers to the subpopulation of neurons that fired in the small time bin at ${\hat{t}}_{k}$. For small Δ*t*, the size of the subpopulation, $\Delta n({\hat{t}}_{k})$, is a *small* number, much smaller than *N*. In particular, in the limit Δ*t* → 0, the simulation of $S_{N}\left(t|{\hat{t}}_{k}\right)\Delta n\left({\hat{t}}_{k}\right)$ for all ${\hat{t}}_{k}$ in the past would be as complex as the original microscopic dynamics of *N* neurons. Therefore we must consider such a simulation as a microscopic simulation. To see the difference to a mescoscopic simulation, we note that the population activity *A*_*N*_(*t*) involves the summation of many random processes (the downsteps $X(t,{\hat{t}}_{k})$) over many small bins. If we succeed to simulate directly *A*_*N*_(*t*) from an underlying rate dynamics that depends deterministically on the past activities *A*_*N*_(*t*′), *t*′ < *t*, we will have a truely mescoscopic simulation. How to arrive at a formulation directly at the level of mescocopic quantities will be the topic of the rest of this section.

The crucial question is whether the stochasticity of the many different random processes ${\left\{S_{N}\left(t|{\hat{t}}_{k}\right)\right\}}_{{\hat{t}}_{k}<t}$ can be reduced to a single effective noise process that drives the dynamics on the mesoscopic level. To this end, we note that for small Δ*t* and given history $\Delta n({\hat{t}}_{k})$, ${\hat{t}}_{k}<t$, each bin ${\hat{t}}_{k}$ contributes with rate $\lambda_{A}\left(t|{\hat{t}}_{k}\right)S_{N}\left(t|{\hat{t}}_{k}\right)\Delta n\left({\hat{t}}_{k}\right)$ a Poisson random number of spikes to the total activity at time *t* (Fig 2D). Therefore, the total number of spikes Δ*n*(*t*) is Poisson distributed with mean $N\bar{A}\left(t\right)\Delta t$, where $\bar{A}\left(t\right)$ is the *expected* population rate

$$\begin{array}{c} \bar{A}\left(t\right)=\int_{-\infty }^{t}\lambda_{A}\left(t|\hat{t}\right)S_{N}\left(t|\hat{t}\right)A_{N}\left(\hat{t}\right)\;d\hat{t}. \end{array}$$ (8)

Here, the integral extends over all last spike times $\hat{t}$ up to but not including time *t*. Eq (8) still depends on the stochastic variables ${\left\{S_{N}\left(t|{\hat{t}}_{k}\right)\right\}}_{{\hat{t}}_{k}<t}$. The main strategy to remove this microscopic stochasticity is to use the evolution of the survival probability $S(t|\hat{t})$, given by Eq (6), and the normalization condition Eq (7). For finite *N*, the quantity $S(t|{\hat{t}}_{k})$ is formally defined as the solution of Eq (6) and can be interpreted as the mean of the survival fraction $S_{N}\left(t|{\hat{t}}_{k}\right)$ (Fig 2E and 2F, see Methods). Importantly, $S(t|{\hat{t}}_{k})$ is a valid mesoscopic quantity since it only depends on the mesoscopic population activity *A*_*N*_ (through $\lambda_{A}\left(t|{\hat{t}}_{k}\right)$, cf. Eq (6)), and not on a specific microscopic realization. Combining the survival probability $S(t|\hat{t})$ with the actual history of the mesoscopic activity $A_{N}\left(\hat{t}\right)$ for $\hat{t}<t$ yields the pseudo-density $Q\left(t,{\hat{t}}_{k}\right)=S\left(t|{\hat{t}}_{k}\right)A_{N}\left({\hat{t}}_{k}\right)$. In contrast to the macroscopic density $Q_{\infty }\left(t,\hat{t}\right)=S\left(t|\hat{t}\right)A\left(\hat{t}\right)$ in Eq (5) or the microscopic density $Q_{N}\left(t,{\hat{t}}_{k}\right)=S_{N}\left(t|{\hat{t}}_{k}\right)A_{N}\left({\hat{t}}_{k}\right)$, the pseudo-density is not normalized. However, the pseudo-density $S\left(t|{\hat{t}}_{k}\right)A_{N}\left({\hat{t}}_{k}\right)$ has the advantage that it is based on mesoscopic quantities only.

Let us split the survival fraction into the mesoscopic quantity $S(t|{\hat{t}}_{k})$ and a microscopic deviation, $S_{N}\left(t|{\hat{t}}_{k}\right)=S\left(t|{\hat{t}}_{k}\right)+\delta S\left(t|{\hat{t}}_{k}\right)$. In analogy to the artificial fluctuation in our thought experiment, endogenously generated fluctuations in the finite-size system are accompanied by deviations of the microscopic density $S_{N}\left(t|{\hat{t}}_{k}\right)A_{N}\left({\hat{t}}_{k}\right)$ from the pseudo-density $S\left(t|{\hat{t}}_{k}\right)A_{N}\left({\hat{t}}_{k}\right)$ (Fig 2C and 2D, red line). A negative deviation ($\delta S(t|{\hat{t}}_{k})<0$) can be interpreted as a hole and a positive deviation ($\delta S(t|{\hat{t}}_{k})>0$) as an overshoot (compare red curve and blue histogram in Fig 2D). Similar to the thought experiment, the effect of these deviations needs to be weighted by the conditional intensity $\lambda_{A}\left(t|{\hat{t}}_{k}\right)$ in order to arrive at the population activity. Eq (8) yields

$$\begin{array}{c} \bar{A}\left(t\right)=\int_{-\infty }^{t}\lambda_{A}\left(t|\hat{t}\right)S\left(t|\hat{t}\right)A_{N}\left(\hat{t}\right)\;d\hat{t}+\int_{-\infty }^{t}\lambda_{A}\left(t|\hat{t}\right)\delta S\left(t|\hat{t}\right)A_{N}\left(\hat{t}\right)\;d\hat{t}. \end{array}$$ (9)

Analogously, the normalization of the refractory density, Eq (7), can be written as

$$\begin{array}{c} 1=\int_{-\infty }^{t}S\left(t|\hat{t}\right)A_{N}\left(\hat{t}\right)\;d\hat{t}+\int_{-\infty }^{t}\delta S\left(t|\hat{t}\right)A_{N}\left(\hat{t}\right)\;d\hat{t}. \end{array}$$ (10)

We refer to the second integral in Eq (9) as a correction term because it corrects for the error that one would make if one sets *S*_*N*_ = *S* in Eq (8). This correction term represents the overall contribution of the holes (*δS* < 0) and overshoots (*δS* > 0) to the expected activity.

To eliminate the microscopic deviations $\delta S(t|\hat{t})$ in Eq (9) we use the normalization condition, Eq (10). This is possible because the correction term is tightly constrained by the sum of all holes and overshoots, $\int_{-\infty }^{t}\delta S\left(t|\hat{t}\right)A_{N}\left(\hat{t}\right)\;d\hat{t}$, which by Eq (10), is completely determined by the past mesoscopic activities. Eqs (9) and (10) suggest to make the deterministic ansatz $\int_{-\infty }^{t}\lambda_{A}\delta S\left(t|\hat{t}\right)A_{N}\left(\hat{t}\right)\;d\hat{t}\approx \Lambda \left(t\right)\int_{-\infty }^{t}\delta S\left(t|\hat{t}\right)A_{N}\left(\hat{t}\right)\;d\hat{t}$ for the correction term. As shown in Methods (“Mesoscopic population equations”), the optimal rate Λ(*t*) that minimizes the mean squared error of this approximation is given by

$$\begin{array}{c} \Lambda \left(t\right)=\frac{\int_{-\infty }^{t}\lambda_{A}\left(t|\hat{t}\right)v\left(t,\hat{t}\right)\;d\hat{t}}{\int_{-\infty }^{t}v\left(t,\hat{t}\right)\;d\hat{t}}. \end{array}$$ (11)

Here, the quantity $v(t,\hat{t})$, called variance function, obeys the differential equation

$$\begin{array}{c} \frac{\partial v}{\partial t}=-2\lambda_{A}\left(t|\hat{t}\right)v+\lambda_{A}\left(t|\hat{t}\right)S\left(t|\hat{t}\right)A_{N}\left(\hat{t}\right) \end{array}$$ (12)

with initial condition $v(\hat{t},\hat{t})=0$ (see Methods, Eq (51). Importantly, the dynamics of *v* involves mesoscopic quantities only, and hence *v* is mesoscopic. As shown in Methods and illustrated in Fig 2D (bottom), we can interpret $v(t,{\hat{t}}_{k})N\Delta t$ as the variance of the number of survived neurons, $S_{N}\left(t|{\hat{t}}_{k}\right)\Delta n\left({\hat{t}}_{k}\right)$. To provide an interpretation of the effective rate Λ(*t*) we note that, for fixed *t*, the normalized variance $v\left(t,\hat{t}\right)/\int_{-\infty }^{t}v\left(t,\hat{t}\right)\;d\hat{t}$ is a probability density over $\hat{t}$. Thus, the effective rate Λ(*t*) can be regarded as a weighted average of the conditional intensity $\lambda_{A}\left(t|\hat{t}\right)$ that accounts for the expected amplitude of the holes and overshoots.

Using the effective rate Λ(*t*) in Eq (9) results in the expected activity

$$\begin{array}{c} \bar{A}\left(t\right)=\int_{-\infty }^{t}\lambda_{A}\left(t|\hat{t}\right)S\left(t|\hat{t}\right)A_{N}\left(\hat{t}\right)\;d\hat{t}+\Lambda \left(t\right)\left(1-\int_{-\infty }^{t}S\left(t|\hat{t}\right)A_{N}\left(\hat{t}\right)\;d\hat{t}\right). \end{array}$$ (13)

Looking at the structure of Eq (13), we find that the first term is the familiar population integral known from the infinite-*N* case, Eq (5). The second term is a correction that is only present in the finite-*N* case. In fact, in the limit *N* → ∞, the pseudo-density $S\left(t|\hat{t}\right)A_{N}\left(\hat{t}\right)$ converges to the macroscopic density $S\left(t|\hat{t}\right)A\left(\hat{t}\right)$, which is normalized to unity. Hence the correction term vanishes and we recover the population Eq (5) for the infinite system.

To obtain the population activity we consider an infinitesimally small time scale *dt* such that the probability of a neuron to fire during an interval [*t*, *t* + *dt*) is much smaller than one, i.e. $\bar{A}\left(t\right)dt\ll 1$. On this time scale, the total number of spikes *dn*(*t*) is an independent, Poisson distributed random number with mean $\bar{A}\left(t\right)Ndt$, where $\bar{A}\left(t\right)$ is given by Eq (13). From Eq (2) thus follows the population activity

$$\begin{array}{c} A_{N}\left(t\right)=\frac{1}{N}\frac{dn(t)}{dt},\;dn\left(t\right)∼\text{Pois}\left[\bar{A}\left(t\right)Ndt\right]. \end{array}$$ (14)

Alternatively, the population activity can be represented as a *δ*-spike train, or “shot noise”, $A_{N}\left(t\right)=\frac{1}{N}\sum_{k}\delta \left(t-t_{\text{pop},k}\right)$, where ${\left\{t_{\text{pop},k}\right\}}_{k\in Z}$ is a random point process with a conditional intensity function $\lambda_{\text{pop}}(t|H_{t})=N\bar{A}\left(t\right)$. Here, the condition $H_{t}$ denotes the history of the point process {*t*_pop,*k*_} up to (but not including) time *t*, or equivalently the history of the population activity $A_{N}\left(\hat{t}\right)$ for $\hat{t}<t$. The conditional intensity means that the conditional expectation of the population activity is given by $\langle A_{N}\left(t\right)\rangle {|}_{H_{t}}=\bar{A}\left(t\right)$, which according to Eq (13) is indeed a deterministic functional of the past activities. Finally, we note that the case of large but finite populations permits a Gaussian approximation, which yields the more explicit form

$$\begin{array}{c} A_{N}\left(t\right)=\bar{A}\left(t\right)+\sqrt{\frac{\bar{A}\left(t\right)}{N}}\xi \left(t\right). \end{array}$$ (15)

Here, *ξ*(*t*) is a Gaussian white noise with correlation function 〈*ξ*(*t*)*ξ*(*t*′)〉 = *δ*(*t* − *t*′). The correlations of *ξ*(*t*) are white because spikes at *t*′ and *t* > *t*′ are independent *given* the expected population activity $\bar{A}\left(t\right)$ at time *t*. However, we emphasize that the expected population activity $\bar{A}\left(t\right)$ does include information on the past fluctuations *ξ*(*t*′) at time *t*′. Therefore the fluctuations of the total activity *A*_*N*_(*t*) are not white but a sum of a colored process $\bar{A}\left(t\right)$ and a white-noise process *ξ*(*t*) [55]. The white noise gives rise to the delta peak of the auto-correlation function at zero time lag which is a standard feature of any spike train, and hence also of *A*_*N*_(*t*). The colored process $\bar{A}\left(t\right)$, on the other hand, arises from Eq (13) via a filtering of the *actual* population activity $A_{N}\left(\hat{t}\right)$ which includes the past fluctuations $\xi (\hat{t})$. For neurons with refractoriness, $\bar{A}\left(t\right)$ is negatively correlated with recent fluctuations $\xi (\hat{t})$ (cf. the thought experiment of Fig 2B) leading to a trough at small time lags in the spike auto-correlation function [14, 53, 90].

The set of coupled Eqs (6), (12), (11)–(14) constitute the desired mesoscopic population dynamics and is the main result of the paper. The dynamics is fully determined by the history of the mesoscopic population activity *A*_*N*_. The Gaussian white noise in Eq (15) or the independent random number involved in the generation of the population activity via Eq (14) is the only source of stochasticity and summarizes the effect of microscopic noise on the mesoscopic level. Microscopic detail such as the knowledge of how many neurons occupy a certain microstate $\hat{t}$ has been removed.

One may wonder where the neuronal interactions enter in the population equation. Synaptic interactions are contained in the conditional intensity $\lambda_{A}\left(t|\hat{t}\right)$ which depends on the membrane potential $u_{A}\left(t,\hat{t}\right)$, which in turn is driven by the synaptic current that depends on the population activity via Eq (29) in Methods. An illustration of the derived mesoscopic model is shown in Fig 1C (inset). In this section, we considered a single population to keep the notation simple. However, it is straightforward to formulate the corresponding equations for the case of several equations as shown in Methods, Sec. “Several populations”.

#### Stochastic population dynamics can be efficiently simulated forward in time

The stochastic population equations provide a rapid means to integrate the population dynamics on a mesoscopic level. To this end, we devised an efficient integration algorithm based on approximating the infinite integrals in the population equation Eq (13) by discrete sums over a finite number of refractory states $\hat{t}$ (Methods, Sec. “Numerical implementation”). The algorithm involves the generation of only one random number per time step and population, because the activity is sampled from the mesoscopic rate ${\bar{A}}^{\alpha }\left(t\right)$. In contrast, the microscopic simulation requires in each time step to draw a random number for each neuron. Furthermore, because the population equations do not depend on the number of neurons, we expect a significant speed-up factor for large neural networks compared to a corresponding microscopic simulation. For example, the microscopic simulation of the cortical column in Fig 1B took 13.5 minutes to simulate 10 seconds of biological time, whereas the corresponding forward integration of the stochastic population dynamics (Fig 1D) took only 6.6 seconds on the same machine (see Sec. “Comparison of microscopic and mesoscopic simulations”).

A pseudocode of the algorithm to simulate neural population dynamics is provided in Methods (Sec. “Numerical implementation”). In addition to that, a reference implementation of this algorithm is publicly available under https://github.com/schwalger/mesopopdyn_gif, and will be integrated in the Neural Simulation Tool (NEST) [91], https://github.com/nest/nest-simulator, as a module presumably named gif_pop_psc_exp.

### Two different consequences of finite *N*

For a first analysis of the finite-size effects, we consider the special case of a fully-connected network of Poisson neurons with absolute refractory period [14]. In this case, the conditional intensity can be represented as $\lambda_{A}\left(t|\hat{t}\right)=f(h(t))\Theta \left(t-\hat{t}-t_{\text{ref}}\right)$, where *t*_ref_ is the absolute refractory period, Θ(⋅) is the Heaviside step function and *h*(*t*) is the free membrane potential, which obeys the passive membrane dynamics

$$\begin{array}{c} \tau_{\text{m}}\frac{dh}{dt}=-h+\mu \left(t\right)+\tau_{\text{m}}J\;\left(\epsilon *A_{N}\right)\left(t\right), \end{array}$$ (16)

where *τ*_m_ is the membrane time constant, *μ*(*t*) = *u*_rest_ + *RI*(*t*) (where *u*_rest_ is the resting potential and *R* is the membrane resistance) accounts for all currents *I*(*t*) that are independent of the population activities, *J* is the synaptic strength and *ϵ*(*t*) is a synaptic filter kernel (see Methods, Eq (27) for details). For the mathematical analysis, we assume that the activity *A*_*N*_(*t*) and input *μ*(*t*) have started at *t* = −∞ so that we do not need to worry about initial conditions. In a simulation, we could for example start at *t* = 0 with initial conditions *A*_*N*_(*t*) = *δ*(*t*) for *t* ≤ 0 and *h*(0) = 0.

For the conditional intensity given above, the effective rate Λ(*t*), Eq (11), is given by Λ(*t*) = *f*(*h*(*t*)) because the variance $v(t,\hat{t})$ is zero during the absolute refractory period $t-t_{\text{ref}}\leq \hat{t}<t$. As a result, the mesoscopic population Eq (13) reduces to the simple form

$$\begin{array}{c} \bar{A}\left(t\right)=f\left(h\left(t\right)\right)\left[1-\int_{t-t_{\text{ref}}}^{t}A_{N}\left(\hat{t}\right)\;d\hat{t}\right]. \end{array}$$ (17)

This mesoscopic equation is exact and could have been constructed directly in this simple case. For *N* → ∞, where *A*_*N*_(*t*) becomes identical to $\bar{A}\left(t\right)$, this equation has been derived by Wilson and Cowan [16], see also [14, 23, 92]. The intuitive interpretation of Eq (17) is that the activity at time *t* consists of two factors, the “free” rate λ_free_(*t*) = *f*(*h*(*t*)) that would be expected in the absence of refractoriness and the fraction of actually available (“free”) neurons that are not in the refractory period. For finite-size populations, these two factors explicitly reveal two distinct finite-size effects: firstly, the free rate is driven by the fluctuating population activity *A*_*N*_(*t*) via Eq (16) and hence the free rate exhibits finite-size fluctuations. This effect originates from the transmission of the fluctuations through the recurrent synaptic connectivity. Secondly, the fluctuations of the population activity impacts the refractory state of the population, i.e. the fraction of free neurons, as revealed by the second factor in Eq (17). In particular, a large positive fluctuations of *A*_*N*_ in the recent past reduces the fraction of free neurons, which causes a negative fluctuation of the number $N\bar{A}\left(t\right)\Delta t$ of expected firings in the next time step. Therefore, refractoriness generates negative correlations of the fluctuations 〈Δ*A*(*t*)Δ*A*(*t*′)〉 for small |*t* − *t*′|. We note that such a decrease of the expected rate would not have been possible if the correction term in Eq (13) was absent. However, incorporating the effect of recent fluctuations (i.e. fluctuations in the number of refractory neurons) on the number of free neurons by adding the correction term, and thereby balancing the total number of neurons, recovers the correct Eq (17).

The same arguments can be repeated in the general setting of Eq (13). Firstly, the conditional intensity $\lambda_{A}\left(t|\hat{t}\right)$ depends on the past fluctuations of the population activity because of network feedback. Secondly, the fluctuations lead to an imbalance in the number of neurons across different states of relative refractoriness (i.e. fluctuations do not add up to zero) which gives rise to the “correction term”, i.e. the second term on the r.h.s. of Eq (13).

### Comparison of microscopic and mesoscopic simulations

We wondered how well the statistics of the population activities obtained from the integration of the mesoscopic equations compare with the corresponding activities generated by a microscopic simulation. As we deal with a finite-size system, not only to the first-order statistics (mean activity) but also higher-order statistics needs to be considered. Because there are several approximations involved (e.g. full connectivity, quasi-renewal approximation and effective rate of fluctuations in the refractory density), we do not expect a perfect match. To compare first- and second-order statistics, we will mainly use the power spectrum of the population activities in the stationary state (see Methods, Sec. “Power spectrum”).

#### Mesoscopic equations capture refractoriness

**Fig 3:**
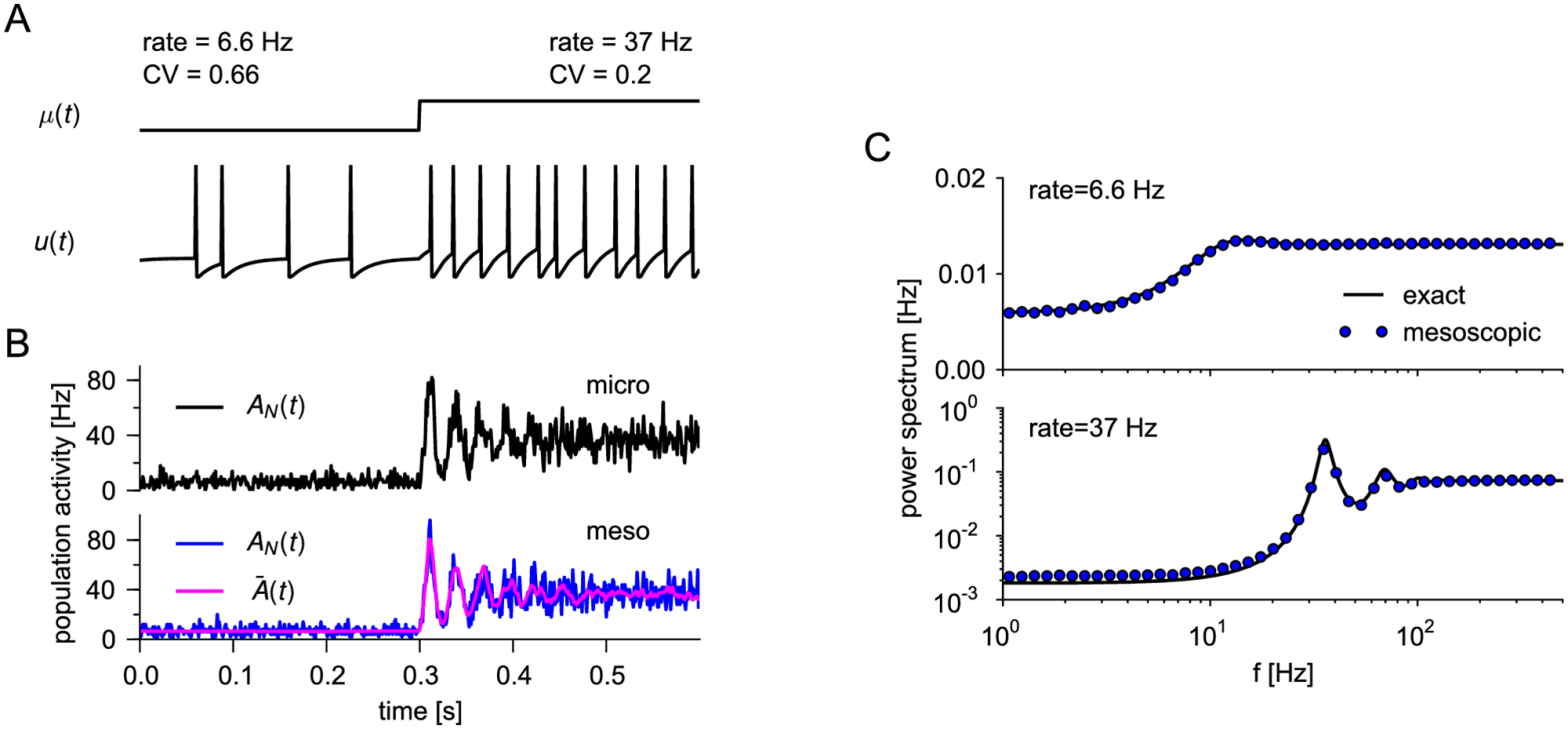
Population activity of uncoupled leaky integrate-and-fire neurons without adaptation. (A) Neurons were stimulated by a step current *I*_ext_(*t*) such that *μ* = *u*_rest_ + *RI*_ext_(*t*) increased from *μ* = 15 mV to *μ* = 30 mV (top). Voltage trace of one of 500 neurons (bottom). Stationary firing statistics (rate and coefficient of variation (CV) of the interspike intervals) corresponding to the two stimuli are indicated above the step current. (B) Realizations of the population activity *A*_*N*_(*t*) for the microscopic (top) and mesoscopic (bottom, blue line) simulation. The magenta line shows the expected population rate $\bar{A}\left(t\right)$ given the past actual realization *A*_*N*_(*t*′) for *t*′ < *t*. (C) The power spectrum of the stationary activity *A*_*N*_(*t*) obtained from renewal theory, Eq (134), (black solid line) and from the mesoscopic simulation (blue circles). The top and bottom panel corresponds to weak (*μ* = 15 mV) and strong (*μ* = 30 mV) constant stimulation (transient removed).

Our theory describes the interplay between finite-size fluctuations and spike-history effects. The most prominent spike-history effect is refractoriness, i.e. the strong effect of the last spike on the current probability to spike. To study this effect, we first focus on a population of uncoupled neurons with a constant threshold corresponding to leaky integrate-and-fire (LIF) models without adaptation (Fig 3). The reset of the membrane potential after each spike introduces a period of relative refractoriness, where spiking is less likely due to a hyper-polarized membrane potential (Fig 3A). Because of the reset to a fixed value, the spike trains of the LIF neurons are renewal processes. In the stationary state, the fluctuation statistics as characterized by the power spectrum is known analytically for the case of a population of independent renewal spike trains (Eq (134) in Methods).

A single realization of the population activity *A*_*N*_(*t*) fluctuates around the expected activity $\bar{A}\left(t\right)$ that exhibits a typical ringing in response to a step current stimulation [23, 93]. The time course of the expected activity as well as the size of fluctuations are roughly similar for microscopic simulation (Fig 3B, top) and the numerical integration of the population equations (Fig 3B, bottom). We also note that the expected activity $\bar{A}\left(t\right)$ is not constant in the stationary regime but shows weak fluctuations. This is because of the feedback of the actual realization of *A*_*N*_(*t*′) for *t*′ < *t* onto the dynamics of $\bar{A}\left(t\right)$, Eq (13).

A closer inspection confirms that the fluctuations generated by the mesoscopic population dynamics in the stationary state exhibit the same power spectrum (Fig 3C) as the theoretically predicted one, which is given by Eq (134). In particular, the mesoscopic equations capture the fluctuation statistics even at high firing rates, where the power spectrum strongly deviates from the white (flat) power spectrum of a Poisson process (Fig 3C bottom). The pronounced dip at low-frequencies is a well-known signature of neuronal refractoriness [94].

#### Mesoscopic equations capture adaptation and burstiness

**Fig 4:**
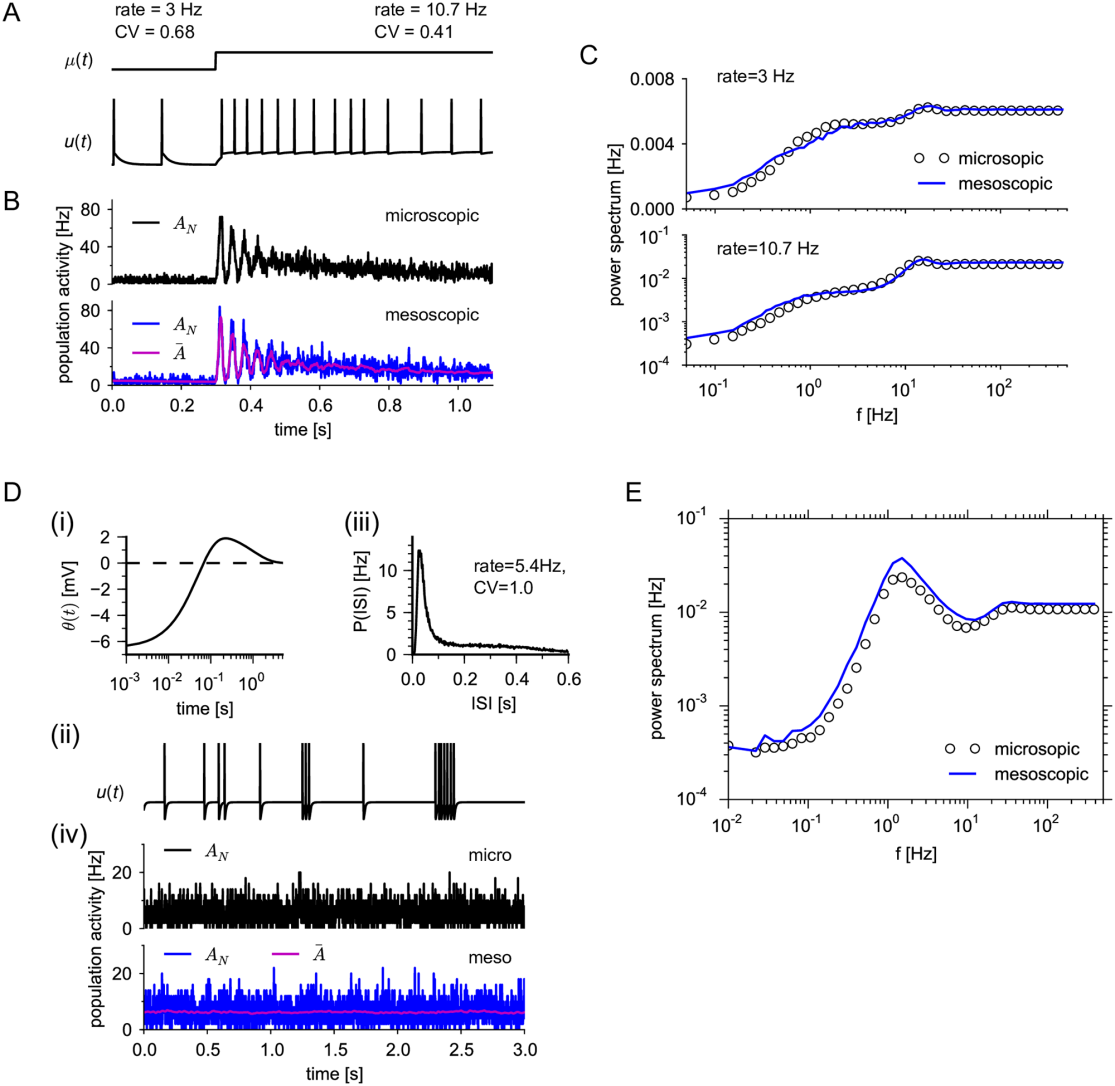
Population dynamics captures adaptation and burstiness. (A) 500 adapting leaky integrate-and-fire neurons were stimulated by a step current *I*_ext_ that increased *μ* = *u*_rest_ + *RI*_ext_(*t*) from *μ* = 12 mV to *μ* = 27 mV (top). Voltage trace of one neuron (bottom). Stationary firing statistics (rate and coefficient of variation (CV)) corresponding to the two stimuli are indicated above the step current. (B) Realizations of the population activity obtained from microscopic simulation (black) and mesoscopic population equation (blue) as well as $\bar{A}\left(t\right)$ (magenta). (C) Power spectra corresponding to the stationary activity (averaged over 1024 trials each of 20 s length) at low and high firing rates as in (A), circles and lines depict microscopic and mesoscopic case, respectively. Parameters in (A)–(C): *u*_th_ = 10 mV, *u*_r_ = 25 mV, threshold kernel $\theta \left(t\right)=\sum_{\ell =1,2}\left(J_{\theta ,\ell }/\tau_{\theta ,\ell }\right)e^{-t/\tau_{\theta ,\ell }}$ for *t* ≥ *t*_ref_ with *J*_*θ*,1_ = 1.5 mV⋅s, *τ*_*θ*,1_ = 0.01 s, *J*_*θ*,2_ = 1.5 mV⋅s, *τ*_*θ*,2_ = 1 s. (D) Bursty neuron model. (i) Biphasic threshold kernel *θ*(*t*), where a combination of a negative part (facilitation) and a positive part (adaptation) yields a bursty spike pattern, (ii) sample firing pattern of one neuron. (iii) The interspike interval distribution with values of rate and CV. (E) Power spectrum of the population activity *A*_*N*_(*t*) shown in (D)-(iv). Parameters in (D) and (E): *μ* = 20, *u*_th_ = 10 mV, *u*_r_ = 0 mV, *τ*_m_ = 0.01 s, facilitation: *J*_*θ*,1_ = −0.45 mV⋅s, *τ*_*θ*,1_ = 0.05 s; adaptation: *J*_*θ*,2_ = 2.5 mV⋅s, *τ*_*θ*,2_ = 1 s.

Further important spike-history effects can be realized by a dynamic threshold. For instance, spike-frequency adaptation, where a neuron adapts its firing rate in response to a step current after an initial strong response (Fig 4A and 4B), can be modeled by an accumulating threshold that slowly decays between spikes [47, 49]. In single realizations, the mean population rate as well as the size of fluctuations appear to be similar for microscopic and mesoscopic case (Fig 4B, top and bottom, respectively). For a more quantitative comparison we compared the ensemble statistics as quantified by the power spectrum. This comparison reveals that the fluctuation statistics is well captured by the mesoscopic model (Fig 4C). The main effect of adaptation is a marked reduction in the power spectrum at low frequencies compared to the non-adaptive neurons of Fig 3. The small discrepancies compared to the microscopic simulation originate from the quasi-renewal approximation, which does not account for the individual spike history of a neuron before the last spike but only uses the population averaged history. This approximation is expected to work well if the threshold kernel changes slowly, effectively averaging the spike history locally in time [63].

In the case of fast changes of the threshold kernel, we do not expect that the quasi-renewal approximation holds. For example, a biphasic kernel [95] with a facilitating part at short interspike intervals (ISI) and an adaptation part for large ISIs (Fig 4D-(i)) can realize bursty spike patterns (Fig 4D-(ii)). The burstiness is reflected in the ISI density by a peak at small ISIs, corresponding to ISIs within a burst, and a tail that extends to large ISIs representing interburst intervals (Fig 4D-(iii)). Remarkably, the mesoscopic equations with the quasi-renewal approximation qualitatively capture the burstiness, as can be seen by the strong low-frequency power at about 1 Hz (Fig 4E). At the same time, the effect of adaptation manifests itself in a reduced power at even lower frequencies. The systematic overestimation of the power across frequencies implies a larger variance of the empirical population activity obtained from the mesoscopic simulation, which is indeed visible by looking at the single realizations (Fig 4D-(iv)). As an aside, we note that facilitation which is strong compared to adaptation can lead to unstable neuron dynamics even for isolated neurons [96].

#### Recurrent network of randomly connected neurons

**Fig 5:**
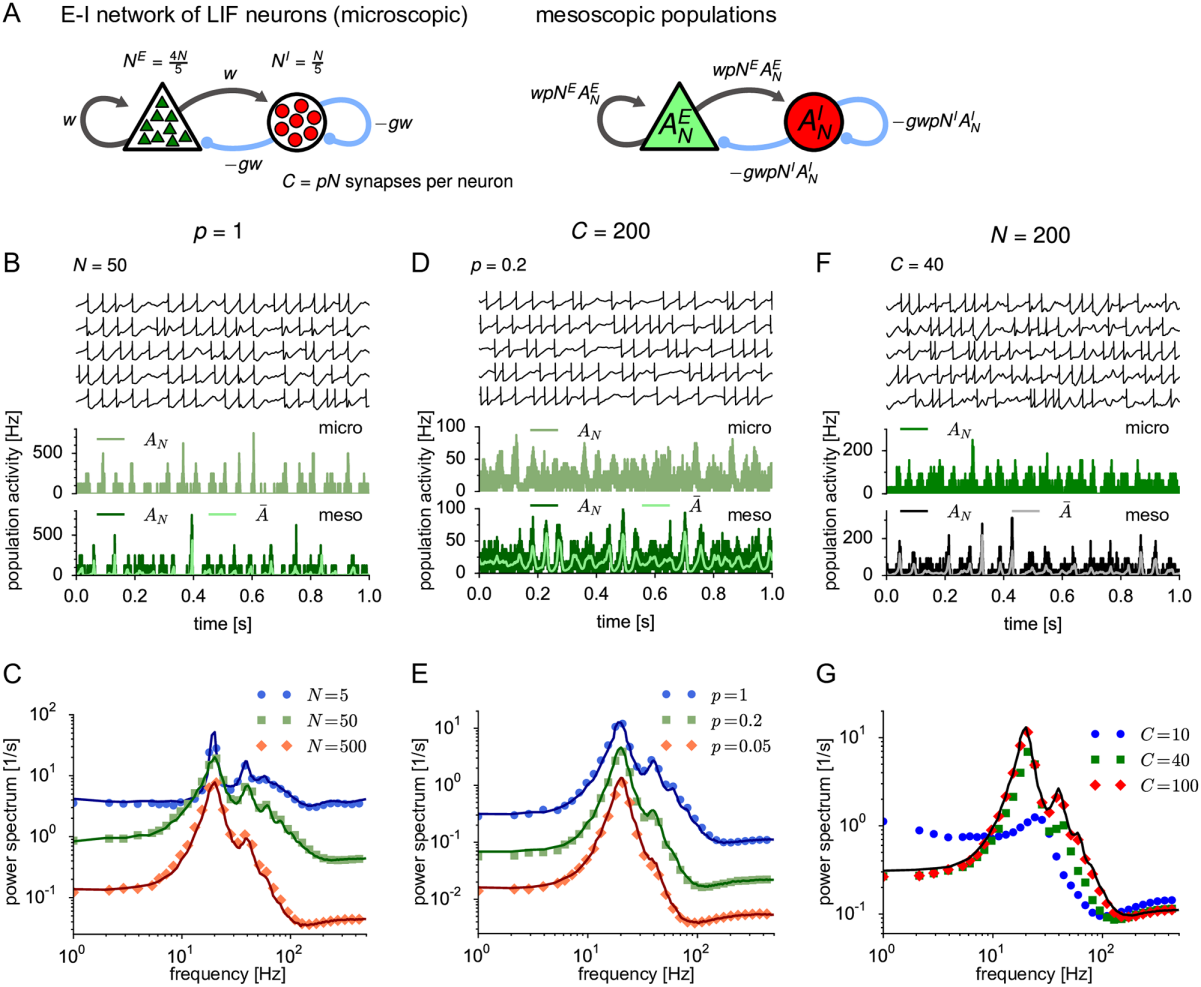
Mesoscopic dynamics of E-I network for varying network size *N*, connection probability *p* and number of synapses per neuron *C*. (A) Left: Schematic of the network of *N*^*E*^ excitatory and *N*^*I*^ = *N*^*E*^/4 inhibitory leaky integrate-and-fire (LIF) neurons, each receiving *C*^*E*^ = *pN*^*E*^ (*C*^*I*^ = *pN*^*I*^) connections from a random subset of excitatory (inhibitory) neurons. Total numbers are *N* = *N*^*E*^ + *N*^*I*^ and *C* = *C*^*E*^ + *C*^*I*^. At *C* = 200, the synaptic strength is *w* = 0.3 mV and −*gw* = −1.5 mV for excitatory and inhibitory connections, respectively. To preserve a constant mean synaptic input, the synaptic strength is scaled such that *Cw* = *const*‥ Right: Schematic of a corresponding mesoscopic model of two interacting populations. (B) Trajectories of *u*(*t*) for five example neurons (top) and of the excitatory population activity $A_{N}^{E}\left(t\right)$ obtained from the network simulation (middle) and the mesoscopic simulation (bottom, dark green) for *C* = *N* = 50; time resolution Δ*t* = 0.2 ms. The light green trajectory (bottom panel) depicts the expected population activity ${\bar{A}}^{E}\left(t\right)$ given the past activity. (C) Power spectra of $A_{N}^{E}\left(t\right)$ for different network sizes while keeping *p* = 1 fixed (microscopic: symbols, mesoscopic: solid lines with corresponding dark colors). (D) Sample trajectories corresponding to the green curve in (E) (*N* = 1000, *p* = 0.2). (E) Analogously to (C) but varying the connection probabilities while keeping *C* = *pN* = 200 fixed. (F) Sample trajectories corresponding to the green curve in (G) (*N* = 200, *C* = 40). (G) Analogously to (C) but varying the number of synapses *C* while keeping *N* = 200 fixed. Note that the mesoscopic theory (black solid line) is independent of *C* because the product *Cw*, which determines the interaction strength in the mesoscopic model (see, left panel of (A)), is kept constant. Parameters: *μ*^*E*/*I*^ = 24 mV, $\Delta_{u}^{E/I}=2.5$ mV and *θ*^*E*/*I*^(*t*) ≡ 0 (no adaptation).

So far, we have studied populations of uncoupled neurons. This allowed us to demonstrate that the mesoscopic dynamics captures effects of single neuron dynamics on the fluctuations of the population activity. Let us now suppose that each neuron in a population *α* is randomly connected to presynaptic neurons in population *β* with probability *p*^*αβ*^ such that the in-degree is fixed to *p*^*αβ*^ *N*^*β*^ connections. In the presence of synaptic coupling, the fluctuations at time *t* are propagated through the recurrent connectivity and may significantly influence the population activity at time *t*′ > *t*. For instance, in a fully-connected network (*p*^*αβ*^ = *p* = 1 for all *α* and *β*) of excitatory and inhibitory neurons (E-I network, Fig 5B and 5C), all neurons within a population receive identical inputs given by the population activities $A_{N}^{\alpha }\left(t\right)$ (cf. Eq (29)). Finite-size fluctuations of $A_{N}^{\alpha }\left(t\right)$ generate common input to all neurons and tend to synchronize neurons. This effect manifests itself in large fluctuations of the population activity (Fig 5B). Since the mean-field approximation of the synaptic input becomes exact in the limit *p* → 1, we expect a good match between the microscopic and mesoscopic simulation in this case. Interestingly, the power spectra of the population activities obtained from these simulations coincide well even for an extremely small E-I network consisting of only one inhibitory and four excitatory neurons (Fig 5C). This extreme case of *N* = 5 neurons with strong synapses (here, *w*^*EE*^ = *w*^*IE*^ = 12 mV, *w*^*II*^ = *w*^*EI*^ = −60 mV) highlights the non-perturbative character of our theory for fully-connected networks, which does not require the inverse system size or the synaptic strength to be small. In general, the power spectra reveal pronounced oscillations that are induced by finite size fluctuations [43]. The amplitude of these stochastic oscillations decreases as the network size increases and vanishes in the large-*N* limit.

**Fig 6:**
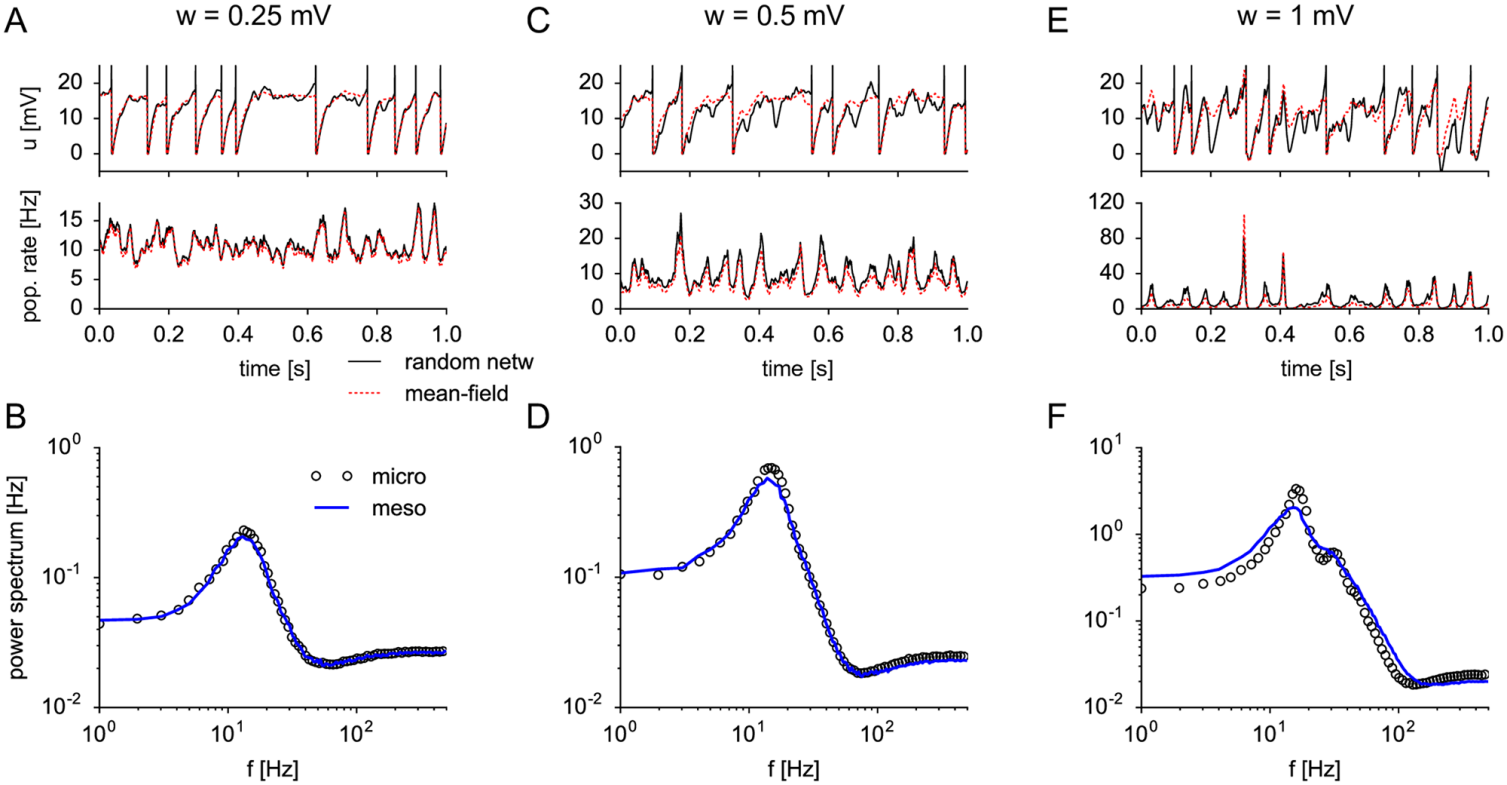
Mean-field approximation of synaptic input for randomly connected networks. The same E-I network as in Fig 5 with *N* = 500 neurons and connection probability *p* = 0.2 was simulated for increasing synaptic strength *w*^*EE*^ = *w*^*IE*^ = *w* (*w*^*EI*^ = *w*^*II*^ = −5*w*) of excitatory (inhibitory) connections: (A, B) *w* = 0.25 mV, (C, D) *w* = 0.5 mV (E, F) *w* = 1 mV. (A, C, E) Top: Membrane potential of one example neuron shows fluctuations due to spike input from *C* = 100 presynaptic neurons (black line), which represent a random subset of all 500 neurons. The mean-field approximation of the membrane potential (dashed red line) assumes that the neuron had the same firing times but was driven by all neurons, i.e. by the population activities $A_{N}^{E}\left(t\right)$ and $A_{N}^{I}\left(t\right)$, with rescaled synaptic strength $w_{\text{MF}}^{E/I}=pw^{E/I}$. Although individual membrane potentials differ significantly from the mean-field approximation (top), the relevant population-averaged hazard rates ${\bar{A}}_{\text{micro}}^{E/I}\left(t\right)\equiv \frac{1}{N^{E/I}}\sum_{i=1}^{N^{E/I}}\lambda \left(t|{\hat{t}}_{i}\right)$ (bottom) are well predicted by the mean-field approximation. (B, D, F) Corresponding power spectra of the (excitatory) population activity for microscopic (circles) and mesoscopic (blue solid line) simulation. Parameters as in Fig 5 except *μ*^*E*/*I*^ = 18 mV.

If the network is not fully but randomly-connected (0 < *p* < 1), neurons still share a part of the finite-size fluctuations of the population activity. Earlier theoretical studies [58, 68, 97] have pointed out that these common fluctuations inevitably yield correlated and partially synchronized neural activity, as observed in simulations (Fig 5D and 5F). This genuine finite-size effect decreases for larger networks approaching an asynchronous state [98] (Fig 5C and 5E). As argued in previous studies [58–60], the fluctuations of the synaptic input can be decomposed into two components, a coherent and an incoherent one. The coherent fluctuations are given by the fluctuations of the population activity and are thus common to all neurons of a population. This component is exactly described by our mean-field approximation, $u_{i}^{\alpha }\left(t\right)\approx u_{A}\left(t,{\hat{t}}_{i}^{\alpha }\right)$ used in Eq (4) (cf. Methods, Eq (31)). The incoherent fluctuations are caused by the quenched randomness of the network (i.e. each neuron receives input from a different subpopulation of the network) and have been described as independent Poisson input in earlier studies [58–60]. If we compare the membrane potential of a *single* neuron with the one expected from the mean-field approximation (Fig 6A, 6C and 6E, top), we indeed observe a significant difference in fluctuations. This difference originates from the incoherent component. Differences in membrane potential will lead to differences in the instantaneous spike emission probability for each individual neuron; cf. Eq (3). However, in order to calculate the population activity we need to average the conditional firing rate of Eq (3) over all neurons in the population (see Methods, Eq (28) for details). Despite the fact that each neuron is characterized by a different last firing time $\hat{t}$, the differences in firing rate caused by voltage fluctuations will, for sufficiently large *N* and not too small *p*, average out whereas common fluctuations caused by past fluctuations in the population activity will survive (Fig 6A, 6C and 6E, bottom). In other words, the coherent component is the one that dominates the finite-N activity whereas the incoherent one is washed out. Therefore, mesoscopic population activities can be well described by our mean-field approximation even when the network is not fully connected (Figs 5E, 5G and 6B, 6D, 6F). Remarkably, even for *C* = 200 synapses per neuron and *p* = 0.05, the mesoscopic model agrees well with the microscopic model. However, if both *N* and *p* are small, the mesoscopic theory breaks down as expected (Fig 5G, blue circles). Furthermore, strong synaptic weights imply strong incoherent noise, which is then passed through the exponential nonlinearity of the hazard function. This may lead to deviations of the population-averaged hazard rate from the corresponding mean-field approximation (Fig 6E, bottom), and, consequently, to deviations between microscopic and mesoscopic population activities in networks with strong random connections (Fig 6F).

#### Finite-size induced switching in bistable networks

In large but finite E-I networks, the main effect of weak finite-size fluctuations is to distort the deterministic population dynamics of the infinitely large network (stable asynchronous state or limit cycle motion) leading to stochastic oscillations and phase diffusion that can be understood analytically by linear response theory [43, 55, 60, 69] and weakly nonlinear analysis [58]. This is qualitatively different in networks with multiple stable states. In such networks, finite-size fluctuations may have a drastic effect because they enable large switch-like transitions between metastable states that cannot be described by a linear or weakly nonlinear theory. We will show now that our mesoscopic population equation accurately captures strongly nonlinear effects, such as large fluctuations in multistable networks.

**Fig 7:**
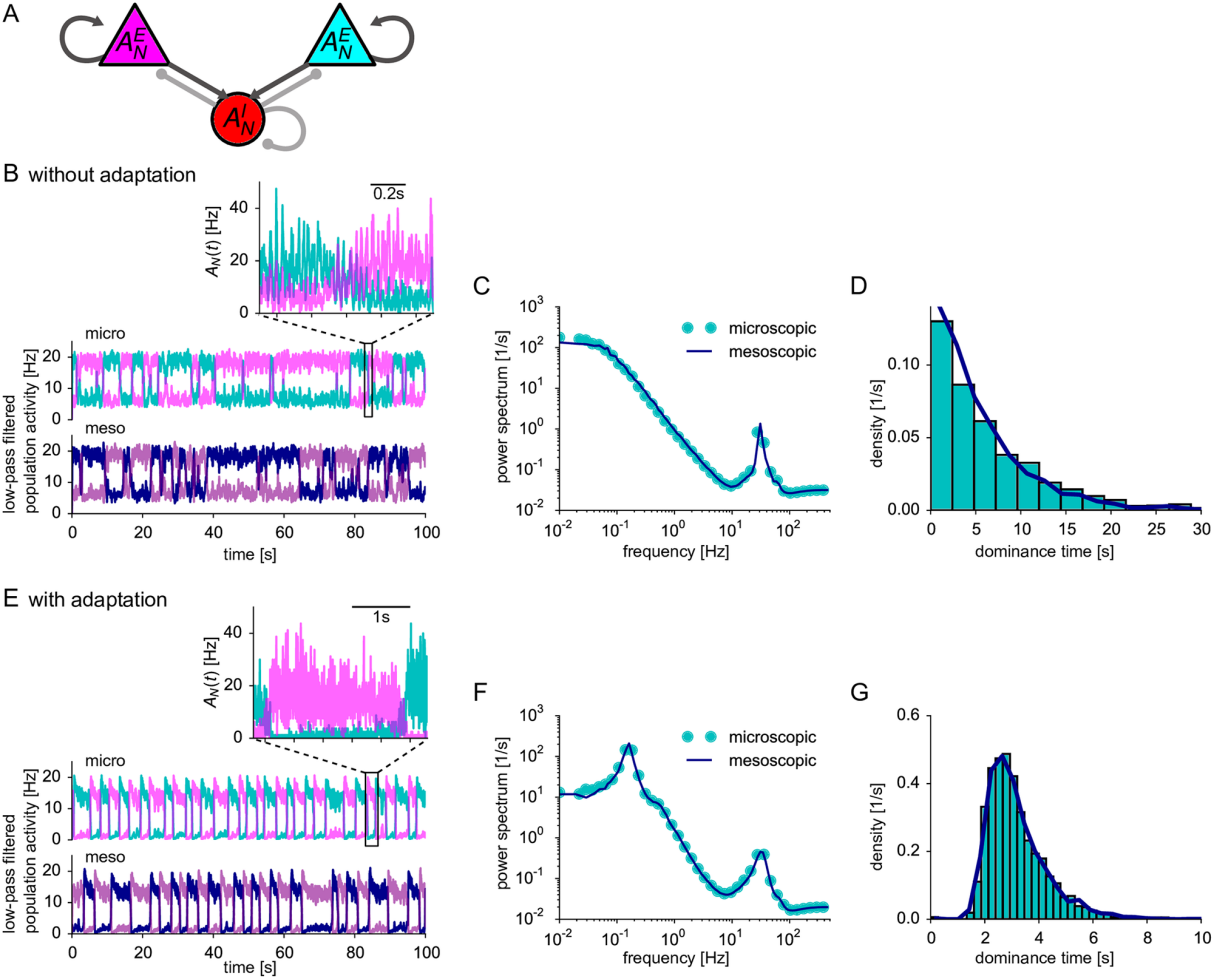
Finite-size induced switching in a bistable network. (A) Schematic of a winner-take-all network architecture: Two competing excitatory populations (*N*^*E*1^ = *N*^*E*2^ = 400) interact with a common inhibitory population (*N*^*I*^ = 200). (B)-(D) In the absence of adaptation (*θ*^*E*/*I*^(*t*) ≡ 0), the excitatory populations switch between low and high activities in an irregular fashion (B). Activities in (B, E) are low-pass-filtered by a moving average of 100 ms. Top: full network simulation. Inset: Magnified view of the activities for 1 s (without moving average) showing fast large-amplitude oscillations. Bottom: mesoscopic simulation. (C) The power spectrum of the activity of the excitatory populations exhibits large low-frequency power and a high-frequency peak corresponding to the slow stochastic switching between high- and low-activity states and the fast oscillations, respectively. (D) The density of the dominance times (i.e. the residence time in the high-activity states) has an exponential form. (E-G) Like (B-D) but excitatory neurons exhibit weak and slow adaptation ($\theta^{E}\left(t\right)=\left(J_{\theta }/\tau_{\theta }\right)e^{-t/\tau_{\theta }}$ with *J*_*θ*_ = 0.1 mV⋅s, *τ*_*θ*_ = 1 s for *t* ≥ *t*_ref_). Switching between high- and low-activity states is more regular than in the non-adapting case as revealed by a low-frequency peak in the power spectrum (F) and a narrow, unimodal density of dominance times (G). In (C, D) and (F, G) microscopic and mesoscopic simulation correspond to cyan symbols/bars and dark blue solid lines, respectively. Parameters: *μ*^*E*/*I*^ = 36 mV except for *μ*^*E*^ = 36.5 in (E-G) to compensate adaptation. Time step Δ*t* = 0.01 ms (microscopic), Δ*t* = 0.2 ms (mesoscopic). Efficacies of excitatory and inhibitory connections: *w*^*E*^ = 0.0624 mV and *w*^*I*^ = −0.2496 mV (B-D), and *w*^*E*^ = 0.096 mV and *w*^*I*^ = −0.384 mV (E-G), *p* = 1, $\Delta_{u}^{E/I}=2.5$ mV.

Multistability in spiking neural networks can emerge as a collective effect in balanced E-I networks with clustered connectivity [99], and, generically, in networks with a winner-take-all architecture, where excitatory populations compete through inhibitory interactions mediated by a common inhibitory population (see, e.g. [14, 100–104] and Fig 7A). Jumps between metastable states have been used to model switchings in bistable perception [32–34]. To understand such finite-size induced switching in spiking neural networks on a qualitative level, phenomenological rate models have been usually employed [32, 104]. In these models, stochastic switchings are enabled by noise added to the deterministic rate equations in an *ad hoc* manner. Our mesoscopic mean-field equations keep the spirit of such rate equations, however with the important difference that the noisy dynamics is systematically derived from the underlying spiking neural network without any free parameter. Here, we show that the mesoscopic mean-field equations *quantitatively* reproduce finite-size induced transitions between metastable states of spiking neural networks. We emphasize that the switching statistics depends sensitively on the properties of the noise that drive the transitions [105]. Therefore, an accurate account of finite-size fluctuations is expected to be particularly important in this case.

We consider a simple bistable network of two excitatory populations with activities *A*^*E*1^ and *A*^*E*2^, respectively, that are reciprocally connected to a common inhibitory population with activity *A*^*I*^ (Fig 7A). We choose the mean input and the connection strength such that in the large-*N* limit the population activities exhibited two stable equilibrium states, one corresponding to a situation, where *A*^*E*1^ is high and *A*^*E*2^ is low, the other state corresponding to the inverse situation, where *A*^*E*1^ is low and *A*^*E*2^ is high. We found that in smaller networks, finite-size fluctuations are indeed sufficient to induce transitions between the two states leading to repeated switches between high- and low-activity states (Fig 7B and 7E). The regularity of the switching appears to depend crucially on the presence of adaptation, as has been suggested previously [32, 34]. Remarkably, both in the presence and absence of adaptation, the switching dynamics of the spiking neural network appears to be well reproduced by the mesoscopic mean-field model.

For a more quantitative comparison, we use several statistical measures that characterize the bistable activity. Let us first consider the case without adaptation. As before, we compare the power spectra of the population activity for both microscopic and mesoscopic simulation and find a good agreement (Fig 7C). The peak in the power spectrum at relatively high-frequency reveals strong, rapid oscillations that are visible in the population activity after a switch to the high-activity state (inset of Fig 7B with magnified view). In contrast, the large power at low frequencies corresponds to the slow fluctuations caused by the switching of activity between the two excitatory populations, as revealed by the low-pass filtered population activity (Fig 7B). The Lorentzian shape of the power spectrum caused by the slow switching dynamics is consistent with stochastically independent, exponentially distributed residence times in each of the two activity states (i.e., a homogeneous Poisson process). The residence time distribution shows indeed a monotonic, exponential decay (Fig 7D) both in the microscopic and mesoscopic model. Furthermore, we found that residence times do not exhibit significant serial correlations. Together, this confirms the Poissonian nature of bistable switching in our three-population model of neurons without adaptation.

In models for perceptual bistability, residence times in the high-activity state are often called dominance times. The distribution of dominance times is usually not exponential but has been described by a more narrow, gamma-like distribution (see, e.g. [106]). Such a more narrow distribution emerges in a three-population network where excitatory neurons are weakly adaptive. When the population enters a high-activity state, the initial strong increase of the population activity is now followed by a slow adaptation to a somewhat smaller, stationary activity (Fig 7E). Eventually, the population jumps back to the low-activity state. The switching dynamics is much more regular with than without adaptation leading to slow stochastic oscillations as highlighted by a second peak in the power spectrum at low frequencies (Fig 7F) and a narrow distribution of dominance times (Fig 7G), in line with previous theoretical studies [32–34]. We emphasize, however, that in contrast to these studies the underlying deterministic dynamics for *N* → ∞ is in our case not oscillatory but bistable, because the adaptation level is below the critical value necessary in the deterministic model to switch back to the low-activity state.

The emergence of regular switching due to finite-size noise can be understood by interpreting the residence time of a given population in the high-activity state as arising from two stages: (i) the initial transient of the activity to a decreased (but still large) stationary value due to adaptation and (ii) the subsequent noise-induced escape from the stationary adapted state. The first stage is deterministic and hence does not contribute to the variability of the residence times. The variability results mainly from the second stage. The duration of the first stage is determined by the adaptation time scale. If this time covers a considerable part of the total residence time, we expect that the coefficient of variation (CV), defined as the ratio of standard deviation and mean residence time, is small. In the case without adaptation, a deterministic relaxation stage can be neglected against the mean noise-induced escape time so that the CV is larger.

#### Mesoscopic dynamics of cortical microcolumn

As a final example, we applied the mesoscopic population equations to a biologically more detailed model of a local cortical microcircuit. Specifically, we used the multi-laminar column model of V1 proposed by Potjans and Diesmann [5] (see also [72, 107] for an analysis of this model). It consists of about 80′000 non-adapting integrate-and-fire neurons organized into four layers (L2/3, L4, L5 and L6), each accommodating an excitatory and an inhibitory population (see schematic in Fig 1A). The neurons are randomly connected within and across the eight populations. We slightly changed this model to include spike-frequency adaptation of excitatory neurons, as observed in experiments (see e.g. [26]). Furthermore, we replaced the Poissonian background noise in the original model by an increase of mean current drive and escape noise (both in the microscopic and mesoscopic model). The mean current drive was chosen such that the firing rates of the spontaneous activity were matched to the firing rates in the original model. We note that the fitting of the mean current was made possible by the use of our population equations, which allow for an efficient evaluation of the firing rates. The complete set of parameters is listed in Methods, Sec. “Modified Potjans-Diesmann model”.

**Fig 8:**
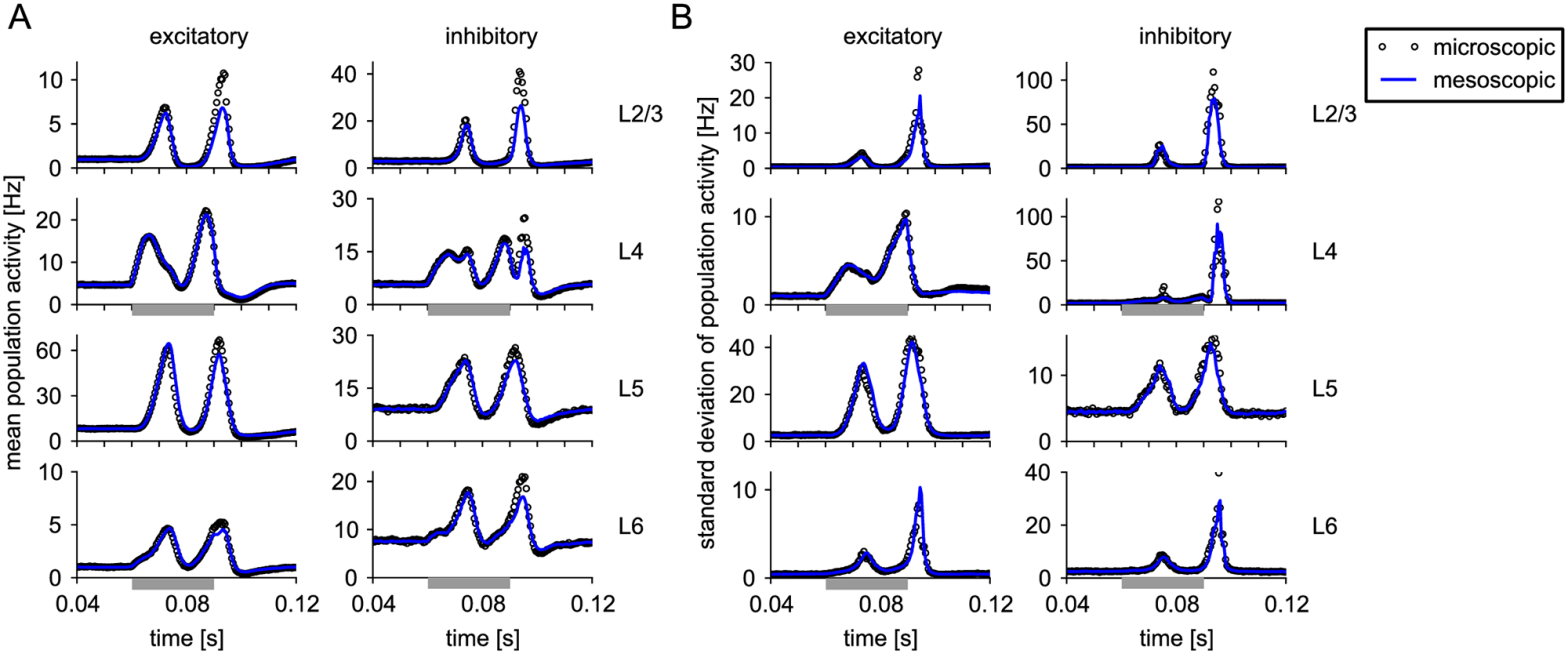
Time-dependent statistics of the population activities in a cortical column model. (A) Trial-averaged population activities (peri-stimulus-time histogram, PSTH) in the modified Potjans-Diesmann model as illustrated for a single trial in Fig 1. Circles and blue solid line show microscopic simulation (250 trials, simulation time step Δ*t* = 0.01 ms) and mesoscopic simulation (1000 trials, Δ*t* = 0.5 ms), respectively. A step current mimicking thalamic input is provided to neurons in layer 4 and 6 during a time window of 30 ms as indicated by the gray bar. Rows correspond to the layers L2/3, L4, L5 and L6, respectively, as indicated. Columns correspond to excitatory and inhibitory populations, respectively. (B) Corresponding, time-dependent standard deviation of *A*_*N*_(*t*) measured with temporal resolution Δ*t* = 0.5 ms.

**Fig 9:**
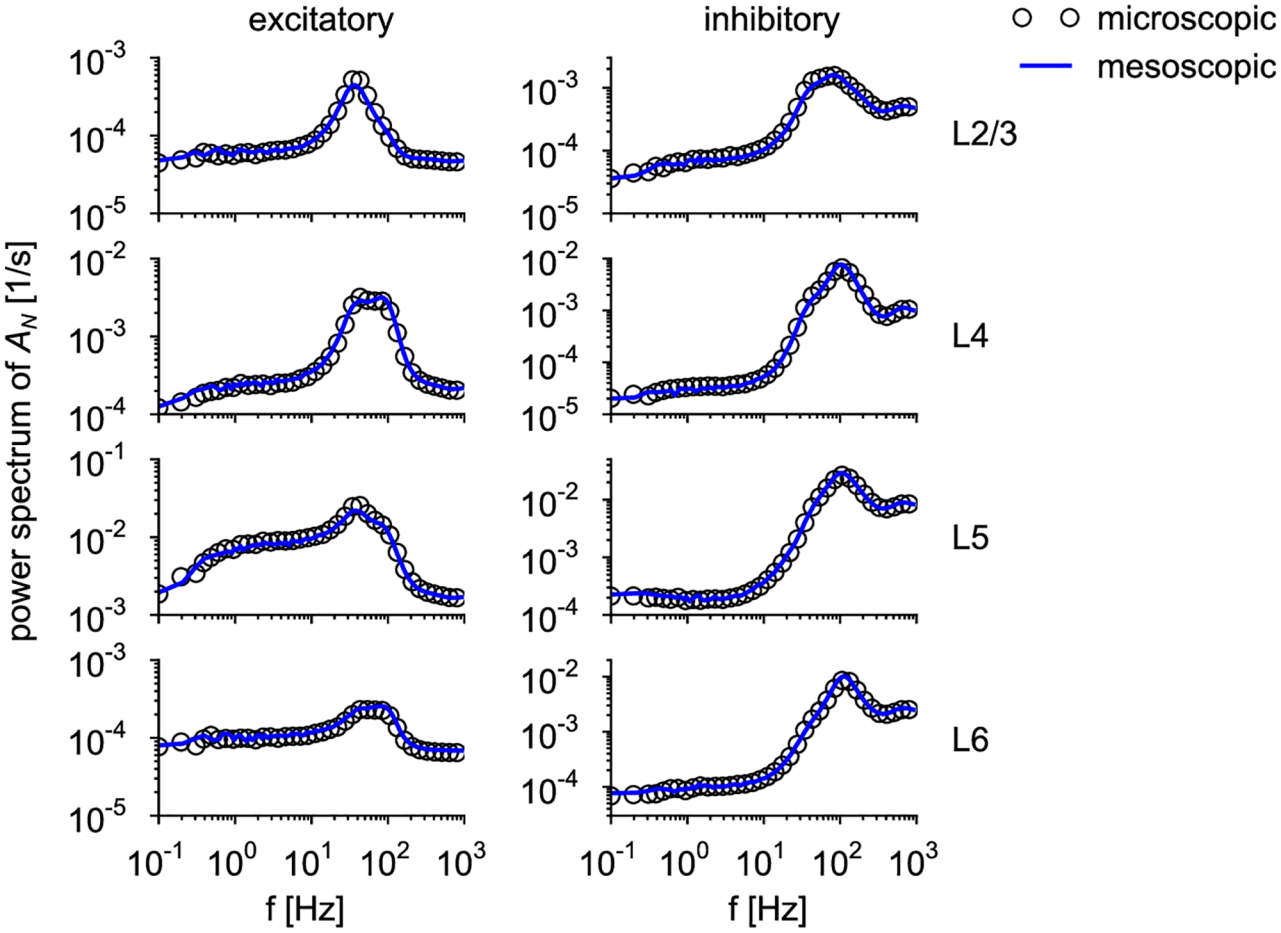
Stationary statistics of population activities in a cortical column model. Power spectra of the spontaneous population activities *A*_*N*_(*t*) in the modified Potjans-Diesmann model in the absence of time-dependent thalamic input (corresponding to the activities shown in Fig 1B (microscopic) and Fig 1D (mesoscopic) outside of the stimulation window. Circles and blue solid lines represent microscopic and mesoscopic simulation, respectively. Rows correspond to the layers L2/3, L4, L5 and L6, respectively, as indicated. Columns correspond to excitatory and inhibitory populations, respectively.

Sample trajectories of the population activities have already served as an illustration of our approach in Fig 1, where neurons in layer 4 and 6 are stimulated by a step current of 30 ms duration, mimicking input from the thalamus as in the original study [5]. Individual realizations obtained from the microscopic and mesoscopic simulation differ due to the marked stochasticity of the population activities (Fig 1B and 1D). However, trial-averaging reveals that the mean time-dependent activities that can be estimated from a peri-stimulus-time histogram (PSTH) obtained from microscopic and mesoscopic simulations indeed agree well, except for a slight underestimation of the oscillatory peak during stimulus offset compared to the microscopic simulation (Fig 8A). However, during the short moments where the mean time-dependent activity (PSTH) of the mescoscopic and microscopic simulation do not match, the time-dependent standard deviation across hundreds of trials (Fig 8B) is extremely high in both mesoscopic and microscopic simulation, indicating that fluctuations of the activity between one trial and the next are high after stimulus offset at 0.09s. The standard deviation as a function of time (Fig 8B) agrees overall nicely between microscopic and mesoscopic simulation, suggesting a good match of second-order statistics. A closer look at the second-order statistics, as provided by the power spectra of spontaneous activities (“ground state” of cortical activity), also reveals a good agreement at all frequencies (Fig 9). This agreement is remarkable in view of the low connection probabilities (*p* < 0.14, see table 5 in [5]) that violate the assumption of dense random connectivity used in the derivation of the mesoscopic mean-field equations. More generally, this example demonstrates that the range of validity of our mesoscopic theory covers relevant cortical circuit models.

Finally, we mention that the numerical integration of the mesoscopic population equations yields a significant speed-up compared to the microscopic simulation. While a systematic and fair comparison of the efficiencies depends on many details and is thus beyond the scope of this paper, we note that a simulation on a single core of 10s of biological time took 811.2s using the microscopic model, whereas that of the mesoscopic model only took 6.6s. This corresponds to a speed-up factor of around 120 achieved by using the mesoscopic population model. In the simulation, we employed the same integration time step of Δ*t* = 0.5 ms for both models for a first naive assessment of the performance. However, a more detailed comparison of the performance should be based on simulation parameters that achieve a given accuracy. In this case, we expect an even larger speed-up of the mesoscopic simulation because for the same accuracy the temporally coarse-grained population equations allow for a significantly larger time step than the microscopic simulation of spiking neurons.

## Discussion

In the present study we have derived stochastic population equations that govern the evolution of mesoscopic neural activity arising from a finite number of stochastic neurons. To our knowledge, this is the first time that such a mesoscopic dynamics has been derived from an underlying microscopic model of spiking neurons with pronounced spike-history effects. The microscopic model consists of interacting homogeneous populations of generalized integrate-and-fire (GIF) neuron models [14, 26–28], or alternatively, spike-response (SRM) [14] or generalized linear models (GLMs) [55, 108, 109]. These classes of neuron models account for various spike-history effects like refractoriness and adaptation [14, 84]. Importantly, parameters of these models can be efficiently extracted from single cell experiments [27] providing faithful representations of real cortical cells under somatic current injection. The resulting population equations on the mesoscopic level yield the expected activity of each population at the present time as a functional of population activities in the past. Given the expected activities at the present time, the actual mesoscopic activities can be obtained by drawing independent random numbers. The derived mesoscopic dynamics captures nonlinear emergent dynamics as well as finite-size effects, such as noisy oscillations and stochastic transitions in multistable networks. Realizations generated by the mesoscopic model have the same statistics as the original microscopic model to a high degree of accuracy (as quantified by power spectra and residence time distributions). The equivalence of the population dynamics (mesoscopic model) and the network of spiking neurons (microscopic model) holds for a wide range of population sizes and coupling strengths, for time-dependent external stimulation, random connectivity within and between populations, and even if the single neurons are bursty or have spike-frequency adaptation.

### Quantitative modeling of mesoscopic neural data: Applications and experimental predictions

Our theory provides a general framework to replace spiking neural networks that are organized into homogeneous populations by a network of interacting mesoscopic populations. For example, the excitatory and inhibitory neurons of a layer of a cortical column [5] may be represented by one population each, as in Fig 1. Weak heterogeneity in the neuronal parameters are allowed in our theory because the mesoscopic equations describe the population-averaged behavior. Further subdivisions of the populations are possible, however, such as a subdivision of the inhibitory neurons into fast-spiking and non fast-spiking types [26]. Populations that show initially a large degree of heterogeneity can be further subdivided into smaller populations. In this case, a correct description of finite-size fluctuations, as provided by our theory, will be particularly important. However, as with any mean-field theory, we expect that our theory breaks down if neural activity and information processing is driven by a few “outlier” neurons such that a mean-field description becomes meaningless. Further limitations may result from the mean-field and quasi-renewal approximation, Eq (4). Formally, the mean-field approximation of the synaptic input requires dense connectivity and the heterogeneity in synaptic efficacies and in synapse numbers to be weak. Moreover, the quasi-renewal approximation assumes slow threshold dynamics. However, as we have demonstrated here, our mesoscopic population equations can provide in concrete applications excellent predictions even for sparse connectivity (Figs 5D–5G, 8 and 9) and may qualitatively reproduce the mesoscopic statistics in the presence of fast threshold dynamics (Fig 4D and 4E).

Using our mesoscopic population equations it is possible to make specific predictions about the response properties of local cortical circuits. For instance, recent progress in genetic methods now enables experimentalists to selectively label and record from genetically identified cell types, such as intratelencephalic (IT), pyramidal tract (PT) and corticothalamic (CT) neurons among the excitatory neurons, and vasoactive intestinal peptide (VIP), somatostatin (Sst) and parvalbumin (Pvalb) expressing neurons among the interneurons [4]. These cell types have received much attention recently as it has been proposed that they may form a basic functional module of cortex, the canonical circuit [4, 110]. The genetic classification of cells defines subpopulations of the cortical network. A model of the canonical circuits of the cortex in terms of interacting mesoscopic populations can be particularly useful if used to describe experiments that use optogenetic stimulation of genetically-defined populations by light, which in our framework can be represented as a transient external input current. To build a mesoscopic population model based on our theory demands some assumptions about microscopic parameters such as (i) typical neuron parameters for each subpopulation, (ii) structural parameters as characterized by average synaptic efficacies and time scales of connections between and within populations, and (iii) estimates of neuron numbers per subpopulation. Parameters for a typical neuron of each population could be extracted by the efficient fitting procedures presented in [26, 27]. Structural parameters and neuron numbers have been estimated, for instance, for barrel columns in rodents somato-sensory cortex [3, 73] and other studies (see e.g., [5]). Our population equations could then be used to make predictions about circuit responses to light stimuli, e.g. by imaging the activity of a genetically-defined subpopulation in one column in response to optogenetic stimulation of another cell class in another column.

As a first step in this direction, we have demonstrated here that our population equations correctly predict the mesoscopic activities (means and fluctuations) of a simulation of a detailed, microscopic network model of a cortical microcircuit [5] under thalamic stimulation of layer 4 and 6 neurons. Using a population density method, mean activities of this model have also been predicted in a recent study to analyze its computational properties [107] with a special focus on predictive coding. Our population density approach goes beyond that study by also predicting finite-size fluctuations of the activities and their effects on the mesoscopic network dynamics such as finite-size induced stochastic oscillations. Predicting activities in real experiments is, however, complicated by the fact that the parameters of a microscopic network model are typically underconstrained given the lack or uncertainty of measured or estimated parameters [111]. Here, our population equations provide an efficient means to constrain unknown microscopic parameters by requiring consistence with mesoscopic experimental data.

While the canonical circuit represents a model of interacting populations on the mesoscopic level, recent interest in macroscopic models of entire brain areas or even of whole brains has risen [6, 7]. Population dynamics can be used in this context as a means to reduce large parts of the macroscopic neuronal network to a system of interacting populations that is numerically manageable, and requires less detailed knowledge of synaptic connectivity (mean synaptic coupling of populations as opposed to individual synapses). However, even this information about mesoscopic network structure might not be available given that it corresponds to an *M* × *M* matrix of mean synaptic efficacies, where the number *M* of populations, or respectively cell types, might be large. In this case, our population equations can be utilized to efficiently constrain unknown structural parameters, such as synaptic weights, such that the resulting mesoscopic activities are consistent with experimental data. This leads in turn to experimentally testable predictions for synaptic connectivities. Such an approach [111] has been recently applied to a network model of primate visual cortex demonstrating the usefulness of mean-field theories for predicting structural properties of large-scale cortical networks.

An interesting complementary route for further studies is a multiscale model, in which a large-scale model is simulated in terms of reduced, mesoscopic populations but with one or several areas in focus that are simulated in full microscopic detail. As knowledge of anatomy and computational capacity increases, more and more mesoscopic populations can be replaced by a microscopic simulation, while at any time in this process the full system is represented in the model. We therefore expect our population dynamics model to be a useful tool to continuously integrate experimental data into multiscale models of whole mammalian brains.

Simplified whole brain models of interacting neuronal areas have recently been proposed [112, 113]. Furthermore, large-scale neuro imaging data are routinely modeled by phenomenological population models such as neural mass, Wilson-Cowan, or neural field models [9, 22]. Our new population dynamics theory could be used in such approaches as an accurate representation of the fluctuations of neural activity in the reduced areas. For example, in macroscopic data such as resting state fMRI, EEG or MEG, the endogenously generated fluctuations of brain activity are of major interest [113]. A fortiori the same applies to mesoscopic data such as local field potentials (LFP) or voltage-sensitive dye (VSD), in which finite-size fluctuation are expected to be large. Our theory paves the way for relating macroscale fluctuations to the underlying networks of spiking neurons and their activity, and so to the neuronal circuits that underlie the computations of the brain.

Another interesting application of our population model is to predict the activity of neural networks grown in cultures. This model system is much more accessible and controllable (e.g., by optogenetic stimulations) than cortical networks in-vivo but may still provide valuable insights into the complex network activity of excitatory and inhibitory neurons as proposed in a recent study [114]. In particular, in that study the authors propose a critical role for short-term plasticity [115]. Although we have here used static synapses, an extension of our mesoscopic mean-field theory to synaptic short-term plasticity is feasible. Furthermore, finite-size fluctuations appear to be particularly important in cell cultures as suggested by a previous theoretical study [62]. Our mesoscopic population theory thus represents a framework to predict spontaneous as well as evoked activity in neuronal cell cultures.

### Theoretical aspects

From a theoretical point of view, our study represents a generalization of *deterministic*, macroscopic population equations for an infinite number of spiking neurons with refractoriness [14, 23, 63] to *stochastic*, mesoscopic population equations for a finite number of neurons. The resulting dynamics can be directly used to generate single stochastic realizations of mesoscopic activities, in analogy to a Langevin dynamics. Our work is thus conceptually different from earlier studies of finite-size effects [66–68], who also considered finite networks of spiking neurons and refractoriness but derived deterministic evolution equations for moment and cross-correlation functions and hence characterized the ensemble dynamics. Furthermore, in contrast to these studies, our theory is not based on a perturbation expansion around the *N* → ∞ limit, and thus captures large and non-Gaussian fluctuations in strongly nonlinear population dynamics such as bistable networks.

Outside the low-rate Poisson firing regime, spiking neurons exhibit history dependencies in their spike trains, the most prominent of which is neuronal refractoriness, i.e. the strongly reduced firing probability depending on the time since the last spike. On the population level this means that a positive (negative) fluctuation of the population rate affects the underlying refractory state of the population because more (less) neurons than expected become refractory. This altered refractory state in turn tends to decrease (increase) the mean and variance of the population activity shortly after the fluctuation. More generally, fluctuations of the population activity influence the population density of state variables, which in turn influences fluctuations. In this study, we have worked out how to incorporate this interplay between fluctuations of the population activity and fluctuations of the refractory density into a mesoscopic population dynamics. The key insight to achieve this was (i) to exploit the normalization condition for the density of microscopic states (in our case, the density of last spike times) and (ii) to associate density fluctuations with a time-dependent but state-independent average rate that emphasizes the microscopic rates of those states that exhibit the largest finite-size fluctuations (in our case, the weighted average rate with respect to the variance $v(t,\hat{t})$).

Our work is thus in marked contrast to previous stochastic rate models for finite-size systems in the form of stochastic Wilson-Cowan equations [62, 104], or stochastic neural field equations [39, 116]. In these models, finite-size fluctuations of the rate may feed back through the recurrent connections but the strong negative self-feedback due to refractoriness is neglected. This is the case even if the stationary or dynamic transfer function employed in the rate dynamics corresponds to a spiking neuron model [62, 72]. Furthermore, fluctuations of the population rate have often been implemented *ad hoc* by a phenomenological white-noise source, which was added to the macroscopic (i.e. deterministic) rate dynamics [32, 102, 112]. The intensity of the noise is a free parameter in these cases. Our mesoscopic equations are also driven by a noise source, but two differences to these studies are noteworthy: First, it is derived from a microscopic model and does not contain any free parameter; and second, the noise is white *given* the predicted mean activity but since the activity predicted in one time step depends on fluctuations in all earlier time steps, the effective noise leads to a colored noise spectrum—even if coupling is removed (see Fig 3). This observation is consistent with previous studies [55, 58, 60, 69], in which the power spectrum of the fluctuations about a steady-state has been calculated analytically.

On the population level, refractoriness can be taken into account by population density equations such as the Fokker-Planck equation for the membrane potential density (see e.g. [36, 58, 59], or [107, 117, 118] for related master equations), or the population integral equation for the refractory density [14, 23, 88, 89]. These studies were mainly concerned with macroscopic populations, which formally correspond to the limit *N* → ∞. For the refractory density formalism, we have shown here how to extend the population integral equation to the case of finite population size. To this end, we corrected for the missing normalization of the mesoscopic density (e.g. $Q\left(t,\hat{t}\right)=S\left(t|\hat{t}\right)A_{N}\left(\hat{t}\right)$ in Eq (13) or $q(t,\hat{t})$ in Eq (80)), and thereby accounted for the interplay between fluctuations and refractoriness. Finite-size fluctuations of the population rate have also been used in the Fokker-Planck formalism [58–60] but the immediate effect of these fluctuations on the membrane potential density at threshold, and hence the refractoriness, has been neglected: in fact, a positive (negative) fluctuation of the population rate increases (decreases) the number of neurons at the reset potential while the number of neurons close to the threshold has to decrease (increase) such that the microscopic density remains normalized. The finite-*N* membrane potential density used by Mattia and Del Giudice [60] does not account for this normalization effect. Whereas the numerical integration of their equation may still give a satisfying solution in the low-rate, Poissonian-firing regime, where refractory effects can be neglected, it becomes unstable at higher rates unless the density is renormalized manually at every time step [119]. How to correct for the missing normalization in the Fokker-Planck approach is still an unsolved theoretical question. In this respect, using analogies to and insights from our approach might be promising.

The quasi-renewal approximation [55, 63] allowed us to develop a finite-size theory for an effectively one-dimensional population density equation even in the presence of adaptation. Here, the only microscopic state variable is the last spike time $\hat{t}$, or equivalently the age of the neuron $\tau =t-\hat{t}$. Longer lasting spike history effects such as adaptation are captured by the dependence of the conditional intensity on the population activity *A*_*N*_, which as a mesoscopic mean-field variable does not need to be treated as a state variable. Furthermore, Chizhov and Graham have shown that the one-dimensional population density method in terms of the age *τ* can also capture multiple gating variables in conductance-based neuron models with adaptation [88]. Such one-dimensional descriptions have great advantages compared to population density equations that include adaptation by additional state variables and which thus require a multi-dimensional state-space [37, 120–122]: Firstly, the numerical solution of the density equations grows exponentially with the number of dimensions and becomes quickly infeasible if multiple adaptation variables are needed as e.g. in the case of multi-timescale adaptation [28] or if an adiabatic approximation of slow variables [64, 120, 123] is not possible. Secondly, it is unclear how to treat finite-size fluctuations in the multi-dimensional case.

Our theory is based on an effective fully-connected network, in which neurons are coupled by the actual realization of the stochastic population activity (the “mean field”), both in the microscopic and mesoscopic model. Thus, in the limit of a fully-connected network, the problem of self-consistently matching the input and output statistics, which arises in mean-field theories, is automatically satisfied to any order by our finite-size theory. This is in marked contrast to the opposite limit of a sparsely-connected network [59]. In that case, the mean-field variables correspond to the *statistics* of the spike trains (e.g. rate and auto-correlation function) rather than to the actual realization of the population activity. These statistics must be matched self-consistently for input and output, which is a hard theoretical problem [56, 57, 124, 125]. Between these two limit cases, where the network is randomly connected with some finite connection probability 0 < *p* < 1, our examples (Figs 5, 8 and 9) indicate that the approximation by an effective fully-connected network can still yield reasonable results even for relatively sparse networks with *p* = 5%. We emphasize that in our microscopic network model we used a fixed in-degree in order to avoid additional variability due to the quenched randomness in the number of synapses. This allowed us to focus on dynamic finite-size noise in homogeneous populations and its interactions with refractoriness. In contrast, the heterogeneity caused by the quenched randomness is a further finite-size effect [58] that needs to be examined in a future study.

As an integral equation, the mesoscopic population model is formally infinitely dimensional and represents a non-Markovian dynamics for the population activity *A*_*N*_. Such complexity is expected given that the derived population equations are general and not limited to a specific dynamical regime. Loosely speaking, the equations must be rich enough, and hence sufficiently complex, in order to reproduce the rich repertoire of dynamical regimes that fully connected networks of spiking neurons are able to exhibit (e.g. limit cycles, multi-stability, cluster states [23]). For a mathematical analysis, however, it is often desirable to have a low-dimensional representation of the population dynamics in terms of a few differential equations, at least for a certain parameter range. Apart from the dynamics in the neighborhood of an equilibrium point (see e.g. [126]) or in the limit of slow synapses [127], such “firing rate models” are difficult to link to the microscopic model already in the deterministic (macroscopic) case (for notable exceptions see [128, 129]), let alone the stochastic, finite-size case. Here, our mesoscopic population rate equations can serve as a suitable starting point for deriving low-dimensional dynamics that links microscopic models to mesoscopic rate equations with realistic finite-size noise.

### Extensions of the model

There are several ways to extend our mesoscopic population model towards more biological realism. We already mentioned the possibility to include short-term synaptic plasticity in our mean-field framework. Furthermore, the hazard function could be generalized to capture Gaussian current noise as arising from background spiking activity [5, 57–60, 98]. Approximate mappings of white and colored current noise to an effective hazard function in the escape noise formalism are available [88, 89] and might be combined with our mesoscopic population model. Yet another extension concerns the synaptic input model. Here we only looked at current input but, as shown by Chizhov and Graham [88], it is straightforward to extend population theories of the renewal type to the case of conductance inputs. In the simplest case, the synaptic current of neuron *i* embedded in population *α* and driven by populations *β* can be modelled by a linear voltage-dependence:

$$\begin{array}{c} I_{\text{syn},i}^{\alpha }\left(t\right)=-\sum_{\beta =1}^{M}\left(u_{i}^{\alpha }\left(t\right)-E^{\alpha \beta }\right)\sum_{j\in \Gamma_{i}^{\beta }}\left(g^{\alpha \beta }*s_{j}^{\beta }\right)\left(t\right) \end{array}$$ (18)

(cf. corresponding expression Eq (22) in Methods for current-based synapses). Here, *E*^*αβ*^ is the reversal potential of a synapse from population *β*, and *g*^*αβ*^(*t*) is the conductance response (in nS) elicited by a spike of a presynaptic neuron in population *β*. The same mean-field arguments as for the current-based model carry over to the case of conductance-based synapses. For example, the membrane potential $u_{A}^{\alpha }\left(t,\hat{t}\right)$ of a current-based leaky integrate-and-fire neuron with a last spike time at time $\hat{t}$ follows the equation

$$\begin{array}{c} \tau_{\text{m}}^{\alpha }\frac{\partial u_{A}^{\alpha }}{\partial t}=-u_{A}^{\alpha }+\mu^{\alpha }\left(t\right)+\tau_{\text{m}}^{\alpha }\sum_{\beta =1}^{M}p^{\alpha \beta }N^{\beta }w^{\alpha \beta }\left(\epsilon^{\alpha \beta }*A_{N}^{\beta }\right)\left(t\right), \end{array}$$ (19)

where at $t=\hat{t}$ and during an absolute refractory period $u_{A}\left(t,\hat{t}\right)=u_{\text{r}}$ is at the reset potential (see Methods, Eq (30) for details). In the case of conductance-based input, Eq (18), we only need to replace Eq (19) by

$$\begin{array}{c} \tau_{\text{m}}^{\alpha }\frac{\partial u_{A}^{\alpha }}{\partial t}=-u_{A}^{\alpha }+\mu^{\alpha }\left(t\right)-R^{\alpha }\sum_{\beta =1}^{M}p^{\alpha \beta }N^{\beta }\left(u_{A}^{\alpha }-E^{\alpha \beta }\right)\left(g^{\alpha \beta }*A_{N}^{\beta }\right)\left(t\right). \end{array}$$ (20)

where *R*^*α*^ is the membrane resistance. How to model nonlinear voltage-dependence of synaptic currents such as N-methyl-D-aspartate (NMDA) currents within a mean-field approximation is less obvious but approximations also exist for this case [20]. It will be an interesting question for the future how well these approaches work with the finite-N theory developed in the present study. Alternatively, effective current models [130, 131] with activity-dependent, effective time constant *τ*_m_(*t*) and effective resting potential *u*_rest_(*t*) could be another solution to treat conductance inputs.

Here, we have used a discrete set of populations. In large-scale models of the brain, one often regards the spatial continuum limit, resulting in so-called stochastic neural field equations [116]. These equations represent a compact description of neural activity and do not depend on a specific discretization of space. Just as discrete firing rate models, these field equations must be considered phenomenological because the link to neuronal parameters is not clear (note however that such equations have been derived from non-spiking, two-state neuron models for *N* < ∞ [39], and from spiking models for *N* → ∞ [132, 133]). By taking the spatial continuum limit, our mesoscopic population equations can be formulated as a stochastic neural field equation that is directly derived from a finite-size, spiking neural network. It would be interesting to employ this continuous extension of our mesoscopic equations to study the effect of spike-history effects on the stochastic behavior of bumps and waves in neural fields.

A first simple comparison of the computational performance in Results, “Mesoscopic dynamics of cortical microcolumn”, demonstrated already that the mesoscopic population dynamics outperformed the microscopic simulation by a speed-up factor of around 120. In this example, the numerical integration of the population dynamics has not been particularly optimized with respect to time step Δ*t* and history length *T*. A systematic comparison under the condition of some given accuracy, has the potential for an even larger speed-up because the population equations can be integrated with a larger time step than the spiking neural network. In addition to that, we have also compared the mesoscopic model to the full microscopic simulation of the refractory density (cf. Results, “Finite-size mean-field theory”) and found a moderate enhancement in performance for sufficiently large networks (N ≳ 100). These computational aspects will be investigated in a separate study.

## Methods

### Model

#### Network setup

We consider a network of *M* populations each consisting of *N*^*α*^ interconnected neurons of the same type (the superscript *α* = 1, …, *M* labels the populations). Neuron *i* in population *α* receives *p*^*αβ*^*N*^*β*^ connections (synapses) from a random subset $\Gamma_{i}^{\beta }$ of presynaptic neurons in population *β*. Here, *p*^*αβ*^ denotes the probability for a connection from a neuron in population *β* to a neuron in population *α*. That is, the connections between any two populations are random with fixed in-degree.

Let the spike train of neuron *i* in population *α* be denoted by

$$\begin{array}{c} s_{i}^{\alpha }\left(t\right)=\sum_{k}\delta \left(t-t_{i,k}^{\alpha }\right), \end{array}$$ (21)

where $t_{i,k}^{\alpha }$ is its *k*-th spike time and *δ* denotes the Dirac *δ*-function. The neuron receives spike train input from its presynaptic partners in population *β* with a transmission delay Δ^*αβ*^ and synaptic weight *w*^*αβ*^. More precisely, the synaptic input current $I_{\text{syn},i}^{\alpha }\left(t\right)$ is modeled as a sum of post-synaptic currents caused by each spike of presynaptic neurons:

$$\begin{array}{c} R^{\alpha }I_{\text{syn},i}^{\alpha }\left(t\right)=\tau_{\text{m}}^{\alpha }\sum_{\beta =1}^{M}w^{\alpha \beta }\sum_{j\in \Gamma_{i}^{\beta }}\left(\epsilon^{\alpha \beta }*s_{j}^{\beta }\right)\left(t\right), \end{array}$$ (22)

where *R*^*α*^ and $\tau_{\text{m}}^{\alpha }$ are the membrane resistance and membrane time constant of a neuron in population *α*, respectively, and *w*^*αβ*^ sets the synaptic weights in units of mV. The synaptic kernel *ϵ*^*αβ*^(*t*) is defined as the postsynaptic current (PSC) normalized by its charge that is induced by one input spike from a neuron of population *β*. More precisely, *ϵ*^*αβ*^ is the PSC divided by its integral, and therefore it has units of 1/sec. In Eq (22), the first sum runs over all populations *β*, whereas the second sum runs over the set $\Gamma_{i}^{\beta }$ of all neurons in population *β* that project onto neuron *i* in population *α*.

In general, the filtered total synaptic input from population *β*, $\sum_{j\in \Gamma_{i}^{\beta }}\left(\epsilon^{\alpha \beta }*s_{j}^{\beta }\right)\left(t\right)$, may be modeled by a set of differential equations for a finite number of synaptic variables $y_{i,\ell }^{\alpha \beta }$, *ℓ* = 1, …, *L*. In simulations, we model the synaptic kernel by a single exponential with constant delay Δ^*αβ*^ = Δ, $\epsilon^{\alpha \beta }\left(t\right)=\Theta \left(t-\Delta \right)e^{-\left(t-\Delta \right)/\tau_{\text{s}}^{\beta }}/\tau_{\text{s}}^{\beta }$, where Θ(*t*) denotes the Heaviside step function. The synaptic time constants are $\tau_{\text{s}}^{E}=3$ ms and $\tau_{\text{s}}^{I}=6$ ms for excitatory and inhibitory synapses, respectively. This kernel can be realized by a single synaptic variable $y_{i}^{\alpha \beta }\left(t\right)$, which obeys the first-order kinetics $\tau_{\text{s}}^{\beta }{\dot{y}}_{i}^{\alpha \beta }=-y_{i}^{\alpha \beta }+\sum_{j\in \Gamma_{i}^{\beta }}s_{j}^{\beta }\left(t-\Delta \right)$ with *β* ∈ {*E*, *I*}.

#### Generalized integrate-and-fire model

Neurons are modeled by a leaky integrate-and-fire model with a dynamic threshold [14, 47, 49, 80] and an escape noise mechanism [14, 26–28]. Following [27], we refer to this model as the generalized integrate-and-fire (GIF) model. The crucial variables of this model are the membrane potential $u_{i}^{\alpha }\left(t\right)$ and the dynamic threshold $\vartheta_{i}^{\alpha }\left(t\right)$. The membrane potential obeys the subthreshold dynamics

$$\begin{array}{c} \tau_{\text{m}}^{\alpha }\frac{du_{i}^{\alpha }}{dt}=-u_{i}^{\alpha }+\mu^{\alpha }\left(t\right)+R^{\alpha }I_{\text{syn},i}^{\alpha }\left(t\right), \end{array}$$ (23)

where $\tau_{\text{m}}^{\alpha }$ is the membrane time constant and $\mu^{\alpha }\left(t\right)=u_{\text{rest}}+R^{\alpha }I_{\text{ext}}^{\alpha }\left(t\right)$ is the drive in the absence of synaptic input consisting of a constant resting potential *u*_rest_ and an external stimulus $I_{\text{ext}}^{\alpha }\left(t\right)$. The synaptic current $I_{\text{syn},i}^{\alpha }\left(t\right)$ has been defined in Eq (22).

After each spike the voltage is reset to the potential *u*_r_, where it is clamped for an absolute refractory period *t*_ref_ = 4 ms. Furthermore, each spike $t_{i,k}^{\alpha }$ adds a contribution $\theta^{\alpha }\left(t-t_{i,k}^{\alpha }\right)$ to the dynamic threshold:

$$\begin{array}{cc} \vartheta_{i}^{\alpha }\left(t\right) & =u_{\text{th}}^{\alpha }+\sum_{t_{i,j}^{\alpha }<t}\theta^{\alpha }\left(t-t_{i,k}^{\alpha }\right),\\ & =u_{\text{th}}^{\alpha }+\int_{-\infty }^{t}\theta^{\alpha }\left(t-t^{\prime }\right)s_{i}^{\alpha }\left(t^{\prime }\right)\;dt^{\prime } \end{array}$$ (24)

where $u_{\text{th}}^{\alpha }$ is a baseline threshold and *θ*^*α*^(*t*) is called the spike-triggered kernel [26, 27]. Since the increases in spike threshold accumulate over several spikes, the spike-triggered kernel causes spike-frequency adaptation. We set *θ*^*α*^(*t*) = ∞ for *t* ∈ (0, *t*_ref_) so as to ensure absolute refractoriness. For the sake of simplicity, we assumed here that all spike-triggered accumulation effects can be lumped into the threshold variable (cf. Sec. “Mapping onto a generalized linear model” below). However, if realistic membrane potentials are needed (e.g., for fitting membrane potential data [27] or in a conductance-based extension of the model (see Discussion) and [88]), adaptation mechanisms affecting the voltage should be kept in the voltage dynamics.

Spikes are elicited stochastically by a conditional intensity (also called hazard rate, escape rate or conditional rate)

$$\begin{array}{c} \lambda_{i}^{\alpha }\left(t\right)=f^{\alpha }\left(u_{i}^{\alpha }\left(t\right)-\vartheta_{i}^{\alpha }\left(t\right)\right), \end{array}$$ (25)

which depends on the momentary distance between the membrane potential and the threshold via the exponential link function $f^{\alpha }\left(x\right)=c^{\alpha }\exp\left(x/\Delta_{u}^{\alpha }\right)$. The parameter *c*^*α*^ is the escape rate at threshold and the parameter $\Delta_{u}^{\alpha }>0$ characterizes the softness of the threshold (Fig 1A, inset). Intuitively, a neuron fires immediately if its membrane potential is $2\cdot \Delta_{u}^{\alpha }$ millivolts above the threshold and is unlikely to fire if its membrane potential is $2\cdot \Delta_{u}^{\alpha }$ millivolts below the threshold [14]. In the limit $\Delta_{u}^{\alpha }\to 0$, the model turns into a deterministic (but adaptive) leaky integrate-and-fire model with a hard threshold. We emphasize that our standard choice of $\Delta_{u}^{\alpha }∼2$ mV is consistent with the intrinsic stochasticity of neurons in cortical slices [26, 85]. Alternatively, the softness of the threshold $\Delta_{u}^{\alpha }$ may also be regarded as a phenomenological parameter that accounts for all incoherent noise sources that are individual to each neuron. This includes, e.g., any intrinsic noise but also fluctuations of external background input from other neural populations that are not modeled explicitly. For instance, to account for the external Poisson input used in the original cortical column model by Potjans an Diesmann [5], we increase in Figs 1, 8 and 9 the softness to $\Delta_{u}^{\alpha }=5$ mV. We note that for more detailed comparisons with the original model, more elaborate approximations of the escape rate for the case of colored noise exist [89], which in principle could be used to approximate external Poisson noise without a free parameter. However, because such a mapping is not the focus of the current study, we stick here for the sake of simplicity to the phenomenological escape rate, Eq (25), of the exponential form.

The parameters of the model used in simulations (unless specified differently) are summarized in Table 1.

**Table 1:** Default values of parameters used in simulations unless stated otherwise.

*τ*_m_	20 ms	membrane time constant
*t*_ref_	4 ms	absolute refractory period
*u*_th_	15 mV	threshold (non-adapting part)
*u*_r_	0 mV	reset potential
*c*	10 Hz	escape rate at threshold
Δ_*u*_	2 mV	noise level
Δ	1 ms	transmission delay
$\tau_{\text{s}}^{E}$	3 ms	decay time constant of excitatory synapses
$\tau_{\text{s}}^{I}$	6 ms	decay time constant of inhibitory synapses

#### Mapping onto a generalized linear model

We also considered a slightly different variant of the model, called *spike-response model* [14] or *generalized linear model (GLM)* [55, 66, 84, 108, 109]. This model does not reqire the reset rule of the integrate-and-fire model but instead relies on spike-triggered kernels to implement refractoriness and other spike-history effects. Specifically, the membrane potential is given by

$$\begin{array}{c} u_{i}^{\alpha }\left(t\right)=h_{i}^{\alpha }\left(t\right)+\sum_{{\hat{t}}_{i,j}^{\alpha }<t}\eta^{\alpha }\left(t-{\hat{t}}_{i,j}^{\alpha }\right), \end{array}$$ (26)

where $h_{i}^{\alpha }\left(t\right)$ is the free membrane potential given by

$$\begin{array}{c} h_{i}^{\alpha }\left(t\right)=\left[\kappa^{\alpha }*\left(\mu^{\alpha }+R^{\alpha }I_{syn,i}^{\alpha }\right)\left(t\right)\right]. \end{array}$$ (27)

For a membrane filter kernel $\kappa^{\alpha }\left(t\right)=\Theta \left(t\right)e^{t/\tau_{\text{m}}^{\alpha }}/\tau_{\text{m}}^{\alpha }$, where Θ(*t*) denotes the Heaviside step function, the dynamics of $h_{i}^{\alpha }$ is equivalent to the dynamics of $u_{i}^{\alpha }$ (Eq (23)), except that $h_{i}^{\alpha }$ is not reset upon spiking. Spike-history effects on the level of the membrane potential are captured by the second term in Eq (26). This term represents the convolution $\left(\eta^{\alpha }*s_{i}^{\alpha }\right)\left(t\right)$ of the output spike train with a spike-triggered kernel *η*(*t*) and generates a spike-after-potential that accumulates over spikes. As before, the threshold $\vartheta_{i}^{\alpha }\left(t\right)$ obeys Eq (24). Given the membrane potential $u_{i}^{\alpha }\left(t\right)$ and the dynamic threshold $\vartheta_{i}^{\alpha }\left(t\right)$, spikes are generated by the same hazard rate $\lambda_{i}^{\alpha }\left(t\right)$ given by Eq (25).

At low firing rates, the spike-triggered kernel *η* can be used to approximate the integrate-and-fire dynamics by choosing $\eta \left(t\right)=\left(u_{\text{r}}-u_{\text{th}}\right)e^{-\left(t-t_{\text{ref}}^{\alpha }\right)/\tau_{\text{m}}^{\alpha }}\Theta \left(t\right)$. However, this is not an exact mapping because the value of the membrane potential is not reset to a fixed value *u*_r_ after spiking, in contrast to the GIF model. This is due to the accumulation of the threshold and the variability in the voltage at the moment of firing.

We also mention that the kernel *η* can be transformed into the kernel *θ* of the threshold dynamics [14]. This is possible because we are only interested in the spike emissions of the neurons and not the membrane potentials. In fact, the conditional firing rate, Eq (25), is invariant under the transformation *θ* → *θ* − *η*, *η* → 0.

### Mean-field approximation

An important variable that characterizes the internal state of a neuron is the time of its last spike, or, equivalently, the time elapsed since the last spike (“age” of the neuron) [88]. The time since the last spike is a good predictor of the refractory state of a neuron at time *t*. Our approach is to use a population density description for this refractory state [23, 68, 88, 89], in which the coupling of neurons as well as the adaptation of single neurons are mediated by the mesoscopic population activities $A_{N}^{\alpha }\left(t\right)$ defined by Eq (1). To this end, we replace the conditional firing rate $\lambda_{i}^{\alpha }\left(t\right)$ of a neuron *i* in population *α* by an effective rate $\lambda_{A}^{\alpha }\left(t|{\hat{t}}_{i}^{\alpha }\right)$ that only depends on its last spike time ${\hat{t}}_{i}^{\alpha }$ and the history of the population activity ${\left\{A_{N}^{\alpha }\left(t^{\prime }\right)\right\}}_{t^{\prime }<t}$ [63]. Here and in the following, the subscript *A* indicates the dependence on the history of $A_{N}^{\alpha }\left(t\right)$. We note that the expected total activity ${\bar{A}}^{\alpha }\left(t\right)$ of population *α* at time *t* is the average of all the conditional firing rates summed over all neurons in this population: ${\bar{A}}^{\alpha }\left(t\right)=\left(1/N^{\alpha }\right)\sum_{i}\lambda_{i}^{\alpha }\left(t\right)$. The effective rate $\lambda_{A}^{\alpha }\left(t|{\hat{t}}_{i}^{\alpha }\right)$ is determined such that it approximates the conditional intensity on average:

$$\begin{array}{c} \frac{1}{N^{\alpha }}\sum_{i=1}^{N^{\alpha }}\lambda_{i}^{\alpha }\left(t\right)\approx \frac{1}{N^{\alpha }}\sum_{i=1}^{N^{\alpha }}\lambda_{A}^{\alpha }\left(t|{\hat{t}}_{i}^{\alpha }\right). \end{array}$$ (28)

To find such an approximation, we proceed in two steps [55]: first, the membrane potential $u_{i}^{\alpha }\left(t\right)$ is approximated by a function $u_{A}^{\alpha }\left(t,{\hat{t}}_{i}^{\alpha }\right)$ using a mean-field approximation of the synaptic input. For fully connected populations, this first approximation turns into an exact statement. Second, the dynamic threshold $\vartheta_{i}^{\alpha }\left(t\right)$ is approximated by a function $\vartheta_{A}^{\alpha }\left(t,{\hat{t}}_{i}^{\alpha }\right)$ using the quasi-renewal approximation [63]. For renewal neurons, the second approximation becomes exact. Once we have found an expression for the mean-field approximation Eq (28), we are in a position to use a population density description with respect to the last spike times ${\hat{t}}_{i}^{\alpha }$. In the following two paragraphs we explain the above two steps in detail.

#### Mean-field approximation of synaptic input

In the special case of a fully connected network (*p*^*αβ*^ = 1), the membrane potential can be completely inferred from the last spike time ${\hat{t}}_{i}^{\alpha }$ and the mean field $A_{N}^{\alpha }$. In this case, the synaptic input Eq (22) can be rewritten as

$$\begin{array}{c} R^{\alpha }I_{\text{syn},i}^{\alpha }\left(t\right)=\tau_{\text{m}}^{\alpha }\sum_{\beta =1}^{M}p^{\alpha \beta }N^{\beta }w^{\alpha \beta }\left(\epsilon^{\alpha \beta }*A_{N}^{\beta }\right)\left(t\right). \end{array}$$ (29)

Thus, in a fully connected network all neurons in population *α* “see” the same synaptic input $R^{\alpha }I_{\text{syn}}^{\alpha }$ given by the “mean field” *A*_*N*_(*t*). From Eq (23) follows that GIF neurons with the same last spike time $\hat{t}$ all have the same membrane potential $u_{A}^{\alpha }\left(t,\hat{t}\right)$ that obeys the differential equation

$$\begin{array}{c} \tau_{\text{m}}^{\alpha }\frac{\partial u_{A}^{\alpha }}{\partial t}=-u_{A}^{\alpha }+\mu^{\alpha }\left(t\right)+\tau_{\text{m}}^{\alpha }\sum_{\beta =1}^{M}J^{\alpha \beta }\left(\epsilon^{\alpha \beta }*A_{N}^{\beta }\right)\left(t\right). \end{array}$$ (30)

with *J*^*αβ*^ = *p*^*αβ*^ *N*^*β*^ *w*^*αβ*^. The initial condition is $u_{A}^{\alpha }\left(\hat{t},\hat{t}\right)=u_{\text{r}}$ corresponding to the reset of the membrane potential after the last spike. If we use this insight for the conditional intensity $f^{\alpha }\left(u_{i}^{\alpha }\left(t\right)-\vartheta_{i}^{\alpha }\left(t\right)\right)$ we see that the explicit dependence upon $u_{i}^{\alpha }\left(t\right)$ can be dropped as long as we keep track of the last spike time ${\hat{t}}_{i}^{\alpha }$, cf. Eq (28); hence $u_{i}^{\alpha }\left(t\right)=u_{A}^{\alpha }\left(t,{\hat{t}}_{i}^{\alpha }\right)$.

In a randomly connected network (*p*^*αβ*^ < 1), the synaptic input is different for each neuron. On the population level, however, this heterogeneity is averaged allowing us to still use the mean-field approximation, Eq (30). To see this, we note that in our network with fixed in-degree, each neuron *i* in population *α* has *p*^*αβ*^*N*^*β*^ presynaptic neurons in population *β* (*β* = *α* is possible). This means that in Eq (22) we can approximate the sum $\sum_{j\in \Gamma_{i}^{\beta }}s_{j}^{\beta }\left(t\right)$ over the *p*^*αβ*^ *N*^*β*^ presynapic neurons by

$$\begin{array}{c} p^{\alpha \beta }N^{\beta }\left(\frac{1}{p^{\alpha \beta }N^{\beta }}\sum_{j\in \Gamma_{i}^{\beta }}s_{j}^{\beta }\left(t\right)\right)\approx p^{\alpha \beta }N^{\beta }A_{N}^{\beta }\left(t\right) \end{array}$$ (31)

(cf. definition of $A_{N}^{\beta }\left(t\right)$ in Eq (1)). The mean-field approximation, Eq (31), only depends on the population activity and is thus identical for all neurons. Therefore, fluctuations of the population activity can be regarded as common input fluctuations that are coherent across neurons. On the other hand, the deviation from the mean-field approximation (i.e. the difference between the left- and right-hand side of Eq (31)) is different for each neuron and can be regarded as incoherent noise. For low connection probabilities, this incoherent part of the fluctuations may lead to a significant deviation of the membrane potential $u_{i}^{\alpha }\left(t\right)$ from the mean-field approximation $u_{A}^{\alpha }\left(t,{\hat{t}}_{i}^{\alpha }\right)$ (Fig 6A, 6C and 6E, top). On the mesoscopic scale, however, the total number of spikes in a small time step Δ*t* is determined by the population-averaged conditional firing rate, Eq (28), (cf. Eq (46) below). Hence, for sufficiently large *N*^*α*^, incoherent noise average out, whereas common finite-size fluctuations survive (Fig 6A, 6C and 6E, bottom). Note, however, that incoherent noise may cause a small bias because we average a nonlinear function of the noisy membrane potential on the l.h.s. of Eq (28). Effectively, the incoherent noise softens the threshold of the escape noise mechanism.

#### Quasi-renewal approximation

So far we have reduced the conditional intensity to $\lambda_{i}^{\alpha }\left(t\right)\approx f(u_{A}\left(t,{\hat{t}}_{i}^{\alpha }\right)-\vartheta_{i}^{\alpha }\left(t\right))$. This expression still involves the individual threshold $\vartheta_{i}^{\alpha }\left(t\right)$ of neuron *i* in population *α*, which depends on the full spike history of that neuron. This means that the spike-train is generally not a time-dependent renewal process. Here, we employ the quasi-renewal approximation [63] and average over the spikes before the last spike time assuming that they occurred according to an inhomogeneous Poisson process with rate $A_{N}^{\alpha }\left(t^{\prime }\right)$, $t^{\prime }<{\hat{t}}_{i}^{\alpha }$. Averaging the conditional intensity, Eq (25), in this way, conditioned on a given last spike time ${\hat{t}}_{i}^{\alpha }$ and a given history $A_{N}^{\alpha }\left(t^{\prime }\right)$, $t^{\prime }<{\hat{t}}_{i}^{\alpha }$, yields [63, 134, 135]

$$\begin{array}{c} \lambda_{i}^{\alpha }\left(t\right)\approx f^{\alpha }\left(u_{A}^{\alpha }\left(t,{\hat{t}}_{i}^{\alpha }\right)-\vartheta_{A}^{\alpha }\left(t,{\hat{t}}_{i}^{\alpha }\right)\right)\equiv \lambda_{A}^{\alpha }\left(t|{\hat{t}}_{i}^{\alpha }\right), \end{array}$$ (32)

where $\vartheta_{A}^{\alpha }\left(t,{\hat{t}}_{i}^{\alpha }\right)$ is an effective dynamic threshold given by

$$\begin{array}{c} \vartheta_{A}^{\alpha }\left(t,\hat{t}\right)=u_{\text{th}}+\theta^{\alpha }\left(t-\hat{t}\right)+\int_{-\infty }^{\hat{t}}{\tilde{\theta }}^{\alpha }\left(t-t^{\prime }\right)A_{N}^{\alpha }\left(t^{\prime }\right)dt^{\prime }. \end{array}$$ (33)

Here, ${\tilde{\theta }}^{\alpha }\left(t\right)=\Delta_{u}^{\alpha }[1-e^{-\theta^{\alpha }\left(t\right)/\Delta_{u}^{\alpha }}]$ is the so-called quasi-renewal kernel [63], while $\theta^{\alpha }\left(t-\hat{t}\right)$ describes the increase of the threshold induced by the last spike. Note that as a result of the two approximations, the conditional firing rate no longer depends on the precise spiking history of a given neuron and its presynaptic neurons, but only on its last firing time, cf. Eqs (25) and (32). This ends our explanation of Eq (28).

### Discretized population density equations

Using the mean-field approximation Eq (32), we have reduced the model to a population of time-dependent renewal processes [23, 68], where the conditional intensity of neuron *i* is $\lambda_{A}^{\alpha }\left(t|{\hat{t}}_{i}^{\alpha }\right)$. Neurons are effectively coupled through the dependence of $\lambda_{A}^{\alpha }\left(t|{\hat{t}}_{i}^{\alpha }\right)$ upon the membrane potential $u_{A}^{\alpha }\left(t|{\hat{t}}_{i}\right)$, which in turn depends on the activities $A_{N}^{\beta }$ of all populations *β* that are connected to population *α*. This is the only place where population labels different from *α* appear. For the sake of notational simplicity, we will omit the population label *α* and the subscript *A* in this section, keeping in mind that all quantities refer to population *α* and that the coupling with other populations is implicitly contained in $u_{A}^{\alpha }\left(t|{\hat{t}}_{i}\right)$.

#### Microscopic dynamics of the refractory density

**Fig 10:**
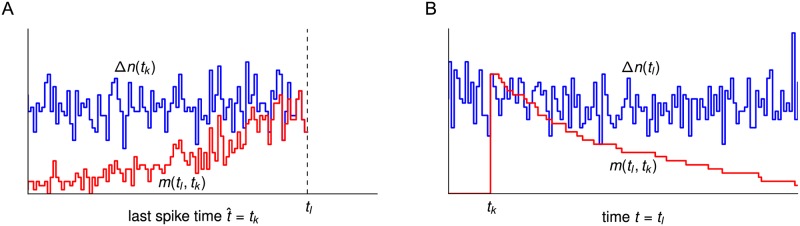
Different interpretations of the function $m(t,\hat{t})$ (red line). (A) As a function of $\hat{t}$ (or as a function of *k* in discrete time), *m*(*t*_*l*_, *t*_*k*_) represents the distribution of last spike times ${\hat{t}}_{i}$ across the population at time *t* = *t*_*l*_. (B) As a function of time *t* (or as a function of the index *l* in discrete time), *m*(*t*_*l*_, *t*_*k*_) represents the survival number, i.e. the number of neurons which fired in the interval [*t*_*k*_, *t*_*k*_ + Δ*t*) which survived (did not fire) until time *t* = *t*_*l*_. The activity Δ*n*(*t*_*k*_), i.e. the number of neurons that fired in the *k*-th time bin, is depicted by a blue line. The population size is *N* = 1000.

Because the firing probability of a neuron only depends on its last spike time and the mesoscopic population activity in the past, we can use a population density description of all last spike times ${\hat{t}}_{i}$ in the population. To derive such representation it is useful to discretize time by introducing the discrete time points *t*_*k*_ = *t*_0_ + *k*Δ*t*, k∈Z, and the corresponding intervals $I_{k}=\left[t_{k},t_{k}+\Delta t\right)$. Time is measured relative to a reference time *t*_0_, which, however, is irrelevant for the following arguments. We require that the size of the intervals Δ*t* is sufficiently small so that each neuron fires at most once during any interval. Specifically, we require that Δ*t* ≤ *t*_ref_. We also require that the sum of axonal and synaptic delays is not smaller than Δ*t*. Furthermore, we identify the discrete time point *t*_*l*_ as the current time, whereas indices *k* with *k* < *l* correspond to the past. In the population density approach, we do not keep track of the last spike time of each individual neuron but for each past time interval $I_{k}$ we only track the number of those neurons that have their last spike time in this interval. This number is denoted by *m*(*t*_*l*_, *t*_*k*_). The collection ${\left\{m\left(t_{l},t_{k}\right)\right\}}_{k\in Z,k<l}$ of these numbers for all intervals $I_{k}$, *k* < *l*, represents the current distribution of last spike times ${\hat{t}}_{i}$ in the population at time *t*_*l*_ (Fig 10A). Because each neuron has exactly one last spike time, the distribution *m*(*t*_*l*_, *t*_*k*_) is normalized to the total number of neurons:

$$\begin{array}{c} \sum_{k=-\infty }^{l-1}m\left(t_{l},t_{k}\right)=N. \end{array}$$ (34)

Since the last spike time determines the refractory state of a neuron, the distribution *m*(*t*_*l*_, *t*_*k*_) will be also called refractory distribution and the function *Q*_*N*_(*t*_*l*_, *t*_*k*_) ≡ *m*(*t*_*l*_, *t*_*k*_)/(*N*Δ*t*) can be regarded as the corresponding refractory density. The refractory distribution fully characterizes the microscopic state of the population.

We now introduce the number of neurons that fired a spike in the interval $I_{k}$ (not necessarily the last spike). This number is denoted by Δ*n*(*t*_*k*_) (Fig 10A) and is related to the population activity by *A*_*N*_(*t*_*k*_) = Δ*n*(*t*_*k*_)/(*N*Δ*t*). Therefore, Δ*n*(*t*_*k*_) will be often referred to as simply the “activity” at time *t*_*k*_. Knowing the past activities Δ*n*(*t*_*k*′_) for *k*′ < *l* and the last spike time *t*_*k*_ fully determines the membrane potentials *u*(*t*_*l*_, *t*_*k*_) and thresholds *ϑ*(*t*_*l*_, *t*_*k*_), and hence the escape rate λ(*t*_*l*_|*t*_*k*_) = *f*(*u*(*t*_*l*_, *t*_*k*_) − *ϑ*(*t*_*l*_, *t*_*k*_)) associated with the interval $I_{k}$. Thus, the knowledge of the past activities and the distribution of last spike times at time *t*_*l*_ is sufficient to statistically determine these quantities at time *t*_*l*+1_. In other words, the evolution of the system can be described by a Markov process if we define the microscopic state $X(t_{l})$ of the population at time *t*_*l*_ by the sequence of pairs

$$\begin{array}{c} X\left(t_{l}\right)={\left\{\left(\Delta n\left(t_{k}\right),m\left(t_{l},t_{k}\right)\right)\right\}}_{k\in Z,k<l}\;. \end{array}$$ (35)

In the following, the main task will be to derive the statistics of the number of spikes Δ*n*(*t*_*l*_) in the next time interval [*t*_*l*_, *t*_*l*_ + Δ*t*) and the distribution *m*(*t*_*l*_ + Δ*t*, *t*_*k*_) of last spike times at time *t*_*l*+1_ given the state $X(t_{l})$ at time *t*_*l*_. We mention that what we have lost in this population density description is only the information about the identity of the neurons, which, however, is irrelevant for the mesoscopic description of homogeneous populations.

There is a second interpretation of *m*(*t*_*l*_, *t*_*k*_): let us consider the group of neurons that have fired in the interval $I_{k}$, *k* < *l*. The number of neurons from this group that have “survived” (i.e. that have not fired again) until time *t*_*l*_ is exactly given by *m*(*t*_*l*_, *t*_*k*_). We will therefore also call it number of survived neurons or survival number for the refractory state *k*. Correspondingly, the ratio *S*_*N*_(*t*_*l*_|*t*_*k*_) = *m*(*t*_*l*_, *t*_*k*_)/Δ*n*(*t*_*k*_) is the fraction of survived neurons. As time *t*_*l*_ evolves, the number of survived neurons diminishes whenever there is a spike in that group (Fig 10B). Thus, if the group fires *X*_*lk*_ spikes in the time step [*t*_*l*_, *t*_*l*_ + Δ*t*), then *m*(*t*_*l*_, *t*_*k*_) decreases by *X*_*lk*_. For *l* > *k*, this gives rise to the evolution equation

$$\begin{array}{c} m(t_{l}+\Delta t,t_{k})=m\left(t_{l},t_{k}\right)-X_{lk}. \end{array}$$ (36)

The initial condition is given by *m*(*t*_*k*_ + Δ*t*, *t*_*k*_) = Δ*n*(*t*_*k*_), which follows from the absolute refractoriness during the first time step after a spike. Absolute refractoriness also entails that each neuron can fire only one spike per time step (Δ*t* ≤ *t*_ref_) with a firing probability

$$\begin{array}{c} P_{\lambda }\left(t_{l}|t_{k}\right)=1-\exp\left(-\int_{t_{l}}^{t_{l}+\Delta t}\lambda \left(s|t_{k}\right)\;ds\right)\approx 1-e^{-\bar{\lambda }\left(t_{l}|t_{k}\right)\Delta t}. \end{array}$$ (37)

In the last step, we introduced the average hazard rate $\bar{\lambda }\left(t_{l}|t_{k}\right)=\left[\lambda \left(t_{l}|t_{k}\right)+\lambda \left(t_{l+1}|t_{k}\right)\right]/2$. Because the past activities Δ*n*(*t*_*k*_), *k* < *l*, completely determine the probability to fire *P*_λ_(*t*_*l*_|*t*_*k*_), each neuron decides independently from the others whether it fires in the next time step. Furthermore, there is a total number of *m*(*t*_*l*_, *t*_*k*_) neurons from the considered group that could potentially fire in the interval [*t*_*l*_, *t*_*l*_ + Δ*t*). Therefore, the number of spikes *X*_*lk*_ is the result of *m*(*t*_*l*_, *t*_*k*_) independent Bernoulli trials with success probability *P*_λ_(*t*_*l*_|*t*_*k*_). This implies that *X*_*lk*_ follows a binomial distribution:

$$\begin{array}{c} X_{lk}∼B\left(m\left(t_{l},t_{k}\right),P_{\lambda }\left(t_{l}|t_{k}\right)\right). \end{array}$$ (38)

Moreover, the random numbers *X*_*lk*_ associated with different past time intervals $I_{k}$ are conditionally independent given the current state of the system $X(t_{l})$ (cf. Eq (35)).

The total number of spikes emitted in the current interval [*t*_*l*_, *t*_*l*_+Δ*t*) is equal to the total reduction of survivals in that interval, hence

$$\begin{array}{c} \Delta n\left(t_{l}\right)=\sum_{k=-\infty }^{l-1}X_{lk}. \end{array}$$ (39)

Eqs (36)–(39) define the microscopic kinetics in discrete time. In a simulation, for each past time interval $I_{k}$ one independent Poisson random number *X*_*lk*_ needs to be drawn per time step and population. These random numbers determine the current spike count via Eq (39) and the update of the distribution of last spike times *m*(*t*_*l*_, *t*_*k*_), via Eq (36). We call this description microscopic because for small time steps, there will be many (order of *N*) intervals $I_{k}$ that contain survived neurons, i.e. for which *m*(*t*_*l*_, *t*_*k*_) > 0 and for each of which one needs to draw a random number *X*_*lk*_ in a simulation. In the limit Δ*t* → 0, such a simulation would be as complex as the original microscopic simulation of *N* neurons.

The microscopic population density description can be summarized in a particularly compact form by performing the continuum limit Δ*t* → 0 and by assuming large *N*. For large *N*, the statistics of *X*(*t*_*l*_, *t*_*k*_) becomes Gaussian with mean and variance *P*_λ_(*t*_*l*_|*t*_*k*_)*m*(*t*_*l*_, *t*_*k*_). Thus, the dynamics of $m(t,\hat{t})$, Eq (36), can be rewritten as

$$\begin{array}{c} m\left(t_{l}+\Delta t,t_{k}\right)-m\left(t_{l},t_{k}\right)=-P_{\lambda }\left(t_{l}|t_{k}\right)m\left(t_{l},t_{k}\right)+\sqrt{P_{\lambda }\left(t_{l}|t_{k}\right){\left[m\left(t_{l},t_{k}\right)\right]}_{+}}N\left(t_{l},t_{k}\right), \end{array}$$ (40)

where ${\left\{N\left(t_{l},t_{k}\right)\right\}}_{k,l\in Z}$ are independent, standard normal random numbers and the ramp function [*x*]_+_ = *x*Θ(*x*) ensures non-negativity of *m*. Using the density of last spike times *Q*_*N*_(*t*_*l*_, *t*_*k*_) ≡ *S*_*N*_(*t*_*l*_|*t*_*k*_)*A*_*N*_(*t*_*k*_) ≡ *m*(*t*_*l*_, *t*_*k*_)/(*N*Δ*t*), setting *t*_*l*_ = *t* and $t_{k}=\hat{t}$, and expanding $P_{\lambda }\left(t|\hat{t}\right)\approx \lambda \left(t|\hat{t}\right)\Delta t$ for small Δ*t* we arrive at the following dynamics in the limit Δ*t* → 0:

$$\begin{array}{c} \frac{\partial Q_{N}\left(t,\hat{t}\right)}{\partial t}=-\lambda \left(t|\hat{t}\right)Q_{N}\left(t,\hat{t}\right)+\sqrt{\frac{\lambda \left(t|\hat{t}\right){\left[Q_{N}\left(t,\hat{t}\right)\right]}_{+}}{N}}\xi \left(t,\hat{t}\right). \end{array}$$ (41)

Here, $\xi \left(t,\hat{t}\right)\equiv \lim_{\Delta t\to 0}\;N\left(t,\hat{t}\right)/\Delta t$ is a Gaussian random field with zero mean and correlation function $\langle \xi \left(t,\hat{t}\right)\xi \left(t^{\prime },{\hat{t}}^{\prime }\right)\rangle =\delta \left(t-t^{\prime }\right)\delta \left(\hat{t}-{\hat{t}}^{\prime }\right)$. For a given last spike time $\hat{t}$, Eq (41) has the form of a Langevin equation. Its initial condition is $Q_{N}\left(\hat{t},\hat{t}\right)=A_{N}\left(\hat{t}\right)$. The population activity results from Eq (39) as the integral of changes of the refractory density $Q_{N}\left(t,\hat{t}\right)$ over all refractory states, i.e. $A_{N}\left(t\right)=-\int_{-\infty }^{t}\partial_{t}Q_{N}\left(t,\hat{t}\right)\;d\hat{t}$, or using Eq (41):

$$\begin{array}{c} A_{N}\left(t\right)=\int_{-\infty }^{t}\lambda \left(t|\hat{t}\right)Q_{N}\left(t,\hat{t}\right)\;d\hat{t}-\int_{-\infty }^{t}\sqrt{\frac{\lambda \left(t|\hat{t}\right){\left[Q_{N}\left(t,\hat{t}\right)\right]}_{+}}{N}}\xi \left(t,\hat{t}\right)\;d\hat{t}. \end{array}$$ (42)

Eqs (41) and (42) represent the microscopic population density equations in continuous time under the Gaussian and quasi-renewal approximations.

#### Mesoscopic description

At the mesoscopic level, we want to describe the state of the population at time *t*_*l*_ only by the mesoscopic variables Δ*n*(*t*_*k*_), *k* < *l*, that have been observed so far. Therefore, we define the history of *n* at time *t*_*l*_ by

$$\begin{array}{c} H\left(t_{l}\right)={\left\{\Delta n(t_{k})\right\}}_{k\in Z,k<l}, \end{array}$$ (43)

which completely determines the mesoscopic state. In contrast to the microscopic state $X(t_{l})$ defined in Eq (35), the mesoscopic state does not require the knowledge of the detailed distribution of last spike times *m*(*t*_*l*_, *t*_*k*_). We call a variable mesoscopic if it only depends on the history $H(t_{l})$. Likewise, an equation is called mesoscopic if it only involves mesoscopic variables. In the following sections, all averages at a given time *t*_*l*_ have to be understood as conditional averages given the history $H(t_{l})$. We will therefore often omit an explicit notation of this condition.

To derive a mesoscopic equation, we want to find an approximate dynamics with only one effective, mesoscopic noise term that summarizes the effect of all microscopic random variables *X*_*lk*_. In a simulation, this would imply to draw only one random number per time step and per population. Towards that end, we assume that Δ*t* can be chosen sufficiently small such that *P*_λ_(*t*_*l*_|*t*_*k*_) ≪ 1, which is always possible if neurons are stochastic und hence do not perfectly synchronize. Under this assumption, the binomially-distributed random numbers *X*_*lk*_ are approximately Poisson-distributed, i.e.

$$\begin{array}{c} X_{lk}∼\text{Pois}\left(\text{E}\left[X_{lk}|m\left(t_{l},t_{k}\right)\right]\right), \end{array}$$ (44)

where

$$\begin{array}{c} \text{E}\left[X_{lk}|m\left(t_{l}|t_{k}\right)\right]=P_{\lambda }\left(t_{l}|t_{k}\right)m\left(t_{l},t_{k}\right). \end{array}$$ (45)

is the conditional mean of *X*_*lk*_ given the current survival number *m*(*t*_*l*_|*t*_*k*_). Given the conditional independence of *X*_*lk*_ for different *k*, the Poisson property implies that the global activity Δ*n*(*t*_*l*_) in Eq (39) is also Poisson-distributed given the current refractory distribution {*m*(*t*_*l*_, *t*_*k*_)}_*k*<*l*_, i.e.

$$\begin{array}{c} \Delta n\left(t_{l}\right)∼\text{Pois}\left(\Delta \bar{n}\left(t_{l}\right)\right), \end{array}$$ (46)

with mean

$$\begin{array}{c} \Delta \bar{n}\left(t_{l}\right)\equiv \text{E}\left[\Delta n\left(t_{l}\right)|{\left\{m\left(t_{l},t_{k}\right)\right\}}_{k<l},H\left(t_{l}\right)\right]=\sum_{k=1}^{\infty }P_{\lambda }\left(t_{l}|t_{k}\right)m\left(t_{l},t_{k}\right). \end{array}$$ (47)

Because of the definition of refractory densities and *P*_λ_ ≤ 1, we find that $\Delta \bar{n}\left(t_{l}\right)\leq N$ is automatically satisfied at any moment in time. However, for the numerical implementation with finite Δ*t* later on we need to keep in mind that the Poisson number Δ*n*(*t*) could become larger than *N*, if $\Delta \bar{n}\left(t_{l}\right)$ is close to *N*. In this case, using a binomial statistics will be more appropriate, as explained in Sec. “Numerical implementation”.

Eqs (46) and (47) suggest the possibility to generate Δ*n*(*t*_*l*_) by a *single* Poisson-distributed random number. However, Eq (47) is not a mesoscopic equation yet because it still depends on the dynamics of *m*(*t*_*l*_, *t*_*k*_), Eq (36), which contains the microscopic random variables *X*_*lk*_. There is another, more subtle problem if we want to use Eqs (46) and (47) as a mesoscopic dynamics that generates the activities Δ*n*(*t*_*l*_). If we regard Δ*n*(*t*_*l*_) as an *independent* random variable, the conservation of neurons, Eq (39), imposes a constraint on the microscopic random numbers {*X*_*lk*_}_*k*<*l*_, which will therefore not be independent anymore. Conversely, if we consider both {*X*_*lk*_}_*k*<*l*_ and Δ*n*(*t*_*l*_) as independent variables, we almost certainly violate the conservation of neurons, Eq (39), or equivalently, the normalization condition Eq (34). This problem does not occur in the microscopic dynamics, where Δ*n*(*t*_*l*_) is a *dependent* variable generated from the independent random variables {*X*_*lk*_}_*k*<*l*_ via Eq (39), and hence the correct normalization is guaranteed at any time. Nevertheless, the “non-normalized” or “unconstrained” process, in which {*X*_*lk*_}_*k*<*l*_ and Δ*n*(*t*_*l*_) are drawn independently, will be useful for deriving mesoscopic equations because it allows us to calculate the moments of the survival numbers *m*(*t*_*l*_, *t*_*k*_). Our main strategy is to use these moments in conjunction with the normalization condition to express the expected spike count $\Delta \bar{n}\left(t_{l}\right)$, Eq (47), as a deterministic functional of the past activities. In this way, $\Delta \bar{n}\left(t_{l}\right)$ will not depend anymore on the actual microscopic realizations of the constrained noise ${\left\{X_{l^{\prime },k}\right\}}_{k,l^{\prime }\in Z,k<l^{\prime }<l}$ (constrained by a given history {Δ*n*(*t*_*k*_)}_*k*<*l*_ via Eq (39)) and can thus be used to generate Δ*n*(*t*_*l*_) as a Poisson random number from the knowledge of the past activities.

#### Moment equations

To achieve such deterministic relationship, we first derive mesoscopic equations for the mean and variance of *m*(*t*_*l*_, *t*_*k*_) given the history $H(t_{l})$ in the so-called *non-normalized ensemble* or *unconstrained* ensemble. This means that the history determines the initial conditions of the dynamics of *m*(*t*_*l*_, *t*_*k*_), Eq (36), as well as the conditional intensities λ(*t*_*l*_|*t*_*k*_), but it does not impose the constraint Eq (39) on the random numbers {*X*_*l*′, *k*_}_*l*′≤*l*_. Although this unconstrained noise leads to a non-normalized distribution $\hat{m}\left(t_{l},t_{k}\right)$, it still yields a very good approximation of its mean and variance in the actual constrained ensemble. Taking the average of Eq (36), and using Eq (44) yields the evolution of the mean:

$$\begin{array}{c} \left\langle {\hat{m}}_{l+1,k}\right\rangle =\left[1-P_{\lambda }\left(t_{l}|t_{k}\right)\right]\left\langle {\hat{m}}_{l,k}\right\rangle \end{array}$$ (48)

with initial condition $\langle {\hat{m}}_{k+1,k}\rangle =\Delta n\left(t_{k}\right)$. Here and in the following, ${\hat{m}}_{l,k}$ is short-hand for $\hat{m}\left(t_{l},t_{k}\right)$ to simplify the notation, and 〈⋅〉 denotes the ensemble average of the unconstrained process for a given history $H(t_{l})$. Actually, the condition for the average 〈⋅〉 can be extended to the history $H(t_{l+1})$ (and to any future activities) because in the unconstrained ensemble neither ${\hat{m}}_{l,k}$ nor ${\hat{m}}_{l+1,k}$ depend on the most recent activity Δ*n*(*t*_*l*_) (clearly, this also holds for any future activity). Importantly, Eq (48) is a mesoscopic equation because it is fully determined by the past activities.

As a next step we derive an equation for the variance of $\hat{m}$. To this end, let

$$\begin{array}{c} \Delta {\hat{m}}_{l,k}={\hat{m}}_{l,k}-\left\langle {\hat{m}}_{l,k}\right\rangle \end{array}$$ (49)

denote the deviation from the mean. Using the law of total variance, we find for the variance in the next time step

$$\begin{array}{c} \left\langle \Delta {\hat{m}}_{l+1,k}^{2}\right\rangle =\text{Var}\left[E\left[m_{l+1,k}|m_{l,k},H_{l}\right]\right]+\left\langle \text{Var}\left[m_{l+1,k}|m_{l,k},H_{l}\right]\right\rangle . \end{array}$$ (50)

The conditional mean of *m*_*l*+1,*k*_ given the current value *m*_*l*,*k*_, denoted by *E*[*m*_*l*+1,*k*_|*m*_*l*,*k*_], follows from the evolution Eqs (36) and (45) as [1 − *P*_λ_(*t*_*l*_|*t*_*k*_)]*m*_*l*,*k*_. Therefore, its variance is ${\left[1-P_{\lambda }\left(t_{l}|t_{k}\right)\right]}^{2}\langle \Delta m_{l,k}^{2}\rangle$. For the second term in Eq (50), we note that the conditional variance Var[*m*_*l*+1,*k*_|*m*_*l*, *k*_] is equal to the variance Var[*X*_*lk*_|*m*_*l*, *k*_] of the decrement *X*_*lk*_. Because *X*_*lk*_ is a Poisson variable, this variance is equal to the mean given by Eq (45). Taken together, we find the following update rule for the total variance

$$\begin{array}{c} \left\langle \Delta {\hat{m}}_{l+1,k}^{2}\right\rangle ={\left[1-P_{\lambda }\left(t_{l}|t_{k}\right)\right]}^{2}\left\langle \Delta {\hat{m}}_{l,k}^{2}\right\rangle +P_{\lambda }\left(t_{l}|t_{k}\right)\left\langle {\hat{m}}_{l,k}\right\rangle \end{array}$$ (51)

with initial condition $\langle \Delta {\hat{m}}_{k+1,k}^{2}\rangle =0$. As a function of *t*_*l*_ (Fig 2C bottom), the variance $\langle \Delta {\hat{m}}^{2}\left(t_{l},t_{k}\right)\rangle$ is initially zero because all neurons have still survived immediately after firing at time *t*_*k*_. On the other hand, at long times *t*_*l*_ ≫ *t*_*k*_, the variance also vanishes because according to Eq (48), the mean number of survived neurons 〈*m*(*t*_*l*_, *t*_*k*_)〉 appearing in Eq (51) goes to zero. As a consequence, the variance obtains a maximum at an intermediate time. Similarly, the dependence of the variance at time *t*_*l*_ for different last spike times $\hat{t}=t_{k}$ shows the same limiting behavior which implies a maximum at an intermediate last spike time $\hat{t}$ (Fig 2B bottom). However, the rugged shape of this function with many local maxima reflects the discontinuity of the driving force 〈*m*(*t*_*l*_, *t*_*k*_)〉 as a function of *t*_*k*_ that arises from the stochastic initial condition 〈*m*(*t*_*k*+1_, *t*_*k*_)〉 = Δ*n*(*t*_*k*_).

#### Mesoscopic population equations

Let us return to Eq (47) for the expected activity $\Delta \bar{n}\left(t_{l}\right)$, which is used to draw the activity Δ*n*(*t*_*l*_) as a Poisson random number (cf. Eq (46)). Because we condition on the history {Δ*n*(*t*_*l*′_)}_*l*′<*l*_, the processes *m*_*l*,*k*_ in this equation belong to the “constrained” ensemble, in which the normalization condition, Eq (34), is obeyed. We note that these constrained processes could in principle be generated microscopically by Eq (36) if at each time *t*_*l*′_ in the past, the microscopic noise *X*_*l*′*k*_ was sampled from a joint distribution that ensures the conservation of neurons, Eq (39), i.e. ∑_*k*_ *X*_*l*′*k*_ = Δ*n*(*t*_*l*′_). However, as we will show in the following, such a construction is not needed because the dependence of the expected activity $\Delta \bar{n}\left(t_{l}\right)$ on a specific realization of *m*_*l*,*k*_ can be eliminated by exploiting the normalization condition, Eq (34). To this end, we take advantage of the fact that the mean $\langle {\hat{m}}_{l,k}\rangle$ of the unconstrained process is a mesoscopic variable. This suggests to split the constrained processes *m*_*l*,*k*_ into the mean of the unconstrained process and a fluctuation part:

$$\begin{array}{c} m_{l,k}=\left\langle {\hat{m}}_{l,k}\right\rangle +\delta m_{l,k}. \end{array}$$ (52)

The first contribution is deterministic given the past activities Δ*n*(*t*_*k*_) while the second contribution represents the microscopic fluctuations. We note that the fluctuation *δm*_*l*,*k*_ is not equivalent to the deviation $\Delta {\hat{m}}_{l,k}$ of the unconstrained process because $\langle {\hat{m}}_{l,k}\rangle +\Delta {\hat{m}}_{l,k}$ does not obey the normalization condition, Eq (34), whereas $\langle {\hat{m}}_{l,k}\rangle +\delta m_{l,k}$ does.

To remove the microscopic fluctuations *δm*_*l*,*k*_, we require that both Eqs (34) and (47) are simultaneously satisfied. Substituting Eq (52) into these equations leads to

$$\begin{array}{c} N=\sum_{k=-\infty }^{l-1}\left\langle {\hat{m}}_{l,k}\right\rangle +\sum_{k=-\infty }^{l-1}\delta m_{l,k}, \end{array}$$ (53)

$$\begin{array}{c} \Delta \bar{n}\left(t_{l}\right)=\sum_{k=-\infty }^{l-1}P_{\lambda }\left(t_{l}|t_{k}\right)\left\langle {\hat{m}}_{l,k}\right\rangle +\sum_{k=-\infty }^{l-1}P_{\lambda }\left(t_{l}|t_{k}\right)\delta m_{l,k}. \end{array}$$ (54)

The microscopic fluctuations *δm*_*l*,*k*_ enter the dynamics only in the form of two sums. First, the normalization condition Eq (53) imposes a strict relation between the summed deviation ∑_*k*_ *δm*_*l*,*k*_ and the means of the unconstrained processes, $\langle {\hat{m}}_{l,k}\rangle$, irrespective of the specific, underlying microscopic dynamics of *m*_*l*,*k*_. In particular, we can solve for $\sum_{k}\;\delta m_{l,k}=N-\sum_{k}\langle {\hat{m}}_{l,k}\rangle$ with terms on the r.h.s. that are completely determined given the past activities. Second, the total effect of the deviations on the expected activity $\Delta \bar{n}\left(t_{l}\right)$ is given by the weighted sum ∑_*k*_ *P*_λ_(*t*_*l*_|*t*_*k*_)*δm*_*l*,*k*_ in Eq (54) with *P*_λ_(*t*_*l*_|*t*_*k*_) ≤ 1 for all *k* < *l*. The weighted sum ∑_*k*_ *P*_λ_(*t*_*l*_|*t*_*k*_)*δm*_*l*,*k*_ is therefore tightly constrained by the value of the summed fluctuation ∑_*k*_ *δm*_*l*,*k*_. These considerations suggest to make the following decoupling approximation:

$$\begin{array}{c} \sum_{k=-\infty }^{l-1}P_{\lambda }\left(t_{l}|t_{k}\right)\delta m_{l,k}\approx P_{\Lambda }\left(t_{l}\right)\sum_{k=-\infty }^{l-1}\delta m_{l,k} \end{array}$$ (55)

with a still unknown factor *P*_Λ_(*t*_*l*_), that we call effective firing probability. To determine the effective firing probability, we require that in the unconstrained ensemble the corresponding approximation

$$\begin{array}{c} \sum_{k=-\infty }^{l-1}P_{\lambda }\left(t_{l}|t_{k}\right)\Delta {\hat{m}}_{l,k}=P_{\Lambda }\left(t_{l}\right)\sum_{k=-\infty }^{l-1}\Delta {\hat{m}}_{l,k}+\varepsilon_{l} \end{array}$$ (56)

minimizes the mean squared error $E\left(P_{\Lambda }\right)=\langle \varepsilon_{l}^{2}\rangle$, where *P*_Λ_ is short-hand for *P*_Λ_(*t*_*l*_). We use the unconstrained deviations $\Delta {\hat{m}}_{lk}$ here because we are only interested in the typical error. Note that if *ε*_*l*_ is Gaussian distributed, minimizing the mean squared error yields the optimal estimation of *P*_Λ_ in the sense that it maximizes the log-likelihood of $\sum_{k}\;P_{\lambda }\left(t_{l}|t_{k}\right)\Delta {\hat{m}}_{l,k}$ given $\sum_{k}\;\Delta {\hat{m}}_{l,k}$ under the linear approximation Eq (56). The error can be rewritten as *ε*_*l*_ = ∑_*k*_[*P*_λ_(*t*_*l*_|*t*_*k*_) − *P*_Λ_(*t*_*l*_)]Δ*m*_*l*, *k*_, which for *N* ≫ 1 is a sum of many independent variables that can indeed be considered to be Gaussian. The derivative of E with respect to *P*_Λ_ is

$$\begin{array}{c} \frac{dE}{dP_{\Lambda }}=2P_{\Lambda }\sum_{k,k^{\prime }<l}\left\langle \Delta {\hat{m}}_{l,k}\Delta {\hat{m}}_{l,k^{\prime }}\right\rangle -2\sum_{k,k^{\prime }<l}P_{\lambda }\left(t_{l}|t_{k}\right)\left\langle \Delta {\hat{m}}_{l,k}\Delta {\hat{m}}_{l,k^{\prime }}\right\rangle , \end{array}$$ (57)

where we have exploited that *P*_λ_(*t*_*l*_|*t*_*k*_) is deterministic given the past activities Δ*n*(*t*_*k*_), *k* < *l*. Furthermore, under this condition, the fluctuations $\Delta {\hat{m}}_{l,k}$ and $\Delta {\hat{m}}_{l,k^{\prime }}$ with *k* ≠ *k*′ are conditionally independent. Using this property and setting $dE/dP_{\Lambda }=0$ we find that the optimal effective firing probability is

$$\begin{array}{c} P_{\Lambda }\left(t_{l}\right)=\frac{\sum_{k=-\infty }^{l-1}P_{\lambda }\left(t_{l}|t_{k}\right)\left\langle \Delta {\hat{m}}_{l,k}^{2}\right\rangle }{\sum_{k=-\infty }^{l-1}\left\langle \Delta {\hat{m}}_{l,k}^{2}\right\rangle }. \end{array}$$ (58)

The variance $\langle \Delta {\hat{m}}_{l,k}^{2}\rangle$ in this formula obeys the mesoscopic dynamics derived above in Eq (51). Hence, the effective firing probability is itself mesoscopic.

Using Eqs (53) and (55), ∑_*k*_ *δm*_*l*, *k*_ can be eliminated in Eq (54) resulting in

$$\begin{array}{c} \Delta \bar{n}\left(t_{l}\right)=\sum_{k=-\infty }^{l-1}P_{\lambda }\left(t_{l}|t_{k}\right)\left\langle m_{l,k}\right\rangle +P_{\Lambda }\left(t_{l}\right)\left(N-\sum_{k=-\infty }^{l-1}\left\langle {\hat{m}}_{l,k}\right\rangle \right). \end{array}$$ (59)

Thus, we obtain an equation that yields the mean spike count $\Delta \bar{n}\left(t_{l}\right)$ at the present time as a function of the past spike counts {Δ*n*(*t*_*k*_)}_*k*<*l*_. Eq (59) is the desired mesoscopic equation in discrete time. For sufficiently small time steps Δ*t*, the present spike count Δ*n*(*t*_*l*_) can be generated by drawing a Poisson random number with mean $\Delta \bar{n}\left(t_{l}\right)$ according to Eq (46).

### Mesoscopic population density equations in continuous time

In continuous time, we consider the rescaled variables

$$\begin{array}{c} A_{N}\left(t_{l}\right)=\frac{\Delta n(t_{l})}{N\Delta t},\;\bar{A}\left(t_{l}\right)=\frac{\Delta \bar{n}\left(t_{l}\right)}{N\Delta t}. \end{array}$$ (60)

Here, $\bar{A}\left(t\right)$ can be interpreted as the *expected population activity* given the past activity *A*_*N*_(*t*′), *t*′ < *t*. For Δ*t* small but positive, the spike count Δ*n*(*t*) is an independent Poisson number with mean $\Delta \bar{n}\left(t\right)=N\bar{A}\left(t\right)\Delta t$. Thus, on a coarse-grained time scale, the continuum limit of the population activity may be written in the following suggestive way

$$\begin{array}{c} A_{N}\left(t\right)=\frac{dn(t)}{Ndt},\;dn\left(t\right)∼\text{Pois}\left(N\bar{A}\left(t\right)dt\right), \end{array}$$ (61)

where d*t* denotes an infinitesimal (but temporally coarse-grained) time step and d*n*(*t*) is an independent Poisson-distributed random number with mean $N\bar{A}\left(t\right)dt$. In the limit d*t* → 0, the spike count in an infinitesimal time step is a Bernoulli random number, where d*n*(*t*) = 1 with probability $N\bar{A}\left(t\right)dt$ and *n*(*t*) = 0 with probability $1-N\bar{A}\left(t\right)dt$. Therefore, in this limit the population activity *A*_*N*_(*t*) converges to a sequence of Dirac *δ*-functions occurring at random times *t*_pop,*k*_ with rate $N\bar{A}\left(t\right)$. Thus, *A*_*N*_(*t*) can be written more formally as a population spike train or “shot-noise”

$$\begin{array}{c} A_{N}\left(t\right)=\frac{1}{N}\sum_{k}\delta \left(t-t_{\text{pop},k}\right), \end{array}$$ (62)

where ${\left(t_{\text{pop},k}\right)}_{k\in Z}$ is a point process with conditional intensity $\lambda_{\text{pop}}\left(t|H_{t}\right)=N\bar{A}\left(t\right)$. Here, the condition $H_{t}$ denotes the history of the population activity {*A*_*N*_(*t*′)}_*t*′<*t*_, or equivalently, the history of spike times {*t*_pop,*k*_}_*t*_pop,*k*_<*t*_, up to (but not including) time *t*.

To obtain the dynamics for $\bar{A}\left(t\right)$, we also introduce the rescaled variables

$$\begin{array}{c} S\left(t_{l}|t_{k}\right)=\frac{\left\langle {\hat{m}}_{l,k}\right\rangle }{\Delta n(t_{k})},\;v\left(t_{l},t_{k}\right)=\frac{\left\langle \Delta {\hat{m}}_{l,k}^{2}\right\rangle }{N\Delta t}. \end{array}$$ (63)

The function $S(t|\hat{t})$ can be interpreted as the survival probability of neurons that have fired their last spike at time $\hat{t}$. Furthermore, for small Δ*t* the firing probability is given by *P*_λ_(*t*_*l*_|*t*_*k*_) = λ(*t*_*l*_|*t*_*k*_)Δ*t* + *O*(Δ*t*^2^). Thus, the continuum limit of Eq (59) reads

$$\begin{array}{cc} \bar{A}\left(t\right) & =\underset{\Delta t\to 0}{\text{lim}}\{\sum_{k=-\infty }^{\frac{t}{\Delta t}-1}\lambda (t\left|k\Delta t)S(t\right|k\Delta t)\frac{\Delta n\left(k\Delta t\right)}{N}\\ & \;+\frac{\sum_{k=-\infty }^{\frac{t}{\Delta t}-1}\lambda (t|k\Delta t)v\left(t,k\Delta t\right)\Delta t}{\sum_{k=-\infty }^{\frac{t}{\Delta t}-1}v\left(t,k\Delta t\right)\Delta t}\left(1-\sum_{k=-\infty }^{\frac{t}{\Delta t}-1}S(t|k\Delta t)\frac{\Delta n\left(k\Delta t\right)}{N}\right)\}. \end{array}$$ (64)

The sums in this equation can be regarded as the definition of stochastic integrals, which allows us to rewrite Eq (64) as

$$\begin{array}{c} \bar{A}\left(t\right)=\int_{-\infty }^{t}\lambda \left(t|\hat{t}\right)S\left(t|\hat{t}\right)A_{N}\left(\hat{t}\right)\;d\hat{t}+\Lambda \left(t\right)\left(1-\int_{-\infty }^{t}S\left(t|\hat{t}\right)A_{N}\left(\hat{t}\right)\;d\hat{t}\right). \end{array}$$ (65)

Here,

$$\begin{array}{c} \Lambda \left(t\right)=\frac{\int_{-\infty }^{t}\lambda \left(t|\hat{t}\right)v\left(t,\hat{t}\right)\;d\hat{t}}{\int_{-\infty }^{t}v\left(t,\hat{t}\right)\;d\hat{t}} \end{array}$$ (66)

is an effective rate corresponding to the effective firing probability *P*_Λ_(*t*). Note that according to Eq (64), the stochastic integrals in Eq (65) extend only over last spike times $\hat{t}<t$ not including time $\hat{t}=t$. Taking the continuum limit of Eq (48) we find that the survival probability satisfies the differential equation

$$\begin{array}{c} \frac{\partial S(t|\hat{t})}{\partial t}=-\lambda \left(t|\hat{t}\right)S\left(t|\hat{t}\right),\;S\left(\hat{t}|\hat{t}\right)=1. \end{array}$$ (67)

This equation has the simple solution

$$\begin{array}{c} S\left(t|\hat{t}\right)=\exp\left(-\int_{\hat{t}}^{t}\lambda \left(t^{\prime }|\hat{t}\right)\;dt^{\prime }\right). \end{array}$$ (68)

Similarly, we find from Eq (51) that the rescaled variance obeys the differential equation

$$\begin{array}{c} \frac{\partial v}{\partial t}=-2\lambda \left(t|\hat{t}\right)v+\lambda \left(t|\hat{t}\right)S\left(t|\hat{t}\right)A_{N}\left(\hat{t}\right),\;v\left(\hat{t}|\hat{t}\right)=0. \end{array}$$ (69)

The set of coupled Eqs (62)–(69) defines the mesoscopic population dynamics. We emphasize that not only *A*_*N*_(*t*) depends on $\bar{A}\left(t\right)$ (cf. Eq (62)) but that there is also a feedback of *A*_*N*_(*t*) onto the dynamics of $\bar{A}\left(t\right)$, cf. Eq (65). In fact, $\bar{A}\left(t\right)$ can be regarded as a deterministic functional of the past activities up to but not including time *t*. In particular, *A*_*N*_(*t*) is *not* an inhomogeneous Poisson spike train because the specific realization of the spike history of *A*_*N*_(*t*) determines the conditional intensity function for the point process (*t*_pop,*k*_) via Eq (65). Furthermore, we note that, in the case of synaptic coupling or adaptation, also the variables *S* and *v* depend on the history of the population activity through the dependence of $\lambda (t|\hat{t})$ on the membrane potential $u(t,\hat{t})$ and the threshold $\vartheta (t,\hat{t})$ (cf. Eqs (30) and (33)).

For large *N*, the population activity can be approximated by a Gaussian process. To this end, we note that in the discrete time description the spike counts Δ*n*(*t*_*l*_) are conditionally independent random numbers with mean and variance $N\bar{A}\left(t_{l}\right)\Delta t$. Therefore, in the large-*N* limit, the variable

$$\begin{array}{c} \Delta W\left(t_{l}\right)=\frac{\Delta n\left(t_{l}\right)-N\bar{A}\left(t_{l}\right)\Delta t}{\sqrt{N\bar{A}\left(t_{l}\right)}} \end{array}$$ (70)

is normally distributed with mean zero and variance Δ*t*, and hence corresponds to the increment of a Wiener process. Using Eq (60) for the population activity and taking the continuum limit Δ*t* → 0, we obtain

$$\begin{array}{c} A_{N}\left(t\right)=\bar{A}\left(t\right)+\sqrt{\frac{\bar{A}\left(t\right)}{N}}\xi \left(t\right), \end{array}$$ (71)

where *ξ*(*t*) = lim_Δ*t*→0_ Δ*W*(*t*)/Δ*t* is a Gaussian white noise with correlation function 〈*ξ*(*t*)*ξ*(*t*′)〉 = *δ*(*t* − *t*′). This Gaussian approximation has the advantage that the multiplicative character of the noise in Eq (71) becomes explicit because *ξ*(*t*) is independent of the state of the system. It also explicitly reveals that the finite-size fluctuations scale like $1/\sqrt{N}$. We stress again that *A*_*N*_(*t*) is not a white-noise process with time-dependent mean, as Eq (71) might suggest at first glance, but it is a sum of two mutually correlated processes, (i) a white-noise term proportional to *ξ*(*t*) that reflects the fact that the population activity is a *δ*-spike train and (ii) a colored “noise” $\bar{A}\left(t\right)$ that arises from the filtering of *ξ*(*t*) through the dynamics in Eq (65). As a result, the auto-correlation function of *A*_*N*_(*t*) contains a *δ*-peak and a continuous part, consistent with previous theoretical findings [55]. In particular, at short lags the auto-correlation function may be negative as a result of refractoriness: in this case, *ξ* and $\bar{A}$ are anti-correlated in line with the intuitive picture discussed in the Results section, Fig 2, that a positive fluctuation *ξ*(*t*) is associated with the creation of a “hole” in the distribution of last spike times leading to a reduced activity after time *t*. In the frequency domain, refractoriness corresponds to a trough in the power spectrum at low frequencies [94] as visible, for instance, in Fig 3. These considerations clearly highlight the non-white character of the finite-size fluctuations in our theory.

### Several populations

It is straightforward to generalize the population equations to several populations by adding a population label *α* = 1, …, *M*. For the sake of completeness, we explicitly state the full set of equations. The activity of population *α* is given by

$$\begin{array}{c} A_{N}^{\alpha }\left(t\right)=\frac{1}{N^{\alpha }}\sum_{k}\delta \left(t-t_{\text{pop},k}^{\alpha }\right), \end{array}$$ (72)

where ${\left(t_{\text{pop},k}^{\alpha }\right)}_{k\in Z}$ is a point process with conditional intensity $\lambda_{\text{pop}}^{\alpha }\left(t|H_{t}\right)=N^{\alpha }{\bar{A}}^{\alpha }\left(t\right)$. Here, the expected activity $\bar{A}\left(t\right)$ depends explicitly on the history $H_{t}={\left\{A_{N}^{\beta }\left(t^{\prime }\right)\right\}}_{t^{\prime }<t,\beta =1,\ldots ,M}$ by the following set of equations

$$\begin{array}{c} {\bar{A}}^{\alpha }\left(t\right)=\int_{-\infty }^{t}\lambda^{\alpha }\left(t|\hat{t}\right)S^{\alpha }\left(t|\hat{t}\right)A_{N}^{\alpha }\left(\hat{t}\right)\;d\hat{t}+\Lambda^{\alpha }\left(t\right)\left(1-\int_{-\infty }^{t}S^{\alpha }\left(t|\hat{t}\right)A_{N}^{\alpha }\left(\hat{t}\right)\;d\hat{t}\right) \end{array}$$ (73)

$$\begin{array}{c} \lambda^{\alpha }\left(t|\hat{t}\right)=c^{\alpha }\;\exp\left(\frac{u^{\alpha }\left(t,\hat{t}\right)-\vartheta^{\alpha }\left(t,\hat{t}\right)}{\Delta_{u}^{\alpha }}\right),\;\Lambda^{\alpha }\left(t\right)=\frac{\int_{-\infty }^{t}\lambda^{\alpha }\left(t|\hat{t}\right)v^{\alpha }\left(t,\hat{t}\right)\;d\hat{t}}{\int_{-\infty }^{t}v^{\alpha }\left(t,\hat{t}\right)\;d\hat{t}}, \end{array}$$ (74)

$$\begin{array}{c} \frac{\partial S^{\alpha }}{\partial t}=-\lambda^{\alpha }\left(t|\hat{t}\right)S^{\alpha },\;S^{\alpha }\left(\hat{t}|\hat{t}\right)=1, \end{array}$$ (75)

$$\begin{array}{c} \frac{\partial v^{\alpha }}{\partial t}=-2\lambda^{\alpha }\left(t|\hat{t}\right)v^{\alpha }+\lambda^{\alpha }\left(t|\hat{t}\right)S^{\alpha }\left(t|\hat{t}\right)A_{N}^{\alpha }\left(\hat{t}\right),\;v^{\alpha }\left(\hat{t},\hat{t}\right)=0, \end{array}$$ (76)

$$\begin{array}{c} \frac{\partial u^{\alpha }}{\partial t}=-\frac{u^{\alpha }-\mu^{\alpha }\left(t\right)}{\tau_{\text{m}}^{\alpha }}+\sum_{\beta =1}^{M}w^{\alpha \beta }p^{\alpha \beta }N^{\beta }\left(\epsilon^{\alpha \beta }*A_{N}^{\beta }\right)\left(t\right),\;u^{\alpha }\left(\hat{t},\hat{t}\right)=u_{\text{r}} \end{array}$$ (77)

$$\begin{array}{c} \vartheta^{\alpha }\left(t,\hat{t}\right)=u_{\text{th}}+\theta^{\alpha }\left(t-\hat{t}\right)+\int_{-\infty }^{\hat{t}}{\tilde{\theta }}^{\alpha }\left(t-t^{\prime }\right)A_{N}^{\alpha }\left(t^{\prime }\right)dt^{\prime }. \end{array}$$ (78)

For each population, the system of Eqs (73)–(78) contains a family of ordinary differential equations for the variables *S*, *u* and *v* parametrized by the continuous parameter $\hat{t}$ with $-\infty <\hat{t}<t$, and five integrals over this parameter. In the next section, we show that the family of ordinary differential equations is equivalent to three first-order partial differential equations. Furthermore, in Sec. “Population equations for a finite history”, we reduce the infinite integrals to integrals over a finite range, which will be useful for the numerical implementation of the population equations.

### Refractory density representation

There is an equivalent formulation of the population equation in terms of first-order partial differential equations for the density of ages $\tau =t-\hat{t}$ [23, 68, 88, 89]. The representation in terms of age *τ* as a state variable is useful because it parallels the Fokker-Planck formalism for the membrane potential density [14, 36, 58–60] or related density equations [117, 118], in which the state variable is the membrane potential of a neuron. To keep the notation simple we consider in the following population *α* but drop the index *α* wherever confusion is not possible. Thus, we write e.g. *S* for *S*^*α*^ and *A*_*N*_ for $A_{N}^{\alpha }$ but we keep the index *β* as well as double indices *αβ* occurring in Eq (77).

The density of ages at time *t* is defined as *q*(*t*, *τ*) = *S*(*t*|*t* − *τ*)*A*_*N*_(*t* − *τ*). We recall that because of finite-size fluctuations, *q* is not a normalized probability density. Furthermore, we regard the functions λ, *u* and *v* as functions of *t* and *τ*. With these definitions the population equation, Eq (65), can be rewritten as

$$\begin{array}{c} \bar{A}\left(t\right)=\int_{0}^{\infty }\lambda \left(t,\tau \right)q\left(t,\tau \right)\;d\tau +\Lambda \left(t\right)\left(1-\int_{0}^{\infty }q\left(t,\tau \right)\;d\tau \right). \end{array}$$ (79)

The stochastic activity *A*_*N*_(*t*) then follows from Eqs (14) or (15). Eq (79) yields the expected population rate at time *t* for a given density of ages. In the Fokker-Planck formalism, this would correspond to the calculation of the rate from the membrane potential density as the probability flux across the threshold.

Noting that $\partial_{t}S\left(t|\hat{t}\right)A_{N}\left(\hat{t}\right)=\left(\partial_{t}+\partial_{\tau }\right)q\left(t,\tau \right)$, we find from Eq (67) the following first-order partial differential equation for the density of ages *q*(*t*, *τ*):

$$\begin{array}{c} \left(\partial_{t}+\partial_{\tau }\right)q=-\lambda \left(t,\tau \right)q,\;q\left(t,0\right)=A_{N}\left(t\right). \end{array}$$ (80)

Similarly, *u* and *v* obey from Eqs (77) and (76), respectively,

$$\begin{array}{c} (\partial_{t}+\partial_{\tau })u=-\frac{u-\mu }{\tau_{\text{m}}}+\sum_{\beta =1}^{M}w^{\alpha \beta }p^{\alpha \beta }N^{\beta }\left(\epsilon^{\alpha \beta }*A_{N}^{\beta }\right)\left(t\right), \end{array}$$ (81)

$$\begin{array}{c} (\partial_{t}+\partial_{\tau })v=-\lambda (t,\tau )[2v-q] \end{array}$$ (82)

with boundary conditions *u*(*t*, 0) = *u*_r_ and *v*(*t*, 0) = 0. These functions, together with the threshold dynamics

$$\begin{array}{c} \vartheta \left(t,\tau \right)=u_{\text{th}}+\theta \left(\tau \right)+\int_{\tau }^{\infty }\tilde{\theta }\left(\tau^{\prime }\right)A_{N}\left(t-\tau^{\prime }\right)\;d\tau^{\prime }, \end{array}$$ (83)

determine λ(*t*, *τ*) and Λ(*t*) via Eq (74), i.e.

$$\begin{array}{c} \lambda \left(t,\tau \right)=c\;\exp\left(\frac{u(t,\tau )-\vartheta (t,\tau )}{\Delta_{u}}\right),\;\Lambda \left(t\right)=\frac{\int_{0}^{\infty }\lambda \left(t,\tau \right)v\left(t,\tau \right)\;d\tau }{\int_{0}^{\infty }v\left(t,\tau \right)\;d\tau }. \end{array}$$ (84)

The Eqs (75)–(77) of the previous section can be regarded as the characteristic equations corresponding to the partial differential Eqs (80)–(82) (“method of characteristics”).

### Population equations for a finite history

To simulate the population activity forward in time, the integrals in Eq (65) over the past need to be evaluated, starting at $\hat{t}=-\infty$. For biological systems, however, it is sufficient to limit the integrals to a finite history of length *T*. This history corresponds to the range $t-T\leq \hat{t}<t$, where we have to explicitly account for the dependence of the conditional firing rate $\lambda (t|\hat{t})$ on the last spike time $\hat{t}$. We will call neurons with last spike time in this range “refractory” because they still experience some degree of (relative) refractoriness caused by the last spike. The remaining part of the integral corresponding to the range $-\infty <\hat{t}<t-T$ receives a separate, compact evaluation. We will refer to neurons with their last spike time in this range as “free” because their conditional intensity is free of the influence of the last spike.

How should we choose the length of the explicit history *T*? First of all, this length can be different for different populations and is mainly determined by the time scale of refractoriness, i.e. the time it needs to forget the individual effect of a single spike in the past. Furthermore, it depends on the properties of the spike-triggered kernel, i.e. the dynamic threshold that is responsible for adaptation. More precisely, we determine the length of the history by the following conditions: first, the conditional intensity is insensitive to the precise timing of the last spike at $\hat{t}<t-T$ if

$$\begin{array}{c} T\gg \max[t_{\text{ref}},\tau_{\text{rel}}]. \end{array}$$ (85)

Here, *t*_ref_ is the absolute refractory period and *τ*_rel_ is the time scale of the relative refractory period. For the GIF model *τ*_rel_ = *τ*_m_. Second, we demand that *T* is chosen such that for *t* > *T*, the quasi-renewal kernel $\tilde{\theta }\left(t\right)=\Delta_{u}[1-e^{-\theta \left(t\right)/\Delta_{u}}]$ can be well approximated by the original spike-triggered kernel *θ*(*t*). Taylor expansion of the exponential yields the condition

$$\begin{array}{c} \theta \left(t\right)\ll \Delta_{u},\;\forall t>T. \end{array}$$ (86)

The length of the history *T* needs to be chosen such that both conditions, Eqs (85) and (86) are fulfilled. It is important to note that condition Eq (86) does not require the time window *T* to be larger than the largest time scale of the spike-triggered kernel. For instance, consider the kernel $\theta \left(t\right)=\frac{J_{\theta }}{\tau_{\theta }}e^{-t/\tau_{\theta }}$, where *J*_*θ*_ and *τ*_*θ*_ are adaptation strength and time scale, respectively. In particular, the adaptation strength *J*_*θ*_ sets the reduction in firing rate compared to a non-adapting neuron in the limit of strong drive irrespective of the time scale *τ*_*θ*_ (see e.g. [80, 82]). Condition Eq (86) can be fulfilled for a given *T* if either *τ*_*θ*_ is small enough such that the exponential $e^{-t/\tau_{\theta }}$ is small, or, for a fixed adaptation strength *J*_*θ*_, by increasing the adaptation time scale *τ*_*θ*_ such that *J*_*θ*_/*τ*_*θ*_ ≪ Δ_*u*_.

#### Dynamic threshold of refractory and free neurons

For free neurons, i.e. for $-\infty <\hat{t}<t-T$, we use the average threshold under the assumption that spikes occurred in the range $-\infty <\hat{t}<t-T$ according to an inhomogeneous Poisson process with rate $A_{N}\left(\hat{t}\right)$. This average is given by [63, 134]

$$\begin{array}{cc} \vartheta_{\text{free}}\left(t\right) & =u_{\text{th}}+\int_{-\infty }^{t-T}\tilde{\theta }\left(t-t^{\prime }\right)A_{N}\left(t^{\prime }\right)dt^{\prime },\\ & \approx u_{\text{th}}+\int_{-\infty }^{t-T}\theta \left(t-t^{\prime }\right)A_{N}\left(t^{\prime }\right)dt^{\prime }, \end{array}$$ (87)

where in the last step we used Eq (86). We assume that for *t* > *T* the spike-triggered kernel can be sufficiently well approximated by a sum of exponentials

$$\begin{array}{c} \theta \left(t\right)=\Theta \left(t\right)\sum_{\ell =1}^{N_{\theta }}\frac{J_{\theta ,\ell }}{\tau_{\theta ,\ell }}e^{-t/\tau_{\theta ,\ell }}. \end{array}$$

This allows us to express the threshold for free neurons as

$$\begin{array}{c} \vartheta_{\text{free}}\left(t\right)=u_{\text{th}}+\sum_{\ell =1}^{N_{\theta }}J_{\theta ,\ell }e^{-T/\tau_{\theta ,\ell }}g_{\ell }\left(t\right), \end{array}$$ (88)

where the variables *g*_ℓ_(*t*) satisfy the differential equations

$$\begin{array}{c} \tau_{\theta ,\ell }\frac{dg_{\ell }}{dt}=-g_{\ell }+A_{N}\left(t-T\right). \end{array}$$ (89)

For refractory neurons, i.e. if $t-T\leq \hat{t}<t$, we need to evaluate in the effective threshold, Eq (33), an integral over the exact quasi-renewal kernel $\tilde{\theta }\left(t\right)$. Splitting this integral into the free and refractory part yields the threshold of refractory neurons:

$$\begin{array}{c} \vartheta \left(t,\hat{t}\right)=\vartheta_{\text{free}}\left(t\right)+\theta \left(t-\hat{t}\right)+\int_{t-T}^{\hat{t}}\tilde{\theta }\left(t-t^{\prime }\right)A_{N}\left(t^{\prime }\right)dt^{\prime }. \end{array}$$ (90)

We can use the threshold for free and refractory neurons, Eqs (88), (89) and (90), respectively, to obtain the respective conditional intensities:

$$\begin{array}{c} \lambda_{\text{free}}\left(t\right)=f\left(h\left(t\right)-\vartheta_{\text{free}}\left(t\right)\right),\;\lambda \left(t,\hat{t}\right)=f\left(u\left(t,\hat{t}\right)-\theta \left(t,\hat{t}\right)\right), \end{array}$$ (91)

where *h*(*t*) is the free membrane potential given by Eq (27). Let us remind the reader that *h*(*t*) obeys the dynamics Eq (23) but without resetting of the membrane potential after a spike.

#### Population equations

We now apply the split of the history to the integrals that appear in the population equations, specifically Eqs (65) and (66). By definition, the conditional intensity of free neurons does not depend explicitly on the last spike time $\hat{t}$. That is, $\lambda \left(t|\hat{t}\right)=\lambda_{\text{free}}\left(t\right)$ for $-\infty <\hat{t}<t-T$, where the free hazard rate λ_free_(*t*) is given by Eq (91). In the free part of the integrals in Eqs (65) and (66), the free hazard rate can be pulled out of the integral, which yields

$$\begin{array}{c} \bar{A}\left(t\right)=\int_{t-T}^{t}\lambda \left(t|\hat{t}\right)S\left(t|\hat{t}\right)A_{N}\left(\hat{t}\right)\;d\hat{t}+\lambda_{\text{free}}\left(t\right)\frac{x(t)}{N}\\ \;+\Lambda \left(t\right)\left(1-\int_{t-T}^{t}S\left(t|\hat{t}\right)A_{N}\left(\hat{t}\right)\;d\hat{t}-\frac{x(t)}{N}\right), \end{array}$$ (92)

$$\begin{array}{c} \Lambda (t)=\frac{\int_{t-T}^{t}\lambda \left(t|\hat{t}\right)v\left(t,\hat{t}\right)\;d\hat{t}+\lambda_{\text{free}}\left(t\right)z\left(t\right)/N}{\int_{t-T}^{t}v\left(t,\hat{t}\right)\;d\hat{t}+z\left(t\right)/N}. \end{array}$$ (93)

Here we have introduced the expected number of free neurons $x\left(t\right)=N\int_{-\infty }^{t-T}S\left(t|\hat{t}\right)A_{N}\left(\hat{t}\right)\;d\hat{t}$ and the partial integral over the variance function $z\left(t\right)=N\int_{-\infty }^{t-T}v\left(t,\hat{t}\right)\;d\hat{t}$. Differentiating these new variables and employing Eqs (63) and (69), we find that they obey the differential equations

$$\begin{array}{c} \frac{dx}{dt}=-\lambda_{\text{free}}\left(t\right)x+NS\left(t|t-T\right)A_{N}\left(t-T\right), \end{array}$$ (94)

$$\begin{array}{c} \frac{dz}{dt}=-2\lambda_{\text{free}}\left(t\right)z+\lambda_{\text{free}}\left(t\right)x+Nv\left(t,t-T\right). \end{array}$$ (95)

Thus, the integrals no longer run from −∞ to *t* but are now limited to the range [*t* − *T*, *t*). The long tails over the past have been reduced to differential equations.

### Numerical implementation

#### Discretization of time

We discretize the time axis into a grid with step size Δ*t* and grid points

$$\begin{array}{c} t_{k}=k\Delta t,\;k\in Z. \end{array}$$ (96)

Because we keep track of a finite history with the oldest last spike time $\hat{t}=t-T$, the history consists of a finite number *K* of bins such that *T* = *K*Δ*t*. If the index *k* = *l* corresponds to the current time, the oldest last spike time of the explicit history corresponds to an index *k* = *l* − *K* and the most recent one corresponds to the index *k* = *l* − 1. Note that the numerical implementation requires the absolute refractory period *t*_ref_ to be at least as large as the integration time step Δ*t* (see below).

To facilitate the notation of the update rules, it is convenient to introduce the following notations:

$$\begin{array}{c} {\bar{m}}_{k}\left(t_{l}\right)\equiv \left\langle m_{l,k}\right\rangle =N\Delta tS\left(t_{i}|t_{k}\right)A_{N}\left(t_{k}\right), \end{array}$$ (97)

$$\begin{array}{c} v_{k}\left(t_{l}\right)\equiv \left\langle \Delta m_{l,k}^{2}\right\rangle =N\Delta tv\left(t_{l},t_{k}\right), \end{array}$$ (98)

$$\begin{array}{c} u_{k}\left(t_{l}\right)\equiv u(t_{l}|t_{k}), \end{array}$$ (99)

$$\begin{array}{c} \lambda_{k}\left(t_{l}\right)\equiv \lambda (t_{l}|t_{k}). \end{array}$$ (100)

In particular, ${\bar{m}}_{k}$ and *v*_*k*_ correspond to the mean and variance of the unconstrained survival numbers, respectively. We also recall that the population index *α* is dropped wherever confusion is not possible, while the index *β* as well as double indices *αβ* will be kept.

#### Choice of Δ*t*

A crucial assumption of the derivation of the population equations in discrete time was that the time step Δ*t* is small enough such that each neuron fires at most one spike per time step. This can be achieved by the condition

$$\begin{array}{c} \Delta t\leq t_{\text{ref}} \end{array}$$ (101)

(cf. Sec. “Discretized population density equations”). Clearly, this condition implies that the total number of spikes per time step must be bounded by the number of neurons, i.e. the population activity must obey Δ*n*(*t*_*l*_) ≤ *N*. The equality sign corresponds to the case that all neurons fire in the same time step. In addition to condition Eq (101), we also had to require that Δ*t* is not larger than the transmission delay Δ, i.e.

$$\begin{array}{c} \Delta t\leq \Delta . \end{array}$$ (102)

In order to justify the use of the Poisson statistics in the derivation of the population equations, we further assumed that Δ*t* is sufficiently small such that the expected number of spikes per time step, $\Delta \bar{n}\left(t\right)$, is much smaller than *N*, or equivalently $\bar{A}\left(t\right)\Delta t⪡1$. While this does not pose a problem for the theory, which ultimately concerns with the continuum limit Δ*t* → 0, an efficient numerical integration of the population equations benefits from a time step that is as large as possible and should thus not be limited by such a condition. In particular, we should allow a large fraction of neurons to fire during one time step, either as a result of an external synchronization of many neurons (e.g. by a strong, sudden stimulus) or because of synchronous oscillations emerging from the network dynamics. In this case, a Poisson-distributed spike count Δ*n*(*t*) may exceed the number of neurons *N*. This problem can be remedied by drawing Δ*n*(*t*) from a binomial distribution with mean $\Delta \bar{n}\left(t\right)$ and maximal value *N*. For $\Delta \bar{n}\left(t\right)⪡N$, this binomial distribution agrees with the Poisson distribution used in our theory, whereas at large $\Delta \bar{n}\left(t\right)$ it ensures that the spike count is bounded by the total number of neurons *N*. Although the binomial distribution does not follow strictly from our theory, it is expected to yield a very good approximation even at large $\Delta \bar{n}\left(t\right)$. The reason is as follows: A statement analogous to our argument that the sum of Poisson numbers [Eq (39)] yields again a Poisson number [Eq (46)] is, in general, not valid for binomial random numbers if the random numbers (i.e. the firing probabilities *P*_λ_(*t*_*l*_|*t*_*k*_) in our model) are very different. However, if neurons are strongly synchronized, and hence $\Delta \bar{n}\left(t\right)∼N$, they fire with a similar probability, which implies indeed a binomial distribution of the spike count Δ*n*(*t*).

Besides Eqs (101) and (102), a third condition concerns the approximation of the integral $\int_{t}^{t+\Delta t}\lambda \left(t^{\prime }\right)dt^{\prime }$ in Eq (37) by $\bar{\lambda }\left(t\right)\Delta t$ (trapezoidal rule). This approximation is valid if the membrane potential *u* and threshold *ϑ* do not vary too strongly during a time step. More precisely, the absolute error of the trapezoidal rule is known to be Δ*t*^3^|λ′′|/12, which we require to be much smaller than λΔ*t*. Thus, the relative error is of order Δ*t*^2^. Using the definition of λ, Eq (25), this leads to the condition

$$\begin{array}{c} \left|{\left(\frac{\partial u}{\partial t}-\frac{\partial \vartheta }{\partial t}\right)}^{2}+\Delta_{u}\left(\frac{\partial^{2}u}{\partial t^{2}}-\frac{\partial^{2}\vartheta }{\partial t^{2}}\right)\right|\Delta t^{2}\ll 12\Delta_{u}^{2}. \end{array}$$ (103)

In summary, Δ*t* should be chosen such that all three conditions, Eqs (101)–(103) are satisfied for all populations.

#### Update of the membrane potential

To compute the firing probabilities, we need to update both the membrane potential and the threshold. In the presence of an exponential synaptic filter $\epsilon^{\alpha \beta }\left(s\right)=\Theta \left(s-\Delta \right)e^{-\left(s-\Delta \right)/\tau_{\text{s}}^{\beta }}/\tau_{\text{s}}^{\beta }$, the membrane potential of free neurons *u*(*t*) = *h*(*t*) in population *α* obeys the differential equation

$$\begin{array}{c} \tau_{\text{m}}\frac{dh}{dt}=-h+u_{\text{rest}}+RI_{\text{ext}}\left(t\right)+\tau_{\text{m}}\sum_{\beta =1}^{M}p^{\alpha \beta }N^{\beta }w^{\alpha \beta }y^{\alpha \beta }, \end{array}$$ (104)

$$\begin{array}{cc} \tau_{\text{s}}^{\beta }\frac{dy^{\alpha \beta }}{dt}=-y^{\alpha \beta }+A_{N}^{\alpha \beta }\left(t-\Delta \right), & \;\;\beta =1,\ldots ,M. \end{array}$$ (105)

Assuming that the external stimulus *I*_ext_(*t*) and the population activity *A*_*N*_(*t*) are constant during one time step, the solution over one time step is given by

$$\begin{array}{c} h(t_{l+1})=u_{\text{rest}}+\left(h\left(t_{l}\right)-u_{\text{rest}}\right)e^{-\Delta t/\tau_{\text{m}}}+h_{\text{tot}}, \end{array}$$ (106)

$$\begin{array}{cc} y^{\alpha \beta }\left(t_{l+1}\right)=A_{N}^{\beta }\left(t_{l}-\Delta \right)+\left[y^{\alpha \beta }\left(t_{l}\right)-A_{N}^{\beta }\left(t_{l}-\Delta \right)\right]e^{-\Delta t/\tau_{\text{s}}^{\beta }}, & \beta =1,\ldots ,M \end{array}$$ (107)

where *h*_tot_ is the total input of population *α* given by

htot=RIext(tl)1-e-Δtτm+τm∑β=1MpαβNβwαβANβ(tl-Δ)+τsβe-Δtτsβyαβ(tl)-ANβ(tl-Δ)τsβ-τm+τsβe-Δtτsβyαβ(tl)-ANβ(tl-Δ)-e-Δtτmτsβyαβ(tl)-τmANβ(tl-Δ)τsβ-τm (108)

For refractory neurons, we obtain the membran potential in the GLM model by the simple formula *u*_*k*_(*t*_*l*+1_) = *h*(*t*_*l*+1_) + *η*(*t*_*l*+1_ − *t*_*k*_). For the GIF model, the same update rule as for *h*(*t*), Eq (106), can be applied for *k* = *l* − *K*, …, *l* − *k*_ref_:

$$\begin{array}{c} u_{k}\left(t_{l+1}\right)=u_{\text{rest}}+\left(u_{k}\left(t_{l}\right)-u_{\text{rest}}\right)e^{-\Delta t/\tau_{\text{m}}}+h_{\text{tot}}. \end{array}$$ (109)

For the absolute refractory period, *l* − *k*_ref_ < *k* < *l*, the membrane potential remains at *u*_*k*_(*t*_*l*+1_) = *u*_r_. Note that the total integrated input *h*_tot_ needs to be computed only once per time step.

#### Update of the threshold

Let us first discuss, how to compute the threshold at time *t*_*l*_ given the values of *g*_ℓ_(*t*_*l*_) and Δ*n*(*t*_*k*_) for *k* = *l* − *K*, …, *l* − 1. For free neurons, the threshold *ϑ*_free_(*t*_*l*_) is given by Eq (88) evaluated at time *t* = *t*_*l*_. For refractory neurons, we find from Eq (90) that the threshold can be written in the discretized form

$$\begin{array}{c} \vartheta_{k}\left(t_{l}\right)=\vartheta_{\text{free}}\left(t_{l}\right)+\theta \left(t_{l}-t_{k}\right)+\frac{1}{N}\sum_{k^{\prime }=l-K}^{k-1}\tilde{\theta }\left(t_{l}-t_{k^{\prime }}\right)\Delta n\left(t_{k^{\prime }}\right), \end{array}$$ (110)

*k* = *l* − *K*, …, *l* − *k*_ref_. Eq (110) can be rewritten as

$$\begin{array}{c} \vartheta_{k}\left(t_{l}\right)={\hat{\vartheta }}_{k}\left(t_{l}\right)+\theta \left(t_{l}-t_{k}\right), \end{array}$$ (111)

where the variables ${\hat{\vartheta }}_{k}\left(t\right)$ can be calculated iteratively starting at *k* = *l* − *K*:

$$\begin{array}{c} {\hat{\vartheta }}_{k+1}\left(t_{l}\right)={\hat{\vartheta }}_{k}\left(t_{l}\right)+N^{-1}\tilde{\theta }\left(t_{l}-t_{k}\right)\Delta n\left(t_{k}\right),\;k=l-K,\ldots ,l-1-k_{\text{ref}} \end{array}$$ (112)

with initial condition ${\hat{\vartheta }}_{l-K}\left(t_{l}\right)=\vartheta_{\text{free}}\left(t_{l}\right)$. Thus, at each time step *t*_*l*_, the threshold can be rapidly evaluated in one sweep via Eqs (88), (111) and (112).

For the computation of the firing probabilities below, it is necessary to compute the threshold one time step ahead, i.e. at time *t*_*l*+1_. To this end, we first update the variables *g*_ℓ_ according to Eq (89):

$$\begin{array}{c} g_{\ell }\left(t_{l+1}\right)=g_{\ell }\left(t_{l}\right)e^{-\Delta t/\tau_{\theta ,\ell }}+\frac{\Delta n(t_{l-K})}{N\Delta t}\left(1-e^{-\Delta t/\tau_{\theta ,\ell }}\right). \end{array}$$ (113)

This yields the threshold *ϑ*_free_(*t*_*l*+1_) of free neurons via the formula Eq (88). For refractory neurons we find from Eqs (111) and (112)

$$\begin{array}{c} \vartheta_{k}\left(t_{l+1}\right)={\hat{\vartheta }}_{k}\left(t_{l+1}\right)+\theta \left(t_{l+1}-t_{k}\right), \end{array}$$ (114)

where ${\hat{\vartheta }}_{k}\left(t_{l+1}\right)$ can be iterated by

$$\begin{array}{c} {\hat{\vartheta }}_{k+1}\left(t_{l+1}\right)={\hat{\vartheta }}_{k}\left(t_{l+1}\right)+N^{-1}\tilde{\theta }\left(t_{l+1}-t_{k}\right)\Delta n\left(t_{k}\right),\;k=l-K,\ldots ,l-k_{\text{ref}}-1. \end{array}$$ (115)

with initial condition

$$\begin{array}{c} {\hat{\vartheta }}_{l-K}\left(t_{l+1}\right)=\vartheta_{\text{free}}\left(t_{l+1}\right)-N^{-1}\tilde{\theta }\left(t_{l+1}-t_{l-K}\right)\Delta n\left(t_{l-K}\right). \end{array}$$ (116)

#### Firing probabilities

The firing probabilities for free and refractory neurons are given by

$$\begin{array}{c} P_{\text{free}}\left(t_{l}\right)=1-e^{-{\bar{\lambda }}_{\text{free}}\left(t_{l}\right)\Delta t},\;P_{\lambda }\left(t_{l}|t_{k}\right)=1-e^{-\bar{\lambda }\left(t_{l}|t_{k}\right)\Delta t}, \end{array}$$ (117)

respectively. Here,

$$\begin{array}{c} {\bar{\lambda }}_{\text{free}}\left(t_{l}\right)=[\lambda_{\text{free}}\left(t_{l}\right)+\lambda_{\text{free}}\left(t_{l+1}\right)]/2, \end{array}$$ (118)

$$\begin{array}{c} \bar{\lambda }\left(t_{l}|t_{k}\right)=[\lambda_{k}\left(t_{l}\right)+\lambda_{k}\left(t_{l+1}\right)]/2, \end{array}$$ (119)

are the arithmetic mean of the respective intensities at the beginning and end of the time interval (cf. Eq (37)). The free intensity λ_free_(*t*) is given by Eq (91). For refractory neurons, the conditional intensities are given by

$$\begin{array}{c} \lambda_{k}\left(t\right)=\left\{\begin{array}{cc} f\left(u_{k}\left(t\right)-\vartheta_{k}\left(t\right)\right), & t_{k}<t-k_{\text{ref}}\Delta t\\ 0, & t-k_{\text{ref}}\Delta t\leq t_{k}<t, \end{array}\right. \end{array}$$ (120)

where the last case corresponds to the absolute refractory period.

#### Population dynamics

We can directly use the discretized form of the population equations given by Eqs (58) and (59). As in Eq (92) the infinite sums in Eq (59) can be split into an explicit, finite history of length *K* and a remaining part corresponding to *k* < *l* − *K*. This results in

$$\begin{array}{c} \Delta \bar{n}\left(t_{l}\right)=\sum_{k=l-K}^{l-1}P_{\lambda }\left(t_{l}|t_{k}\right){\bar{m}}_{k}\left(t_{l}\right)+P_{\text{free}}\left(t_{l}\right)x\left(t_{i}\right)+\\ +P_{\Lambda }\left(t_{l}\right)\left(N-\sum_{k=l-K}^{l-1}{\bar{m}}_{k}\left(t_{l}\right)-x\left(t_{l}\right)\right), \end{array}$$ (121)

where

$$\begin{array}{c} P_{\Lambda }\left(t_{l}\right)=\frac{\sum_{k=l-K}^{l-1}P_{\lambda }(t_{l}|t_{k})v_{k}\left(t_{l}\right)+P_{\text{free}}\left(t_{l}\right)z\left(t_{l}\right)}{\sum_{k=l-K}^{l-1}v_{k}\left(t_{l}\right)+z\left(t_{l}\right)}. \end{array}$$ (122)

is the firing probability of “neurons” belonging to the “holes and overshoots” *δm*_*l*,*k*_ (cf. Results, Sec. “Mesoscopic population equations”). The variables *x* and *z* have the discrete time definition

$$\begin{array}{c} x\left(t_{l}\right)=\sum_{k=-\infty }^{l-K-1}{\bar{m}}_{k}\left(t_{l}\right),\;z\left(t_{l}\right)=\sum_{k=-\infty }^{l-K-1}v_{k}\left(t_{l}\right), \end{array}$$ (123)

corresponding to a discretization of their integral definition above. Having calculated the expected spike count $\Delta \bar{n}\left(t_{l}\right)$, the actual spike count Δ*n*(*t*_*l*_) is obtained by drawing a binomially distributed random number

$$\begin{array}{c} \Delta n\left(t_{l}\right)∼B\left(N,p_{B}=\Delta \bar{n}\left(t_{l}\right)/N\right) \end{array}$$ (124)

as discussed above. In Eq (124), *B*(*N*, *p*_*B*_) denotes the binomial distribution corresponding to *N* Bernoulli trials with success probability *p*_*B*_.

The discrete evolution equations for ${\bar{m}}_{k}\left(t_{l}\right)$ and *v*_*k*_(*t*_*l*_) are given by Eqs (48) and (51), respectively, which we repeat here for convenience:

$$\begin{array}{c} {\bar{m}}_{k}\left(t_{l+1}\right)=\left[1-P_{\lambda }\left(t_{l}|t_{k}\right)\right]{\bar{m}}_{k}\left(t_{l}\right) \end{array}$$ (125)

$$\begin{array}{c} v_{k}\left(t_{l+1}\right)={\left[1-P_{\lambda }\left(t_{l}|t_{k}\right)\right]}^{2}v_{k}\left(t_{l}\right)+P_{\lambda }\left(t_{l}|t_{k}\right){\bar{m}}_{k}\left(t_{l}\right). \end{array}$$ (126)

To find the update rule for *x*, we use the definition Eq (123) and the update rule for ${\bar{m}}_{k}\left(t_{l}\right)$, Eq (125):

$$\begin{array}{cc} x(t_{l+1}) & =\sum_{k=-\infty }^{l-K}{\bar{m}}_{k}\left(t_{l+1}\right)=\sum_{k=-\infty }^{l-K}\left[1-P_{\lambda }\left(t_{l}|t_{k}\right)\right]{\bar{m}}_{k}\left(t_{l}\right)\\ & =\left[1-P_{\text{free}}\left(t_{l}\right)\right]\sum_{k=-\infty }^{l-K-1}{\bar{m}}_{k}\left(t_{l}\right)+\left[1-P_{\lambda }\left(t_{l}|t_{l-K}\right)\right]{\bar{m}}_{l-K}\left(t_{l}\right). \end{array}$$ (127)

Here, we have exploited that *P*_λ_(*t*_*l*_|*t*_*k*_) = *P*_free_(*t*_*l*_) for *k* < *l* − *K*. Using again Eq (125) we find

$$\begin{array}{c} x\left(t_{l+1}\right)=\left[1-P_{\text{free}}\left(t_{l}\right)\right]x\left(t_{l}\right)+{\bar{m}}_{l-K}\left(t_{l+1}\right). \end{array}$$ (128)

This equation is the discrete analog of the continuous-time Eq (94). Note that ${\bar{m}}_{l-K}\left(t_{l+1}\right)$ is given by Eq (125).

An update rule for *z* can be found from the definition Eq (123) and the update rule for $\langle \Delta m_{l,k}^{2}\rangle$, Eq (126). A similar calculation that led to Eq (128) results in

$$\begin{array}{c} z\left(t_{l+1}\right)={\left[1-P_{\text{free}}\left(t_{l}\right)\right]}^{2}z\left(t_{l}\right)+P_{\text{free}}\left(t_{l}\right)x\left(t_{l}\right)+v_{l-K}\left(t_{l+1}\right). \end{array}$$ (129)

This equation is the discrete analog of the continuous-time Eq (95). Note that *v*_*l*−*K*_(*t*_*l*+1_) is given by Eq (126).

Finally, the initial conditions can be accounted for by setting

$$\begin{array}{c} {\bar{m}}_{l}\left(t_{l+1}\right)=\Delta n\left(t_{l}\right),\;v_{l}\left(t_{l+1}\right)=0,\;u_{l}\left(t_{l+1}\right)=u_{\text{r}}. \end{array}$$ (130)

The last update step corresponding to the reset of the membrane potential only needs to be performed for the GIF model.

#### Initialization and storage of history

One simple way to initialize the system is to fully synchronize the network at time −Δ*t* such that at time *t*_0_ = 0 all neurons are refractory. This gives rise to the sharp initial condition $\Delta n\left(t_{k}\right)={\bar{m}}_{k}\left(0\right)=N\delta_{k,-1}$ and *v*_*k*_(0) = *u*_*k*_(0) = 0 for the refractory epoch (*k* = −*K*, …, −1). Here, *δ*_*k*,*l*_ denotes the Kronecker delta, which is unity for *k* = *l* and zero otherwise. After synchronization there are no free neurons, hence *x*(*t*_0_) = *z*(*t*_0_) = 0 and, if there were no further spikes in the past, *g*_ℓ_(*t*_0_) = 0 for ℓ = 1, …, *N*_*θ*_. The initialization of *g*_ℓ_ corresponds to a zero adaptation level at the beginning of the simulation.

For the representation of the variables ${\bar{m}}_{k}\left(t_{l}\right)$, *v*_*k*_(*t*_*l*_), *u*_*k*_(*t*_*l*_) and λ_*k*_(*t*_*l*_), *k* = *l* − *K*, …, *l* − 1, in memory, it is convenient to employ circular buffers. That is, the “running” range of the explicit history *k* = *l* − *K*, …, *l* − 1 is mapped to a static range $\hat{k}=0,\ldots ,K-1$ in memory by applying the modulo operation

$$\begin{array}{c} \hat{k}=\left(k\;\text{mod}\;K\right) \end{array}$$ (131)

to all temporal indices.

#### Summary of the update step and pseudocode

Let us summarize the steps needed to evolve the population equation from time *t*_*l*_ to time *t*_*l*+1_:

1. Calculate the total integrated input *h*_tot_ using Eq (108) and then update the synaptic variables *y*^*αβ*^(*t*_*l*+1_) according to Eq (107).
2. Update the free membrane potential *h*(*t*_*l*+1_) and threshold variable *g*_ℓ_(*t*_*l*+1_) for free neurons using Eqs (106) and (113) and use these values to compute the threshold *ϑ*_free_(*t*_*l*+1_) and conditional intensity λ_free_(*t*_*l*+1_) of free neurons by means of Eqs (88) and (91). This yields the firing probability of free neurons *P*_free_(*t*_*l*_) via Eqs (117) and (118).
3. For all refractory states *k* = *l* − *K*, …, *l* − *k*_ref_, compute the membrane potential *u*_*k*_(*t*_*l*+1_) from Eq (109), the threshold *ϑ*_*k*_(*t*_*l*+1_) from Eq (115) and the conditional intensity λ_*k*_(*t*_*l*+1_) from Eq (120). The firing probabilities *P*_λ_(*t*_*l*_|*t*_*k*_) are then given by Eqs (117) and (119).
4. Calculate the effective firing probability *P*_Λ_(*t*_*l*_) from Eq (122).
5. Calculate the expected activity $\Delta \bar{n}\left(t_{l}\right)$ by Eq (121). The empirical population activity Δ*n*(*t*_*l*_) can be obtained by drawing a binomially distributed random number according to Eq (124).
6. Update the mean and variance of the survival numbers ${\bar{m}}_{k}\left(t_{l+1}\right)$, *v*_*k*_(*t*_*l*+1_), *x*(*t*_*l*+1_) and *z*(*t*_*l*+1_) using Eqs (125), (126), (128) and (129).
7. Realize the boundary conditions at $\hat{t}=t$ according to Eq (130).

**Fig 11:**
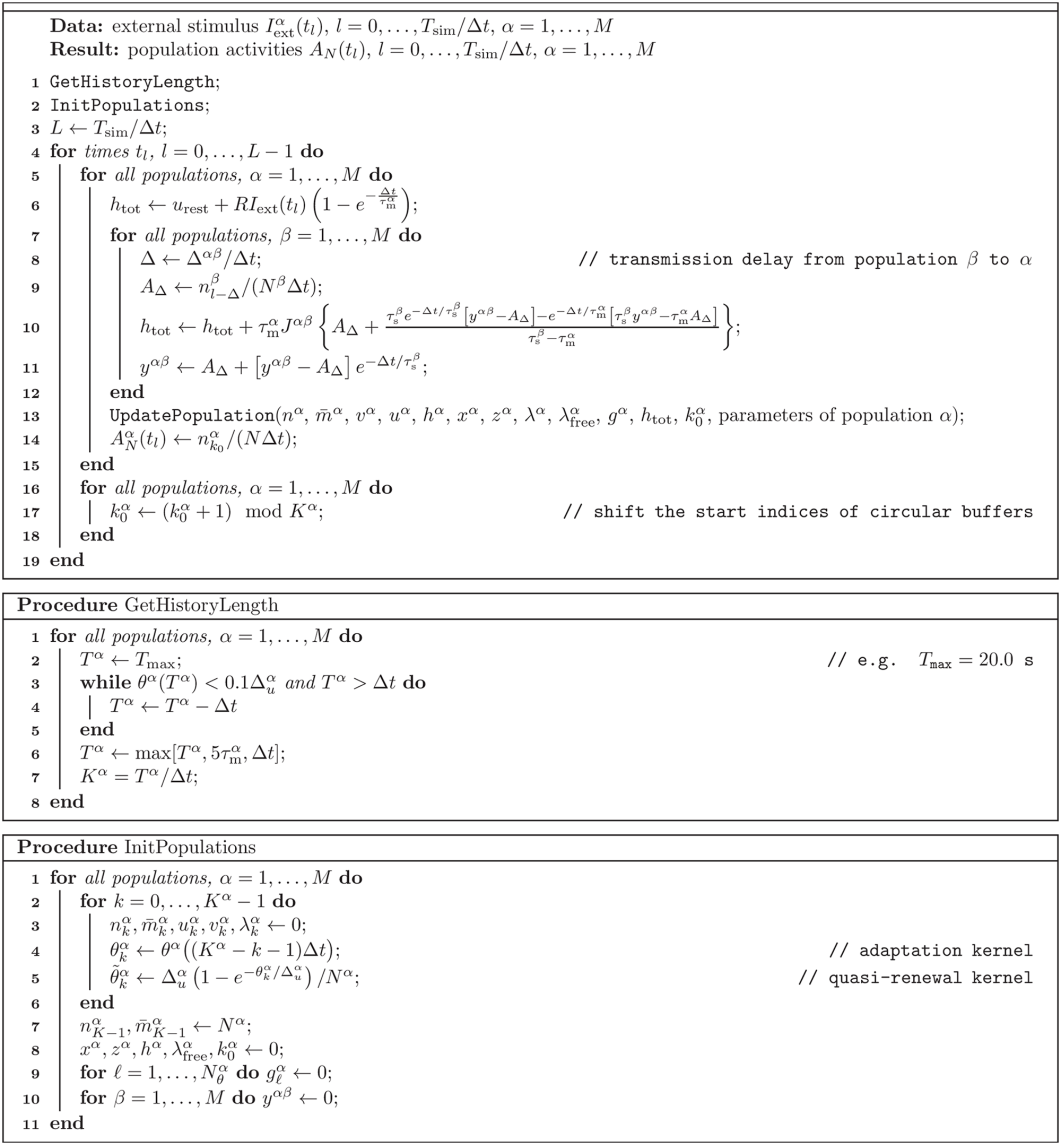
Pseudocode for the integration of the mesoscopic population equation. Note that procedure UpdatePopulation in line 12 is shown in Fig 12.

**Fig 12:**
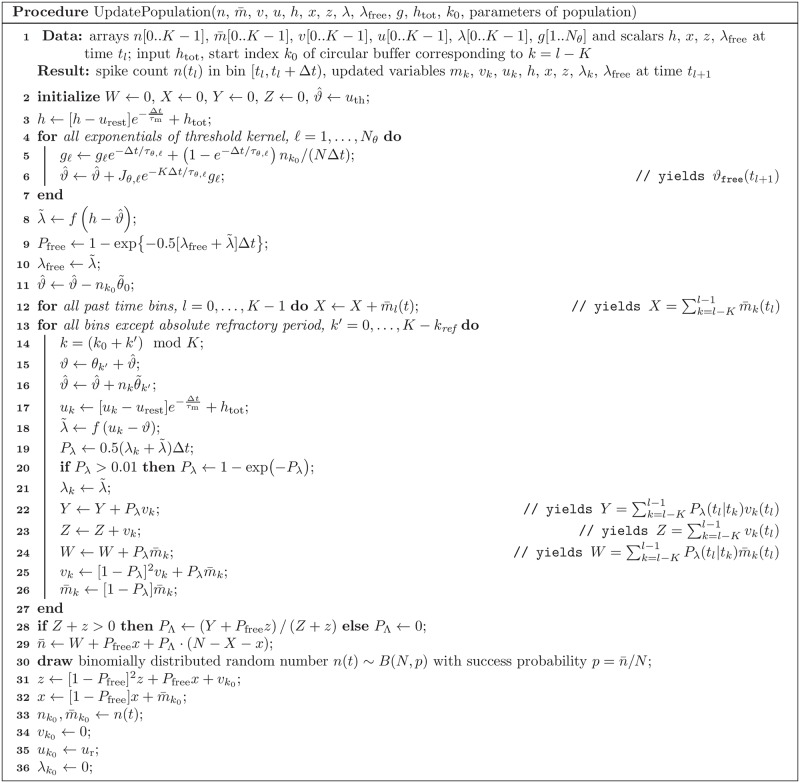
Pseudocode for the update of the variables of a given population. Note that the adaptation kernel *θ*_*k*′_ ≡ *θ*((*K* − *k*′)Δ*t*), the quasi-renewal kernel ${\tilde{\theta }}_{k^{\prime }}\equiv \tilde{\theta }((K-k^{\prime })\Delta t)/N$, Eq (33), as well as the exponentials $e^{-\frac{\Delta t}{\tau_{\text{m}}}}$ and $J_{\theta ,\ell }e^{-K\Delta t/\tau_{\theta ,\ell }}$ can be precomputed.

These steps have to be performed for all populations *α* = 1, …, *M*. A detailed implementation of the algorithm is provided by the pseudocode shown in Figs 11 and 12.

Whereas the complexity of the microscopic simulation is of order *O*(*N*Δ*t*^−1^), the integration of the population equation is of order *O*(Δ*t*^−2^) because in each time step one has to update a history of length *K* = *T*/Δ*t* (step 3, 4 and 6). Hence, at low neuron numbers (e.g. *N* < 100), the direct simulation of the microscopic system may become more efficient. We emphasize, however, that to achieve a comparable accuracy, the integration of the mesoscopic population equation can be performed on a coarser, millisecond time scale (e.g., Δ*t* = 1 ms), whereas the microscopic simulation requires precise spike times and hence a sub-millisecond simulation (e.g., Δ*t* = 0.1 ms). If we take advantage of this fact, the mesoscopic population model performs well even at low neuron numbers.

### Power spectrum

We characterize the fluctuations of the stationary population activity by the power spectrum defined as

$$\begin{array}{c} \tilde{C}\left(f\right)=\lim_{T\to \infty }\frac{\left\langle |\tilde{A}\left(f;T\right){|}^{2}\right\rangle }{T}, \end{array}$$ (132)

where

$$\begin{array}{c} \tilde{A}\left(f;T\right)=\int_{0}^{T}A_{N}\left(t\right)e^{2\pi ift}\;dt \end{array}$$ (133)

is the Fourier transform of the population activity on a time window of length *T*.

For a population of renewal neurons the power spectrum is known analytically. It is given by [134]

$$\begin{array}{c} \tilde{C}\left(f\right)=\frac{r}{N}\frac{1-|{\tilde{P}}_{\text{ISI}}{\left(f\right)|}^{2}}{|1-{\tilde{P}}_{\text{ISI}}{\left(f\right)|}^{2}}, \end{array}$$ (134)

where ${\tilde{P}}_{\text{ISI}}\left(f\right)$ is the Fourier transform of the interspike interval density

$$\begin{array}{c} P_{\text{ISI}}\left(t\right)=\lambda \left(t|0\right)\exp\left(-\int_{0}^{t}\lambda \left(s|0\right)\;ds\right) \end{array}$$ (135)

and *r* is the stationary firing rate given by

$$\begin{array}{c} r={\left[\int_{0}^{\infty }\exp\left(-\int_{0}^{t}\lambda \left(s|0\right)\;ds\right)\;dt\right]}^{-1}. \end{array}$$ (136)

Note that the power of the fluctuations in Eq (134) scales like 1/*N*, vanishing in the macroscopic limit *N* → ∞. For the LIF model with escape noise, the hazard rate λ(*t*|0) is given by

$$\begin{array}{c} \lambda \left(t|0\right)=f\left(u\left(t,0\right)-u_{\text{th}}\right),\;u\left(t,0\right)=\mu +\left(u_{\text{r}}-\mu \right)\exp\left(-\frac{t-t_{\text{ref}}}{\tau_{\text{m}}}\right) \end{array}$$ (137)

for *t* > *t*_ref_ and λ(*t*|0) = 0 for *t* ≤ *t*_ref_.

### Modified Potjans-Diesmann model

To model the cortical column of [5] in our framework, we used the parameters of the original publication and modified the model in two ways: First, the background Poisson input was replaced by a constant drive and an increased escape noise such that the populations exhibited roughly the same stationary firing rates. Specifically, we set *u*_r_ = 0 mV, *u*_th_ = 15 mV andΔ_*u*_ = 5 mV; and, using the mesoscopic dynamics, fitted the resting potentials of the GIF model (here denoted by ${\hat{\mu }}^{\alpha }$) without adaptation *J*_*θ*_ = 0 to obtain firing rates $\hat{r}$ that roughly match the target firing rates. Second, we introduced adaptation on excitatory cells with strength *J*_*θ*_ and time scale *τ*_*θ*_, and re-adjusted the resting potential as follows $u_{\text{rest}}=\hat{\mu }+J_{\theta }\hat{r}$. This yielded correct stationary firing rates in the presence of adaptation. The resulting parameters of the modified model are summarized in Table 2.

**Table 2:** Parameters of the modified Potjans-Diesmann model.

population	L2/3e	L2/3i	L4e	L4i	L5e	L5i	L6e	L6i
*τ*_m_ [s]	0.01							
*τ*_s_ [s]	0.0005							
Δ [s]	0.0015							
*t*_ref_ [s]	0.002							
Δ_*u*_ [mV]	5.0							
$\hat{\mu }$ [mV]	19.149	20.362	30.805	28.069	29.437	29.33	34.932	32.081
$\hat{r}$ [Hz]	0.974	2.861	4.673	5.65	8.141	9.013	0.988	7.53
adaptation: $\theta \left(t\right)=\left(J_{\theta }/\tau_{\theta }\right)e^{-t/\tau_{\theta }}$ for *t* > *t*_ref_								
*J*_*θ*_ [mV s]	1.0	0.0	1.0	0.0	1.0	0.0	1.0	0.0
*τ*_*θ*_ [s]	1.0	-	1.0	-	1.0	-	1.0	-
$u_{\text{rest}}=\hat{\mu }+J_{\theta }\hat{r}$ [mV]	20.123	20.362	35.478	28.069	37.578	29.33	35.92	32.081
step stimulus (“thalamic input”)								
*RI*_ext_ [mV]	0.	0.	19.	11.964	0.	0.	9.896	3.788
*μ*(*t*) [mV]	*u*_rest_ + *RI*_ext_ for *t* ∈ [0.06*s*, 0.09*s*], else *u*_rest_							
network parameters								
*N*	20683	5834	21915	5479	4850	1065	14395	2948
connection prob. *p*^*αβ*^	from population *β*							
*α* = L2/3e	0.1009	0.1689	0.0437	0.0818	0.0323	0.0	0.0076	0.0
*α* = L2/3i	0.1346	0.1371	0.0316	0.0515	0.0755	0.0	0.0042	0.0
*α* = L4e	0.0077	0.0059	0.0497	0.135	0.0067	0.0003	0.0453	0.0
*α* = L4i	0.0691	0.0029	0.0794	0.1597	0.0033	0.0	0.1057	0.0
*α* = L5e	0.1004	0.0622	0.0505	0.0057	0.0831	0.3726	0.0204	0.0
*α* = L5i	0.0548	0.0269	0.0257	0.0022	0.06	0.3158	0.0086	0.0
*α* = L6e	0.0156	0.0066	0.0211	0.0166	0.0572	0.0197	0.0396	0.2252
*α* = L6i	0.0364	0.001	0.0034	0.0005	0.0277	0.008	0.0658	0.1443
*w*^*αβ*^ [mV], *α* = L4e	0.176	-0.702	0.351	-0.702	0.176	-0.702	0.176	-0.702
*w*^*αβ*^ [mV], *α*≠ L4e	0.176	-0.702	0.176	-0.702	0.176	-0.702	0.176	-0.702
